# The varying temperature- and pressure-induced phase transition pathways in hybrid improper ferroelectric Sr_3_Sn_2_O_7_

**DOI:** 10.1107/S2052520625002306

**Published:** 2025-04-14

**Authors:** Evie Ladbrook, Jeremiah P. Tidey, Fei-Ting Huang, Sang-Wook Cheong, Dominik Daisenberger, Mark R. Warren, Mark S. Senn

**Affiliations:** a Department of Chemistry University of Warwick Gibbet Hill Coventry CV4 7AL United Kingdom; b Department of Physics University of Warwick Gibbet Hill Coventry CV4 7AL United Kingdom; c Keck Center for Quantum Magnetism, Rutgers University, Piscataway, New Jersey, 07928, USA; d Diamond Light Source Ltd Harwell Science and Innovation Campus Didcot OX11 0DE United Kingdom; Moscow State University, Russian Federation

**Keywords:** high-pressure crystallography, hybrid improper ferroelectricity, Ruddlesden–Popper

## Abstract

A variable-temperature and pressure single-crystal diffraction study of hybrid improper ferroelectric Sr_3_Sn_2_O_7_ is reported. In combination with symmetry analysis, we reveal that the application of pressure and temperature induce distinct phase transition pathways, driven by a differing response of the octahedral rotations to these stimuli. Contrary to what has been previously predicted, we observe the ferroelectric to paraelectric phase transition between 10.17(18) and 12.13(14) GPa, meaning the hybrid improper ferroelectric phase remains stable to significantly higher pressures than expected.

## Abstract 2

The phase transitions in Sr_3_Sn_2_O_7_ are studied crystallographically, illustrating both the hybrid improper ferroelectric mechanism that acts in this material and the varying response of the symmetry-breaking degrees of freedom with pressure and temperature.

## 1. Introduction

**Figure 1:**
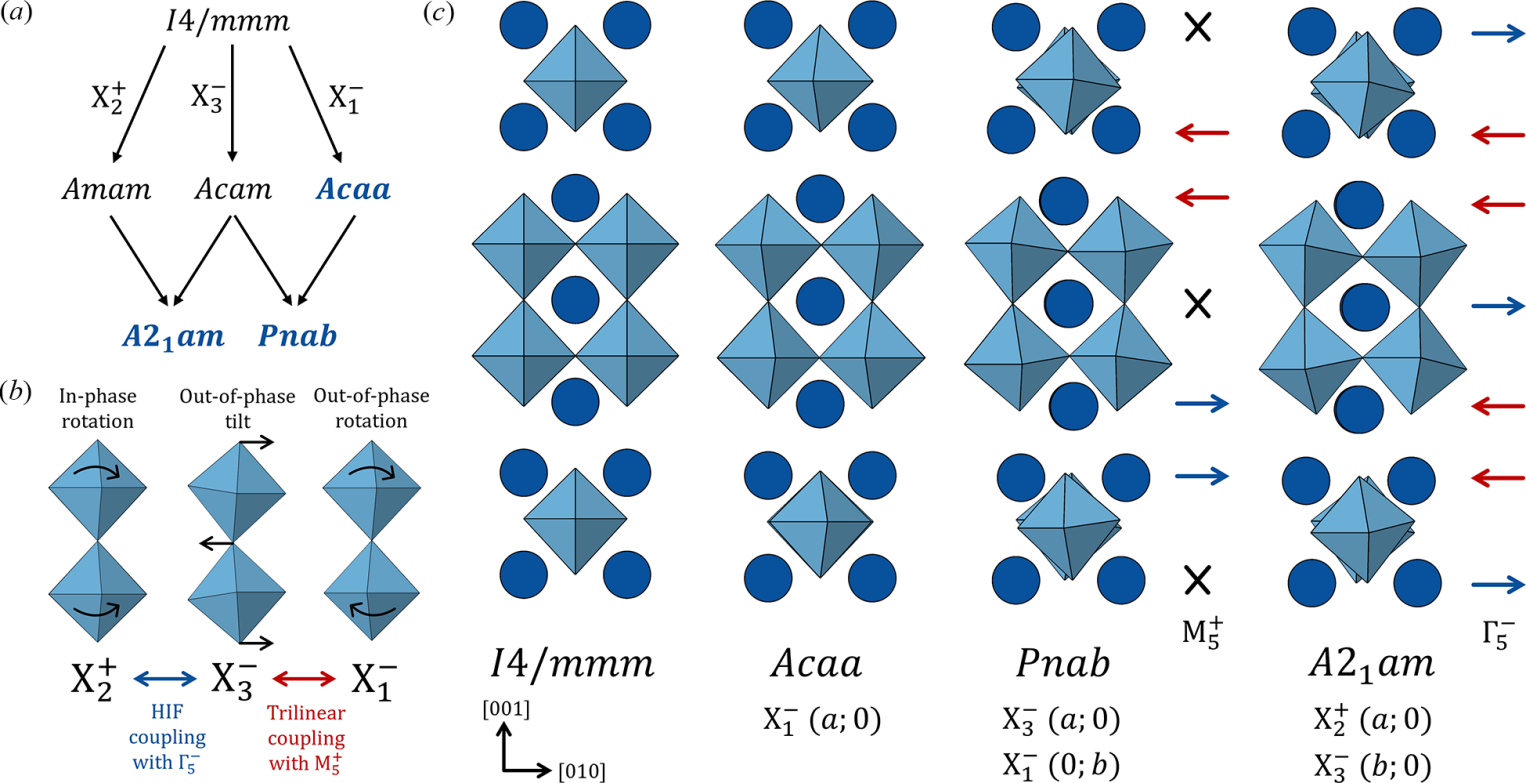
(*a*) Relationship between the phases generated from the *I*4/*mmm* aristotype through combinations of the 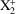, 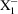 and 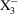 irreps. (*b*) Rotation and tilting of SnO_6_ octahedra, highlighting the hybrid improper coupling of the octahedral distortions, 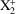 and 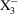 to the polar mode, Γ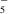 in *A*2_1_*am*, and, similarly, the trilinear coupling between 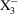, 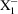 and the antiferroelectric mode, 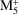 in *Pnab*. (*c*) Crystal structures of the phases of Sr_3_Sn_2_O_7_ that have been experimentally observed by temperature (Yoshida *et al.*, 2018 ▸) and pressure, showing the different order parameter directions. Sr displacements are indicated by blue and red arrows with crosses indicating no displacement. This results in an antiferroelectric distortion (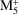) for *Pnab* and a polar distortion (Γ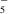) for *A*2_1_*am*.

Ferroelectric materials, which display a spontaneous electrical polarization that is reversible by an external electric field, are fundamental to many technological applications such as non-volatile memory and sensing devices (Scott, 2007 ▸). Polarization in conventional proper ferroelectrics like BaTiO_3_ and Pb(Zr/Ti)O_3_ arises through a single primary lattice distortion/instability. However, hybrid improper ferroelectrics (HIFs) have recently emerged as an intriguing alternative (Benedek & Fennie, 2011 ▸; Bousquet *et al.*, 2008 ▸; Oh *et al.*, 2015 ▸). In these materials, polarization arises indirectly as a result of a trilinear coupling mechanism between two non-polar structural distortions and a polar mode, generating an overall polar structure. This expands the range of materials that can exhibit ferroelectricity and, additionally, negates the strict chemical requirements that often restrict proper ferroelectrics from becoming multiferroic. Hybrid improper ferroelectricity is prototypical to layered perovskites, such as Ruddlesden–Popper (RP) perovskites which have a structure consisting of *n* perovskite layers intercalated with a rock-salt-like layer. Here, the two non-polar structural distortions are the tilting and rotation of the oxygen octahedra, highlighted in Fig. 1 ▸. This mechanism, where polarization is a secondary rather than primary order parameter, can provide a rich platform for manipulating polarization through the interplay of structural degrees of freedom.

External stimuli such as temperature and pressure can be utilized to directly control the structural distortions which allows for exploration of the ferroelectric mechanism with a view to how the structure may be adapted to tune the ferroelectric character of HIFs for technological use. Variable-temperature studies on *n* = 2 RP phases (RP2) (Senn *et al.*, 2015 ▸; Kratochvilova *et al.*, 2019 ▸; Yoshida *et al.*, 2018 ▸; Pomiro *et al.*, 2020 ▸) have shown that numerous phases are accessible through different combinations of octahedral distortions, where phases with a lower tolerance factor tend to have a greater degree of octahedral rotations and tilting. Whilst it is typically understood that hydrostatic pressure suppresses polarization in proper ferroelectrics (Ishidate *et al.*, 1997 ▸; Bousquet & Ghosez, 2006 ▸), in HIFs pressure can, instead, act to enhance polarization. Additionally, we have recently demonstrated by a combined experimental high-pressure powder X-ray diffraction and density functional theory (DFT) study that, for Ca_3_Ti_2_O_7_ (Clarke *et al.*, 2024 ▸), the amplitude of the octahedral rotations is increased by external pressure which, in turn, enhances the polarization by the trilinear coupling mechanism.

Sr_3_Sn_2_O_7_ is a desirable candidate for the technological realization of HIFs as it is ferroelectric at room temperature and exhibits a small polarization switching field (Xu *et al.*, 2020 ▸). The temperature-induced phase transitions of Sr_3_Sn_2_O_7_ have been previously studied by X-ray and powder neutron diffraction which revealed a complex series of phase transitions from the polar ground state *A*2_1_*am* phase to a non-polar *Pnab* phase at 410 K, with subsequent transitions to *Acaa* and *I*4/*mmm* at around 700 and 900 K, respectively (Yoshida *et al.*, 2018 ▸). Another study (Smith *et al.*, 2021 ▸), using infrared spectroscopy (IR) and interpreted with the aid of DFT, suggested that the same sequence of phase transitions is induced by increasing pressure, with these transitions occurring at approximately 2, 15 and 18 GPa. It should be noted that this sequence is unique to Sr_3_Sn_2_O_7_.

The structural differences between these phases can be visualized as distortions acting on the tetragonal aristotype *I*4/*mmm*, as shown in Fig. 1 ▸. The *A*2_1_*am* phase arises as a consequence of the hybrid improper mechanism whereby an in-phase rotation and an out-of-phase tilt of the SnO_6_ octahedra, transforming as irreducible representations 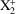 (*a*; 0) and 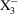 (*b*; 0), respectively, couple to a polar displacement with irrep Γ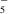. The *Pnab* phase also exhibits a trilinear coupling term which instead arises due to the combination of the out-of-phase tilt, 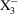 (*a*; 0), and an out-of-phase rotation, 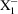 (0; *b*) with an antiferrodistortive displacement, 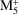. Despite the out-of-phase tilt, 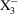, remaining in both phases, there is no pathway along which the 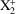 transforms directly and continuously into 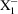. As such, this transition would be first-order, as there is no group–subgroup relationship. Finally, the *Acaa* phase only exhibits the out-of-phase rotation, 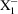 (*a*; 0).

Although the pressure-dependent response of the octahedral distortions in Sr_3_Sn_2_O_7_ has been investigated previously by first principle calculations (Ramkumar & Nowadnick, 2021 ▸), it has not been directly investigated experimentally.

In this work, we investigate the evolution of the structure of Sr_3_Sn_2_O_7_ as a function of temperature and pressure. We perform variable-temperature single-crystal X-ray diffraction between 100 and 480 K, allowing us to access a phase transition from *A*2_1_*am* to *Pnab* and confirming the previously published transition. We also undertake the first single-crystal high-pressure X-ray diffraction experiment at ambient temperature using a diamond anvil cell to achieve pressures up to 31 GPa. In contrast to the previously reported phase transitions from IR spectroscopy (Smith *et al.*, 2021 ▸), we do not find evidence for a phase transition from *A*2_1_*am* to *Pnab*. Instead, we observed a transition directly from *A*2_1_*am* to *Acaa* at around 12 GPa with no further transition to *I*4/*mmm* up to the limit of our experiment. To investigate the response of the octahedral distortions to temperature and pressure, we utilize symmetry-adapted distortion analysis. Our results show clearly that while both increasing temperature and pressure act to suppress the amplitude of the octahedral tilting, 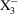, the two physical control parameters produce an opposite response with respect to the octahedral rotations, 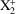 and 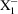, providing a likely explanation for the different phase transition pathways that are observed.

## 2. Methods

### 2.1. Sample growth

Single crystals of Sr_3_Sn_2_O_7_ were grown using a laser floating zone furnace, as described by Xu *et al.* (2020 ▸).

### 2.2. Variable-temperature XRD

A polarized light microscope was used to select a crystal that did not possess rotated orthorhombic twin domains which was confirmed by routine in-house diffraction screening. Images showing orthorhombic twin domains are included in the supporting information (Fig. S1). The single crystal of dimensions 50 × 50 × 15 µm was mounted using Fomblin-Y on a Mitigen cryoloop. Variable-temperature single-crystal X-ray diffraction measurements were collected in Experimental Hutch 1 (EH1) at Beamline I19 of the Diamond Light Source using a wavelength of λ = 0.6889 Å (corresponding to a beam energy of approximately 18.0 keV). Measurements were collected on heating from 100 K to 480 K and on cooling from 480 K to 300 K.

Indexing, integration and refinalization were performed using *CrysAlisPRO* (Rigaku, 2023 ▸). Data were reduced initially in *P*1 with no rejection conditions applied to eliminate any bias. The structure was initially solved for the 100 K collection in *A*2_1_*am* using *SHELXT* (Sheldrick, 2015*b* ▸) and subsequent temperatures by isomorphous replacement, each refined to convergence using *SHELXL* (Sheldrick, 2015*a* ▸) implemented through *Olex2* (Dolomanov *et al.*, 2009 ▸). The presence of inversion twinning in the *A*2_1_*am* phase was accounted for with the twin law, ([−1 0 0], [0 −1 0], [0 0 −1]) for which the batch scale factor (BASF) was refined to approximately 0.50.

Once a suitable model was established and the space group confirmed, data reduction was repeated with the appropriate symmetry expectations and the lattice parameters extracted.

### 2.3. High-pressure XRD

A single crystal of dimensions 50 × 30 × 15 µm was loaded into a LeToullec style four-pin membrane diamond anvil cell (DAC) equipped with Boehler-Almax anvils with 400 µm culets and a rhenium gasket which was preindented to 50 µm with a 250 µm diameter sample chamber spark eroded into the indent. Care was, again, taken to ensure that the sample did not possess orthorhombic twinning. Helium was used as a pressure transmitting medium to ensure the sample was compressed under hydrostatic conditions with a ruby sphere used as a pressure indicator, measured by fluorescence (Syassen, 2008 ▸). Single-crystal X-ray diffraction measurements were performed using a four-axis Newport diffractometer equipped with a Dectris Eiger CdTe detector, operating at a wavelength of λ = 0.4859 Å (corresponding to a beam energy of approximately 25.5 keV), in Experimental Hutch 2 (EH2) at Beamline I19 of the Diamond Light Source. Measurements were taking at intervals from ambient pressure to 30.1 (2) GPa, as detailed in the results section below.

Data reduction followed similarly as for the variable-temperature data. As is typical in high-pressure experiments, the data are incomplete on account of the body of the DAC reducing the reciprocal space available. This is exacerbated by the cell having a decreased opening angle (φ/2 = 23°) to allow higher pressures to be reached, while the *c* axis is particularly poorly defined on account of the orientation of the plate-like crystal within the DAC and a high mosaicity observed along *c*. Suitable models were identified based on the variable-temperature study and other common distorted RP2 structures (*A*2_1_*am*, *Pnab*, *Acaa*, *P*4/*nbm* and *I*4/*mmm*) and generated using *ISODISTORT*. Each model was tested by refining to convergence using *SHELXL*, implemented through *Olex2*.

The distances between apical oxygens and Sn (O–Sn–O) were restrained to be similar with an e.s.d. of 0.2 Å to maintain chemical and physical sensibility of the model. Atomic displacement parameters were refined isotropically, with oxygens restrained to be similar. These restraints were necessary to obtain self-consistent, physically sensible, fully converged refinements for the final models.

## 3. Results

### 3.1. Variable-temperature XRD

**Figure 2:**
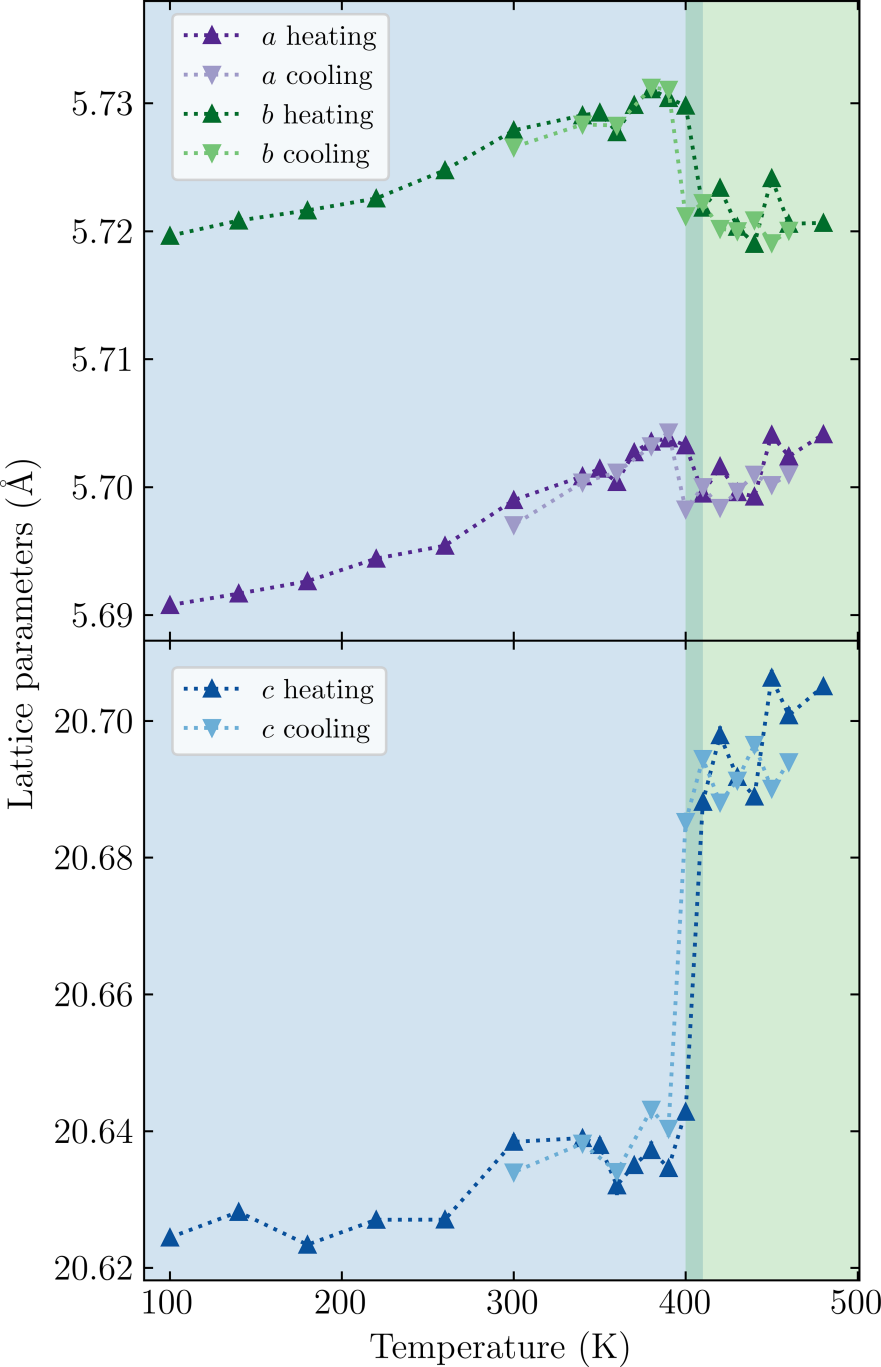
Lattice parameters as a function of temperature on heating and cooling. Blue and green backgrounds indicate the *A*2_1_*am* and *Pnab* phases, respectively, with the region between 400 and 410 K showing hysteresis.

Single-crystal X-ray diffraction data were collected on heating from 100 to 480 K and on cooling from 480 to 300 K. Within this temperature range, only the *A*2_1_*am* and *Pnab* phases were accessible. Fig. 2 ▸ shows the temperature-dependent evolution of the lattice parameters, where a significant discontinuity is clear upon heating to above 400 K. A hysteresis of 10 K is observed for this transition between heating and cooling. We, therefore, confirm a first-order structural phase transition from *A*2_1_*am* to *Pnab*, in agreement with previous work (Yoshida *et al.*, 2018 ▸).

**Figure 3:**
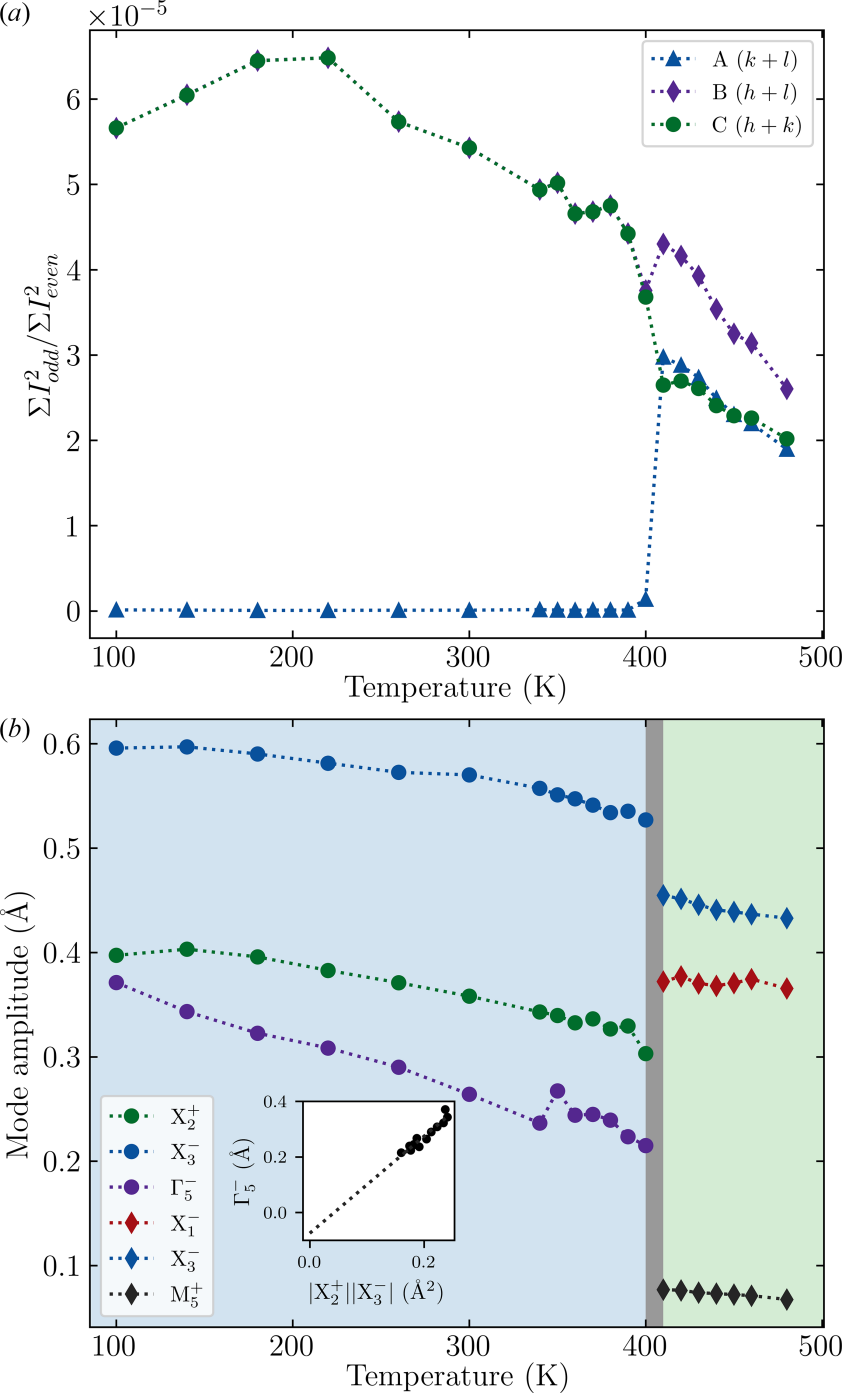
(*a*) The sum of the squares of each *hkl* reflection where *k* + *l*, *h* + *l*, *h* + *k* = odd, normalized by *k* + *l*, *h* + *l*, *h* + *k* = even, respectively. Excluding reflections where *h*, *k*, *l* = 0. (*b*) Distortion mode amplitudes as a function of temperature with inset showing hybrid improper coupling in the *A*2_1_*am* phase. Blue and green indicate the *A*2_1_*am* and *Pnab* phases, respectively.

To further investigate and confirm the high-temperature phase as *Pnab*, we can assess the adherence to certain reflection conditions. Observation of super-structure reflections, given by *k* + *l* = odd, corresponding to the violation of *A*-centering would corroborate a change in centering. Fig. 3 ▸(*a*) shows that, from 100 to 400 K, there is negligible intensity in *k* + *l* = odd reflections and so *A* centering is indeed present. However, upon heating to 410 K, there is a clear change to *P* centering as intensity is generated for this class of reflections, similar to the classes corresponding to violation of *B* and *C* centering conditions.

To explore the evolution of the atomic level structure, the structures were decomposed in terms of symmetry-adapted displacements using *ISODISTORT* (Campbell *et al.*, 2006 ▸; Stokes *et al.*, 2013 ▸), as shown in Fig. 3 ▸(*b*). The amplitude of the octahedral tilt, 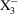, and rotation, 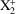, both decrease steadily as a function of temperature. For HIFs, it is a specific prediction that the amplitude of the polar distortion should be linearly proportional to the product of the amplitude of the driving order parameters (*Q*) (Benedek & Fennie, 2011 ▸). To test the validity of the assumption that Sr_3_Sn_2_O_7_ is indeed strictly a HIF, we hence plot *Q*(Γ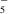) against *Q*(X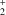)*Q*(X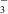), shown in Fig. 3 ▸(*b*). A strong linear trend with an intercept close to zero is shown [*y* = 1.83 (14)*x* − 0.10 (3)], demonstrating that the polarization is driven directly by the octahedral rotations and tilts and that there is no appreciable proper contribution to the ferroelectric polarization. This is at odds to what has been observed for Ca_3_Ti_2_O_7_, where an appreciable proper contribution to the polarization is expected based on DFT calculations (Clarke *et al.*, 2024 ▸).

Above the phase transition temperature, the improper coupling to the polar distortions is necessarily turned off and replaced by a coupling to the antipolar displacements, 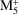. This coupling is shown in the supporting information (Fig. S2). The amplitudes of both 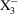 and 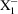 decrease steadily with increasing temperature, as to be expected as we approach the vicinity of the entropically stabilized undistorted *I*4/*mmm* aristotype.

### 3.2. High-pressure XRD

**Figure 4:**
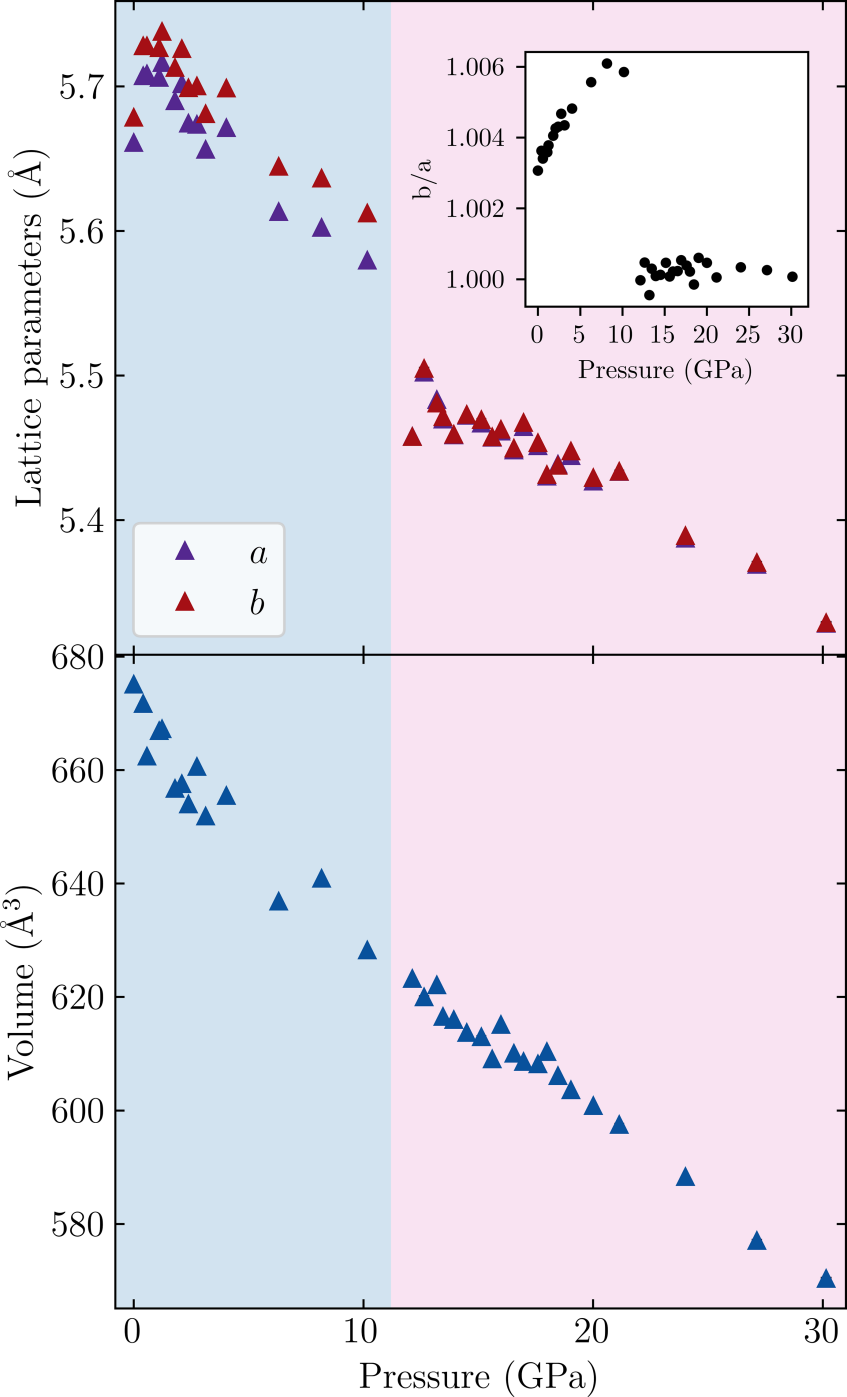
Pressure dependence of the *a* and *b* lattice parameters and volume. Inset shows *b*/*a* lattice parameter ratio. Blue and pink backgrounds indicate *A*2_1_*am* and *Acaa* phases, respectively. The first data point at 0 GPa was collected in cell but prior to gas-loading with He. Pressure error bars are smaller than size of markers.

We collected high-pressure single-crystal X-ray diffraction data between ambient pressure and 30.1 (2) GPa. Fig. 4 ▸ shows how the *a* and *b* lattice parameters and volume evolve throughout this pressure range. In contrast to previous work using IR spectroscopy (Smith *et al.*, 2021 ▸), there does not appear to be any evidence of a phase transition from *A*2_1_*am* to *Pnab* at around 2 GPa that would manifest itself in a discontinuity in the lattice parameters. However, there is a large discontinuity at approximately 11 GPa.

To identify these phases, models were tested in *A*2_1_*am*, *Pnab*, *P*4/*nbm* and *Acaa* (and *I*4/*mmm* for higher pressures). Between 0 and 10.17 (18) GPa, a structural model refined in *A*2_1_*am* provides the best fit. The *Pnab* and *P*4_2_/*nbm* models tested provided significantly worse fitting statistics or would not converge without additional constraints. Further details are provided in Tables S4–S6.

From 10.17 (18) to 30.1 (2) GPa, *Acaa* provides the most reasonable fit. The inset of Fig. 4 ▸ shows that there is a significant decrease in the degree of orthorhombicity as the *b*/*a* ratio decreases to values close to one in this pressure range. This has been demonstrated previously for the *Acaa* phase in the variable-temperature diffraction study (Yoshida *et al.*, 2018 ▸).

**Figure 5:**
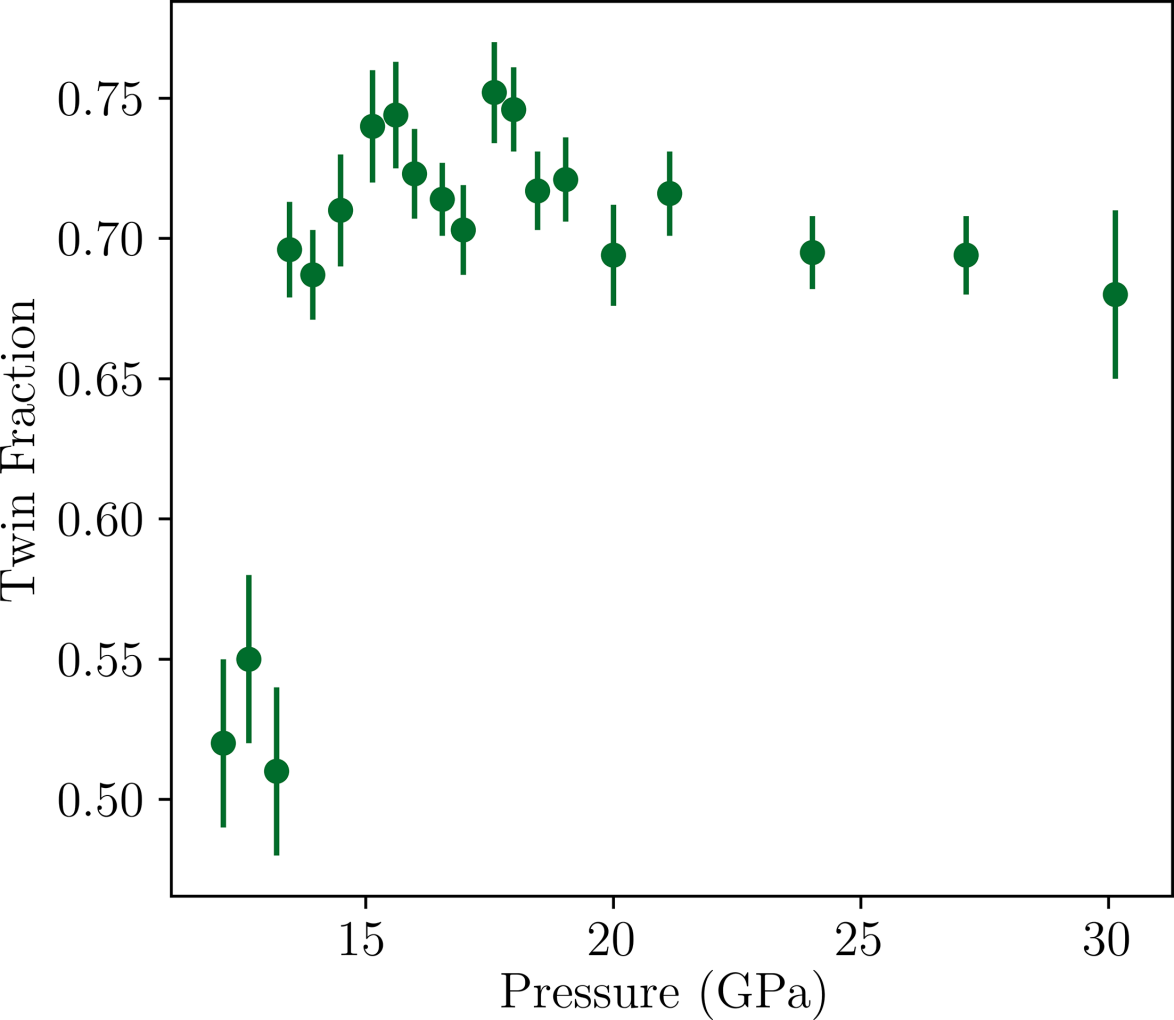
Pressure-dependent evolution of BASF within the *Acaa* phase. Pressure error bars are smaller than size of markers.

In the region between 12.13 (14) and 13.20 (18) GPa, both the *A*2_1_*am* and *Acaa* models converge successfully, which could indicate a region of phase coexistence. Interestingly, despite the fact that orthorhombic twinning is not present in the *A*2_1_*am* phase, it appears in the *Acaa* phase. This provides further evidence that the phase transition is not continuous in either of the order parameters. As shown in Fig. 5 ▸, between 12.13 (14) and 13.20 (18) GPa, the twin fraction {refined via the BASF in *SHELXL* with the twin law ([0 1 0], [−1 0 0], [0 0 1])} refines to approximately 0.5, consistent with the same region where both *A*2_1_*am* and *Acaa* models converge successfully. This twin fraction then increases to around 0.7 beyond this, corresponding to 30% of the *A*-centered domain [X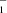 (*a*; 0)] and 70% of the *B*-centered domain [X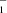 (0; *a*)]. This may occur to relieve strain in the *Acaa* phase but it may, instead, indicate that it is easier to nucleate the 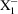 along (0; *a*) compared to (*a*; 0) to alleviate competition with the rotation (X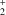) in *A*2_1_*am*.

**Figure 6:**
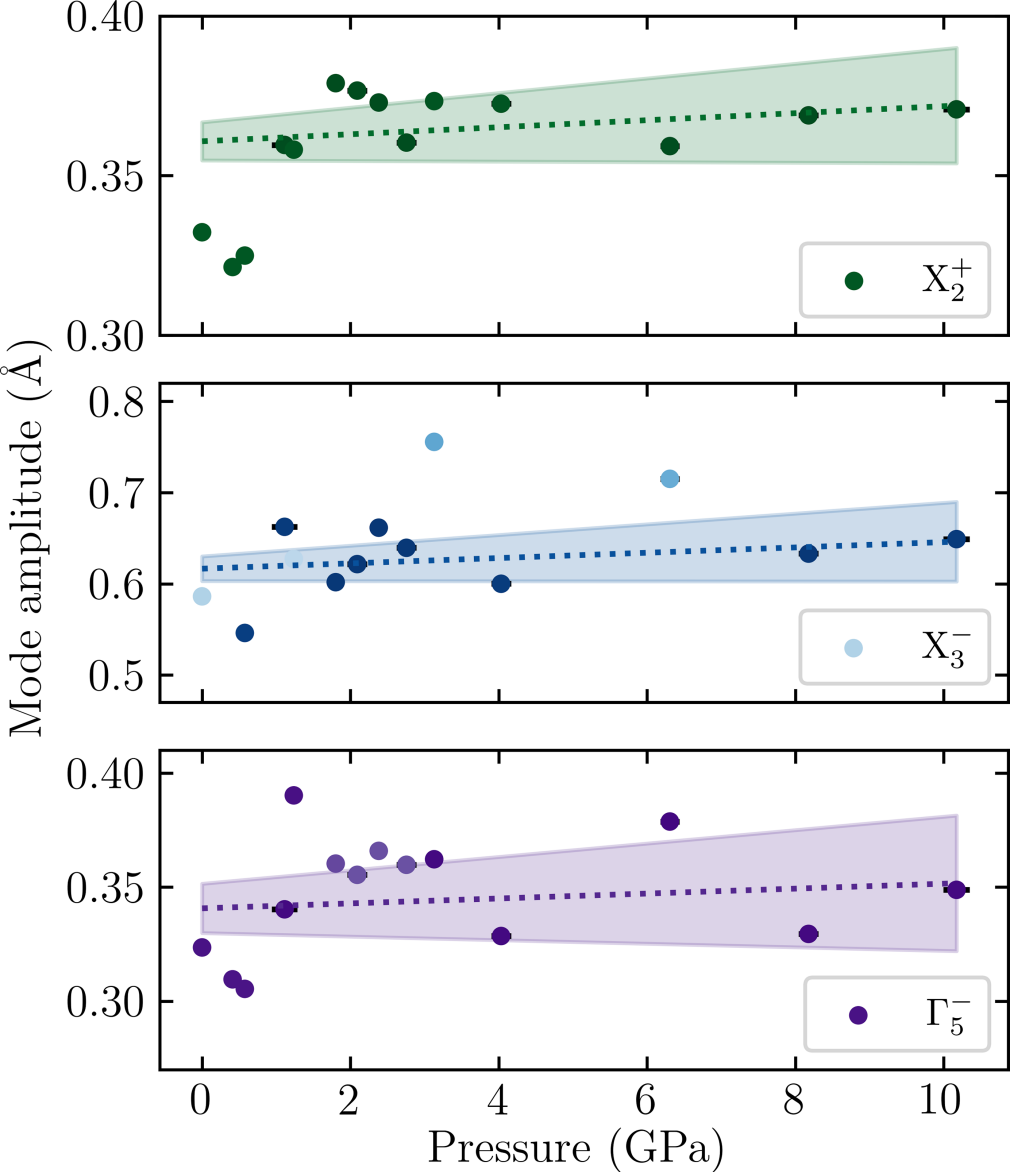
Distortion mode amplitudes as a function of pressure for *A*2_1_*am* phase, showing the rotation, 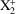, and tilt, 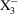, and polar, Γ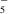, modes. Darker and lighter markers indicate a lower and higher uncertainty, respectively. A weighted linear regression was performed based on errors generated through *AMPLIMODES* with shaded regions showing the confidence interval of one sigma.

To further investigate the evolution of the atomic structure under pressure, the ferroelectric phase was decomposed in terms of symmetric-adapted displacements, as shown in Fig. 6 ▸, using *ISODISTORT* and *AMPLIMODES* (Orobengoa *et al.*, 2009 ▸; Perez-Mato *et al.*, 2010 ▸). The noise and outliers in these data, which we attribute to the inherent complications of the high-pressure experiment, particularly the limited data completeness, illustrate the difficulty in refining the subtle structural distortions associated with the octahedral rotation and tilting that are dominated by oxygen displacements. However, the results still provide a useful upper and lower bound for what should be expected for the pressure-dependent evolution of the octahedral rotation and tilt modes. As the pressure increases, the amplitude of all modes remains relatively constant, exhibiting a shallow gradient with a confidence interval that encompasses both negative and positive values. This is broadly in line with computational predictions by Ramkumar & Nowadnick (2021 ▸) which shows a slight decrease in all distortions modes within the same 0 to 10 GPa pressure range. Regardless of the overall trends, the persistence of the polar mode, Γ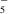, indicates that the ferroelectric, *A*2_1_*am*, is stable to much higher pressures than was previously inferred from the IR study.

**Figure 7:**
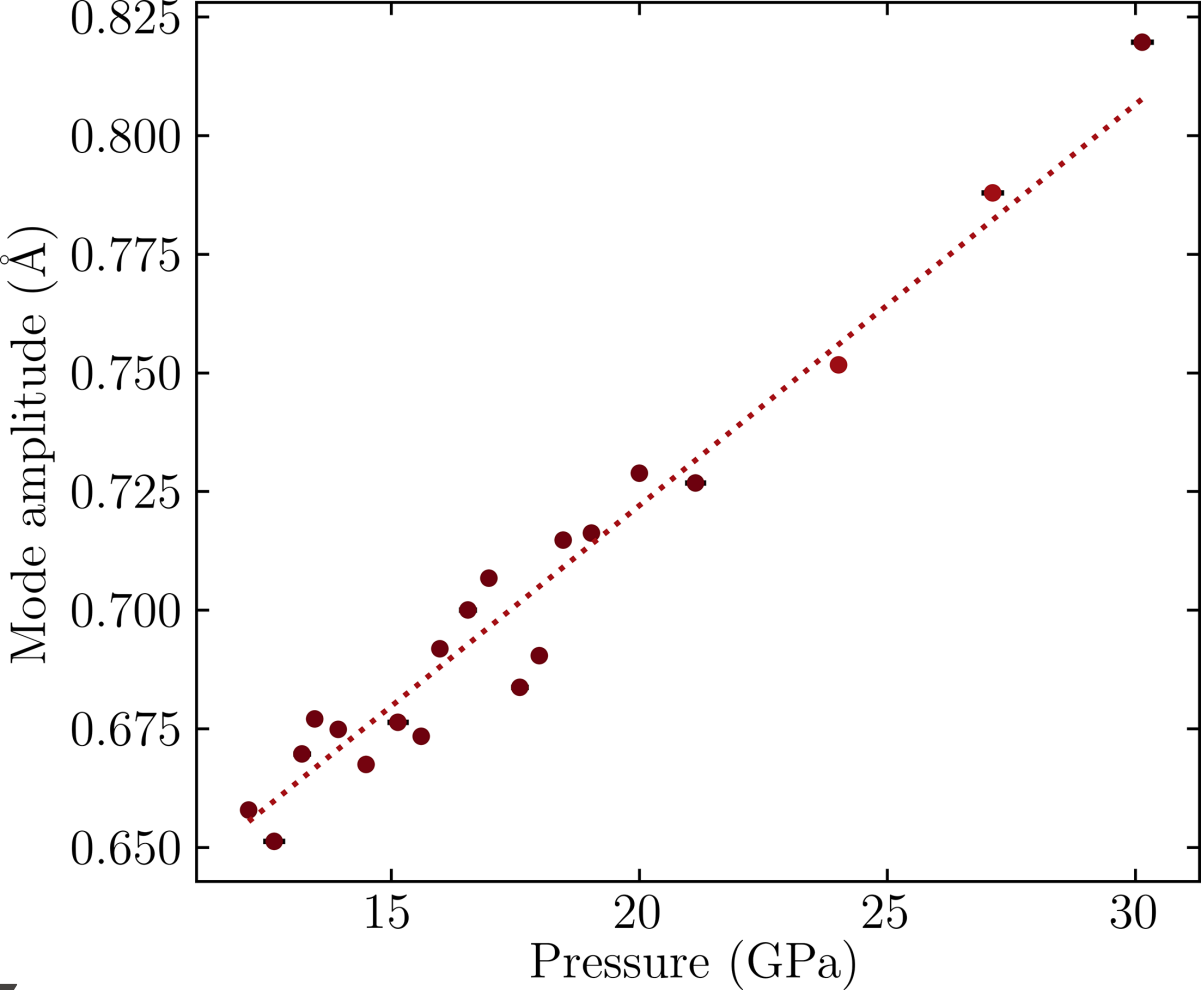
Evolution of the out-of-phase rotation, 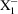, as a function of pressure for *Acaa* phase. A weighted linear regression was performed based on errors generated from *AMPLIMODES*.

Fig. 7 ▸ shows how the out-of-phase rotation, 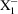, of the *Acaa* phase evolves above 10 GPa. The amplitude increases steadily and linearly with pressure, suggesting that the *Acaa* phase is continuing to be stabilized relative to the aristotype symmetry. Accordingly, we do not necessarily expect a further phase transition to the aristotype symmetry *I*4/*mmm*. From refinements, no transition from *Acaa* to *I*4/*mmm* was identified, even to the limit of our experiment at 30.1 (2) GPa.

Compressibility parameters were calculated using *PASCal* (Cliffe & Goodwin, 2012 ▸) also provide evidence to this conclusion. These data are tabulated in the supporting information (Table S7). For both the *A*2_1_*am* and *Acaa* phases, the *c* axis is more compressible than the *a* and *b* axes and, additionally, the *c* axis is more compressible in *Acaa* than in *A*2_1_*am*. Together, this failure to recover the *I*4/*mmm* phase at high pressure is consistent with the observed uniaxial negative thermal expansion in isosymmetric RP2, Ca_3_Mn_2_O_7_, which we have found is related to the unusually high anisotropic compressibility mediated by the octahedral rotations in these phases (Senn *et al.*, 2016 ▸; Ablitt *et al.*, 2017 ▸; Ablitt *et al.*, 2018 ▸).

We find that our results do not follow the trend that the application of pressure induces the same sequence of phase transitions as with increasing temperature. The effect of pressure and temperature on the structural distortions in the ferroelectric, *A*2_1_*am*, phase also differs slightly from each other. Whilst increasing temperature causes a distinct decrease in the tilting and rotation modes and, therefore, a decrease in the polar mode, the pressure-dependent trends are much more marginal. Compared to Ca_3_Ti_2_O_7_, where the amplitude of the rotation increases faster than the amplitude of the tilt decreases (Clarke *et al.*, 2024 ▸), this does not appear to be the case for Sr_3_Sn_2_O_7_.

Finally, we note that while our results appear to contradict the IR results (Smith *et al.*, 2021 ▸), in the same study the authors report ground state DFT energies which appear to show that *Acaa*, not *Pnab*, is the stable high-pressure phase. The differences in the experimental phase transitions reported could be as a result of the pressure transmitting medium used. Although the exact hydrostatic limit of petroleum jelly, as used in the IR study, has not been previously reported, we speculate that it is much lower than 4 GPa, in line with the limits of similar media (Klotz *et al.*, 2009 ▸). As the differences between the structures are subtle, they are likely very sensitive to non-hydrostatic conditions. Understanding the discrepancy between results derived from crystallographic studies and those of a spectroscopic nature would require substantial further investigation.

## Supplementary Material

CIFs for the varying temperature and pressure studies of SrSn2O7. DOI: 10.1107/S2052520625002306/yh5041sup1.zip

Tables S1-S6 and Fig. S1. DOI: 10.1107/S2052520625002306/yh5041sup2.pdf

CCDC references: 2434683, 2434684, 2434685, 2434686, 2434687, 2434688, 2434689, 2434690, 2434691, 2434692, 2434693, 2434694, 2434695, 2434696, 2434697, 2434698, 2434699, 2434700, 2434701, 2434702, 2434703, 2434704, 2434705, 2434706, 2434707, 2434708, 2434709, 2434710, 2434711, 2434712, 2434713, 2434714, 2434715, 2434716, 2434717, 2434718, 2434719, 2434720, 2434721, 2434722, 2434723, 2434724, 2434725, 2434726, 2434727, 2434728, 2434729, 2434730, 2434731, 2434732, 2434733, 2434734, 2434735, 2434736, 2434737, 2434738, 2434739, 2434740, 2434741, 2434742, 2434743, 2434744, 2434745, 2434746, 2434747, 2434748

## Data Availability

All CIFs underpinning the results presented here are available as part of the supporting information.

## References

[bb1] Ablitt, C., Craddock, S., Senn, M. S., Mostofi, A. A. & Bristowe, N. C. (2017). *npj Comput. Mater.* **3**, 44.

[bb2] Ablitt, C., Mostofi, A. A., Bristowe, N. C. & Senn, M. S. (2018). *Front. Chem.* **6**, 455. 10.3389/fchem.2018.00455 PMC6201142 30406076

[bb3] Benedek, N. A. & Fennie, C. J. (2011). *Phys. Rev. Lett.* **106**, 107204. 10.1103/PhysRevLett.106.107204 21469829

[bb4] Bousquet, E., Dawber, M., Stucki, N., Lichtensteiger, C., Hermet, P., Gariglio, S., Triscone, J.-M. & Ghosez, P. (2008). *Nature*, **452**, 732–736. 10.1038/nature06817 18401406

[bb5] Bousquet, E. & Ghosez, P. (2006). *Phys. Rev. B*, **74**, 180101.

[bb6] Campbell, B. J., Stokes, H. T., Tanner, D. E. & Hatch, D. M. (2006). *J. Appl. Cryst.* **39**, 607–614.

[bb7] Clarke, G., Daisenberger, D., Luo, X., Cheong, S. W., Bristowe, N. C. & Senn, M. S. (2024). *Phys. Rev. B*, **109**, 094107.

[bb8] Cliffe, M. J. & Goodwin, A. L. (2012). *J. Appl. Cryst.* **45**, 1321–1329.

[bb9] Dolomanov, O. V., Bourhis, L. J., Gildea, R. J., Howard, J. A. K. & Puschmann, H. (2009). *J. Appl. Cryst.* **42**, 339–341.

[bb10] Ishidate, T., Abe, S., Takahashi, H. & Môri, N. (1997). *Phys. Rev. Lett.* **78**, 2397–2400.

[bb11] Klotz, S., Chervin, J.-C., Munsch, P. & Le Marchand, G. (2009). *J. Phys. D Appl. Phys.* **42**, 075413.

[bb12] Kratochvilova, M., Huang, F.-T., Diaz, M. F., Klicpera, M., Day, S. J., Thompson, S. P., Oh, Y.-S., Gao, B., Cheong, S.-W. & Park, J.-G. (2019). *J. Appl. Phys.* **125**, 244102.

[bb13] Oh, Y. S., Luo, X., Huang, F.-T., Wang, Y. & Cheong, S.-W. (2015). *Nat. Mater.* **14**, 407–413. 10.1038/nmat4168 25581628

[bb14] Orobengoa, D., Capillas, C., Aroyo, M. I. & Perez-Mato, J. M. (2009). *J. Appl. Cryst.* **42**, 820–833.

[bb15] Perez-Mato, J. M., Orobengoa, D. & Aroyo, M. I. (2010). *Acta Cryst.* A**66**, 558–590. 10.1107/S0108767310016247 20720321

[bb16] Pomiro, F., Ablitt, C., Bristowe, N. C., Mostofi, A. A., Won, C., Cheong, S.-W. & Senn, M. S. (2020). *Phys. Rev. B*, **102**, 014101.

[bb17] Ramkumar, S. P. & Nowadnick, E. A. (2021). *Phys. Rev. B*, **104**, 144105.

[bb18] Rigaku (2023). *CrysAlisPro*. Rigaku Oxford Diffraction, Yarnton, England.

[bb19] Scott, J. F. (2007). *Science*, **315**, 954–959. 10.1126/science.1129564 17303745

[bb20] Senn, M., Bombardi, A., Murray, C., Vecchini, C., Scherillo, A., Luo, X. & Cheong, S. (2015). *Phys. Rev. Lett.* **114**, 035701. 10.1103/PhysRevLett.114.035701 25659007

[bb21] Senn, M. S., Murray, C. A., Luo, X., Wang, L., Huang, F.-T., Cheong, S.-W., Bombardi, A., Ablitt, C., Mostofi, A. A. & Bristowe, N. C. (2016). *J. Am. Chem. Soc.* **138**, 5479–5482. 10.1021/jacs.5b13192 26927232

[bb22] Sheldrick, G. M. (2015*a*). *Acta Cryst.* C**71**, 3–8.

[bb23] Sheldrick, G. M. (2015*b*). *Acta Cryst.* A**71**, 3–8.

[bb24] Smith, K. A., Ramkumar, S. P., Harms, N. C., Clune, A. J., Xu, X., Cheong, S.-W., Liu, Z., Nowadnick, E. A. & Musfeldt, J. L. (2021). *Phys. Rev. B*, **104**, 064106.

[bb25] Stokes, H. T., Hatch, D. M. & Campbell, B. J. (2013). *ISODISTORT*, *ISOTROPY* Software Suite. https://iso.byu.edu.

[bb26] Syassen, K. (2008). *High Pressure Res.* **28**, 75–126.

[bb27] Xu, X., Wang, Y., Huang, F.-T., Du, K., Nowadnick, E. A. & Cheong, S.-W. (2020). *Adv. Funct. Mater.* **30**, 2003623.

[bb28] Yoshida, S., Akamatsu, H., Tsuji, R., Hernandez, O., Padmanabhan, H., Sen Gupta, A., Gibbs, A. S., Mibu, K., Murai, S., Rondinelli, J. M., Gopalan, V., Tanaka, K. & Fujita, K. (2018). *J. Am. Chem. Soc.* **140**, 15690–15700. 10.1021/jacs.8b07998 30347981

