# Supplementary material for: The varying temperature- and pressure-induced phase transition pathways in hybrid improper ferroelectric Sr3Sn2O7
Source: Acta Crystallogr B Struct Sci Cryst Eng Mater. 2025 Apr 14;81(Pt 3):318–24. doi: 10.1107/S2052520625002306 (PMC12147935; doi:10.1107/S2052520625002306)
Supplement: Supplementary file 2 [file b-81-00318-sup2.pdf]

STRUCTURAL SCIENCE
CRYSTAL ENGINEERING
MATERIALS

Volume 81 (2025)

Supporting information for article:

The varying temperature- and pressure-induced phase transition pathways in hybrid improper ferroelectric Sr3Sn2O7

Evie Ladbrook, Jeremiah P. Tidey, Fei-Ting Huang, Sang-Wook Cheong, Dominik Daisenberger, Mark R. Warren and Mark S. Senn

Supporting information for 'The varying temperature- and pressure-induced phase transition pathways in hybrid improper ferroelectric $Sr_3Sn_2O_7$

Evie Ladbrook[a], Jeremiah P. Tidey[b], Fei-Ting Huang[c], Sang-Wook Cheong[c], Dominik Daisenberger[d], Mark R. Warren[d], and Mark S. Senn[a]

a) Department of Chemistry, University of Warwick, Gibbet Hill, Coventry, CV4 7AL, United Kingdom
b) Department of Physics, University of Warwick, Gibbet Hill, Coventry, CV4 7AL, United Kingdom
c) Keck Center for Quantum Magnetism, Rutgers University, Piscataway, New Jersey, 07928, USA
d) Diamond Light Source Ltd, Harwell Science and Innovation Campus, Didcot OX11 0DE, United Kingdom

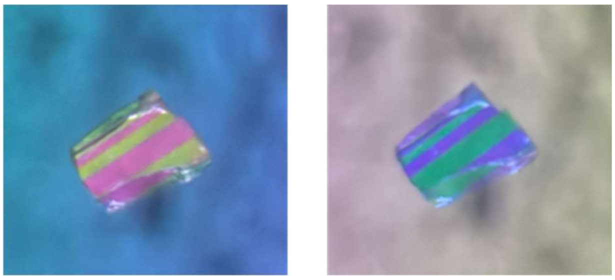

Figure S1. Orthorhombic twin domains in $Sr_3Sn_2O_7$ visualised by polarised light microscopy.

Table S1: Variable temperature lattice parameters.

Temperature (K)	*a* (Å)	*b* (Å)	*c* (Å)
100	5.69077(13)	5.71964(14)	20.6244(5)
140	5.69167(14)	5.72083(14)	20.6282(5)
180	5.6926(2)	5.7216(2)	20.6234(9)
220	5.6944(2)	5.7226(2)	20.6270(9)
260	5.6954(2)	5.7248(3)	20.6271(10)
300	5.6990(3)	5.7279(3)	20.6384(11)
340	5.7008(2)	5.72906(19)	20.6390(7)
350	5.7014(3)	5.7293(3)	20.6380(11)
360	5.7004(3)	5.7277(4)	20.6321(14)
370	5.7027(3)	5.7299(3)	20.6350(11)
380	5.7036(3)	5.7311(3)	20.6372(13)
390	5.7038(3)	5.7304(3)	20.6346(9)
400	5.7033(2)	5.7298(3)	20.6428(9)

410	5.6995(3)	5.7218(3)	20.6881(10)
420	5.7016(3)	5.7234(4)	20.6979(13)
430	5.6996(2)	5.7203(2)	20.6918(8)
440	5.69925(15)	5.71901(17)	20.6889(5)
450	5.7041(3)	5.72414(3)	20.7063(12)
460	5.7024(3)	5.72058(3)	20.7008(12)
480	5.70412(15)	5.72065(17)	20.7051(5)
460	5.70100(17)	5.7201(2)	20.6940(8)
450	5.70017(15)	5.71908(19)	20.6901(7)
440	5.7010(3)	5.7209(3)	20.6965(11)
430	5.6997(3)	5.7200(3)	20.6913(10)
420	5.6984(2)	5.7202(3)	20.6881(9)
410	5.7000(3)	5.7222(3)	20.6945(13)
400	5.6983(3)	5.7212(3)	20.6852(14)
390	5.7043(3)	5.7311(3)	20.6404(12)
380	5.7032(3)	5.7312(3)	20.6432(12)
360	5.7011(3)	5.7283(3)	20.6341(10)
340	5.70038(12)	5.72834(13)	20.6382(5)
300	5.69703(19)	5.7266(2)	20.6340(7)

Table S2: R_1, wR_2, R_{int} and goodness-of-fit (GooF) for variable temperature study. Results presented here are self-consistent but stem from refinements carried out using SHELXL 2014/7 implemented through Olex2 1.5 (2021.12.09). Grey areas indicate where the refinement could not converge.

	A2₁am				*Pnab*			
Temperature (K)	R_1 (%)	wR_2 (%)	R_{int} (%)	GooF	R_1 (%)	wR_2 (%)	R_{int} (%)	GooF
100	7.32	18.33	5.31	1.122				
140	7.11	17.47	6.12	1.150				
180	7.09	17.48	6.42	1.154				
220	7.12	18.21	7.42	1.134				
260	7.50	19.05	7.58	1.117				
300	7.34	18.82	7.24	1.100				
340	7.47	20.42	7.75	1.060				
350	7.31	19.2	7.94	1.099				
360	7.57	19.91	7.39	1.101				
370	7.41	19.24	7.58	1.074				
380	7.40	19.03	7.59	1.110				
390	7.45	19.06	7.29	1.103				
400	7.35	18.77	6.67	1.067	12.44	47.25	6.81	1.906
410	7.97	22.3	7.20	1.049	8.47	22.89	7.36	1.026
420	7.92	23.5	7.00	1.079	8.10	22.29	7.21	1.028
430	9.21	23.13	6.59	1.234	8.23	22.59	6.78	1.010

440					8.29	23.06	7.35	1.011
450					8.01	22.55	6.64	1.010
460					8.31	23.01	7.18	1.024
480					8.42	23.94	7.44	1.045
460					8.66	24.01	8.82	1.014
450					8.33	23.74	7.51	1.011
440					8.17	22.78	7.81	1.028
430					8.33	24.03	7.86	1.010
420	8.97	29.54	6.98	1.242	8.30	23.09	7.18	1.032
410	7.89	22.99	6.95	1.033	8.32	22.66	7.13	1.025
400	7.61	20.82	6.73	1.021	8.25	23.11	6.86	1.038
390	7.27	19.57	7.36	1.054				
380	7.23	19.40	7.23	1.071				
360	7.15	20.34	7.90	1.066				
340	7.31	19.80	7.23	1.067				
300	7.13	19.92	7.59	1.047				

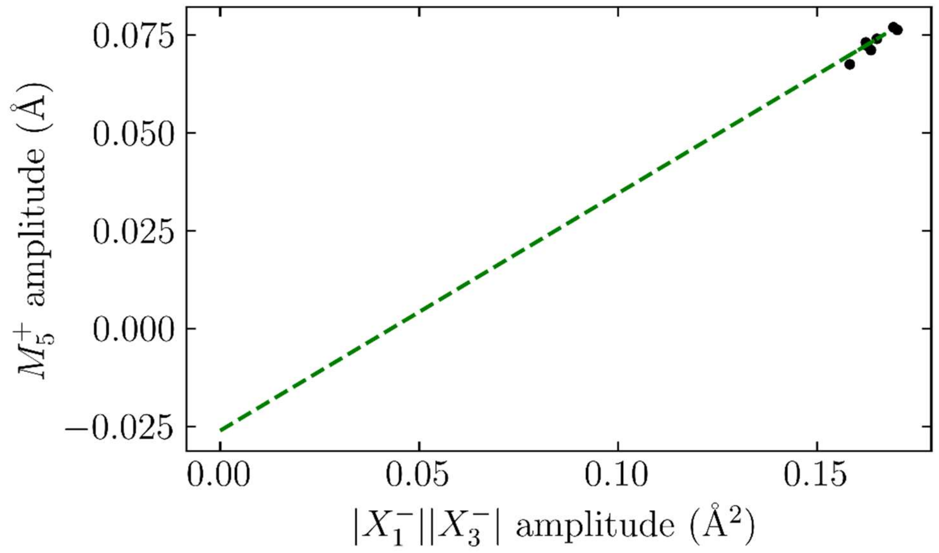

Figure S2. Trilinear coupling between rotation modes, X_1^- and X_3^-, and antiferroelectric mode, M_5^+, in the *Pnab* phase giving, y = 0.61(12)x – 0.026(19)

Table S3: High pressure lattice parameters.

Pressure (GPa)	*a* (Å)	*b* (Å)	*c* (Å)
0.00(5)	5.6611(6)	5.6784(17)	21.00(19)
0.41(5)	5.7072(6)	5.7279(13)	20.55(12)
0.58(5)	5.7085(6)	5.7279(6)	20.26(11)
1.11(17)	5.7061(7)	5.7266(7)	20.41(11)
1.24(5)	5.7163(6)	5.7379(6)	20.34(11)
1.80(9)	5.6899(7)	5.7129(7)	20.20(10)
2.09(13)	5.7016(7)	5.7259(7)	20.14(11)

2.38(9)	5.6744(7)	5.6988(7)	20.22(11)
2.76(13)	5.6737(6)	5.7002(6)	20.43(10)
3.13(9)	5.6563(8)	5.6809(8)	20.29(13)
4.03(13)	5.6713(7)	5.6986(6)	20.28(10)
6.31(13)	5.6133(8)	5.6446(8)	20.10(15)
8.18(13)	5.6023(8)	5.6364(7)	20.30(14)
10.17(18)	5.5796(17)	5.6123(16)	20.1(3)
12.13(14)	5.4579(7)	5.4577(8)	20.92(19)
12.6(2)	5.5022(9)	5.5048(10)	20.47(18)
13.20(18)	5.4833(8)	5.4809(8)	20.70(15)
13.46(9)	5.4697(8)	5.4713(8)	20.60(15)
13.93(14)	5.4588(9)	5.4593(8)	20.67(14)
14.49(9)	5.4724(7)	5.4730(7)	20.49(13)
15.1(2)	5.4669(9)	5.4694(8)	20.50(16)
15.60(17)	5.4571(8)	5.4575(7)	20.45(14)
15.98(14)	5.4614(7)	5.4626(6)	20.62(12)
16.55(18)	5.4483(7)	5.4496(6)	20.55(12)
16.97(8)	5.4645(8)	5.4674(8)	20.37(14)
17.59(18)	5.4513(8)	5.4534(7)	20.46(13)
17.99(14)	5.4302(7)	5.4314(6)	20.69(11)
18.47(9)	5.4318(11)	5.4378(10)	20.68(19)
19.04(14)	5.4445(6)	5.4477(6)	20.35(11)
20.00(14)	5.4268(9)	5.4318(10)	20.39(15)
21.1(2)	5.4335(7)	5.4337(6)	20.24(11)
24.02(5)	5.3873(6)	5.3891(6)	20.26(9)
27.1(2)	5.3692(7)	5.3705(6)	20.01(10)
30.1(2)	5.3285(10)	5.3289(9)	20.09(15)

Table S4: R_1 (%) values for high pressure study. Data presented here are self-consistent but stem from refinements carried out using SHELXL 2014/7 implemented through Olex2 1.5 (2021.12.09). Grey areas indicate where the refinement could not converge.

Pressure (GPa)	*Pnab*	*P4/nbm*	$A2_1am$	*Acaa*
0.00(5)	8.04	8.29	2.35	
0.41(5)	8.81	8.88	4.95	
0.58(5)	10.30	12.07	6.59	
1.11(17)	11.42	13.79	4.63	
1.24(5)	15.44	13.14	5.44	
1.80(9)	13.60	11.30	4.59	
2.09(13)	13.82	12.30	4.94	
2.38(9)	14.93	12.67	4.33	
2.76(13)	10.82	10.77	5.06	
3.13(9)	12.37	10.55	7.68	
4.03(13)	12.42	9.01	5.41	
6.31(13)	11.71	13.03	6.58	

8.18(13)	9.21	8.07	5.54	
10.17(18)	9.05	8.53	4.03	
12.13(14)	5.36	13.06	5.53	2.06
12.6(2)	8.23	8.20	8.75	5.70
13.20(18)	14.78	8.16	7.44	4.44
13.46(9)	11.37	13.62		4.25
13.93(14)	9.79	10.02		4.85
14.49(9)	12.99	8.63		7.62
15.1(2)	11.72	9.37		7.48
15.60(17)	6.22	8.47		6.91
15.98(14)	5.96	8.55		4.97
16.55(18)	7.39	8.52		3.94
16.97(8)	7.23	9.17		4.34
17.59(18)	12.62	8.99		8.52
17.99(14)	14.93	12.66		5.49
18.47(9)	6.71	9.51		3.23
19.04(14)	7.50	9.87		3.74
20.00(14)	7.34	9.29		3.63
21.1(2)	9.92	8.43		4.27
24.02(5)	9.06	9.74		4.24
27.1(2)	12.09	10.32		4.32
30.1(2)	15.70	9.29		9.27

Table S5: R_1, wR_2, R_{int} and goodness-of-fit (GooF) for $A2_1am$ phase from high pressure study. Data here is presented in the main manuscript and stem from refinements using SHELXL 2019/3 implemented through Olex2 1.5-ac7-013.

Pressure (GPa)	R_1 (%)	wR_2 (%)	R_{int} (%)	GooF
0.00(5)	3.62	10.08	0.37	1.263
0.41(5)	3.24	9.65	0.23	1.332
0.58(5)	5.19	14.73	2.48	1.118
1.11(17)	2.61	9.77	0.39	1.246
1.24(5)	5.38	19.61	0.21	1.279
1.80(9)	3.85	10.18	0.37	1.128
2.09(13)	4.40	10.85	0.31	1.161
2.38(9)	3.59	9.71	0.35	1.151
2.76(13)	4.13	10.17	0.39	1.156
3.13(9)	7.65	14.69	1.94	1.222
4.03(13)	4.74	12.15	0.57	1.172
6.31(13)	5.59	12.59	1.30	1.213
8.18(13)	3.70	10.03	0.37	1.224
10.17(18)	3.62	10.08	0.37	1.293

Table S6: R_1, wR_2, R_{int} and goodness-of-fit (GooF) for *Acaa* phase from high pressure study. Data here is presented in the main manuscript and stem from refinements using SHELXL 2019/3 implemented through Olex2 1.5-ac7-013.

Pressure (GPa)	R_1 (%)	wR_2 (%)	R_{int} (%)	GooF
12.13(14)	2.47	8.50	1.69	1.162
12.6(2)	3.99	12.13	0.51	1.146
13.20(18)	3.40	10.25	0.54	1.187
13.46(9)	3.38	8.80	1.69	1.242
13.93(14)	2.78	9.45	0.67	1.181
14.49(9)	5.52	14.62	0.45	1.193
15.1(2)	4.27	16.07	1.75	1.321
15.60(17)	4.91	16.28	1.42	1.254
15.98(14)	3.67	12.02	1.20	1.277
16.55(18)	2.53	8.32	1.01	1.299
16.97(8)	3.13	11.23	1.34	1.409
17.59(18)	5.62	15.50	2.31	1.274
17.99(14)	3.52	11.60	1.41	1.149
18.47(9)	2.47	8.09	0.66	1.352
19.04(14)	2.74	9.49	0.44	1.322
20.00(14)	2.74	9.64	0.86	1.358
21.1(2)	1.68	6.20	2.63	1.277
24.02(5)	2.21	6.78	0.48	1.201
27.1(2)	3.14	8.85	1.33	1.299
30.1(2)	5.42	22.85	3.43	1.282

Table S7: High pressure compressibility parameters calculated using PASCal.

		K (TPa^{-1})	σK(TPa^{-1})	ε_0	λ	P_c	ν
$A2_1am$	*a*	1.8315	0.8507	0.0071	-0.0011	0	1.3055
	b	1.4801	0.8555	0.0075	-0.0008	0	1.4055
	c	2.0846	0.8168	19.4245	-19.456	-0.0007	0.0002
	V	6.0992	0.5428				
Acaa	*a*	1.4293	0.2966	0.0041	-0.0007	12.1256	1.3167
	b	1.3937	0.2957	0.0041	-0.0006	12.1256	1.3478
	c	3.0646	1.2700	-0.0072	-0.0038	12.1256	0.9178
	V	5.895	1.2013				
