# Supplementary material for: The varying temperature- and pressure-induced phase transition pathways in hybrid improper ferroelectric Sr3Sn2O7
Source: Acta Crystallogr B Struct Sci Cryst Eng Mater. 2025 Apr 14;81(Pt 3):318–24. doi: 10.1107/S2052520625002306 (PMC12147935; doi:10.1107/S2052520625002306)

checkCIF/PLATON report

Structure factors have been supplied for datablock(s) 013_sso_x1_p9_30-5-1

THIS REPORT IS FOR GUIDANCE ONLY. IF USED AS PART OF A REVIEW PROCEDURE FOR PUBLICATION, IT SHOULD NOT REPLACE THE EXPERTISE OF AN EXPERIENCED CRYSTALLOGRAPHIC REFEREE.

No syntax errors found.   CIF dictionary   Interpreting this report

Datablock: 013_sso_x1_p9_30-5-1

```
Bond precision:   Sn- O = 0.0170 A         Wavelength=0.48590

Cell:                a=5.6737(6)      b=5.7002(6)      c=20.43(10)
                     alpha=90         beta=90          gamma=90
Temperature:         300 K

                 Calculated                    Reported
Volume           661(3)                        661(3)
Space group      A 21 a m                      A 21 a m
Hall group       A -2 2a                       A -2 2a
Moiety formula   O28 Sn8, 12(Sr)               O7 Sn2 Sr3
Sum formula      O28 Sn8 Sr12                  O7 Sn2 Sr3
Mr               2449.12                       612.24
Dx,g cm-3        6.150                         6.156
Z                1                             4
Mu (mm-1)        11.483                        11.486
F000             1080.0                        1080.0
F000'            1068.45
h,k,lmax         8,8,28                        7,7,4
Nref             1015[ 558]                    174
Tmin,Tmax                                      0.706,0.713
Tmin'

Correction method= # Reported T Limits: Tmin=0.706 Tmax=0.713
AbsCorr = SPHERE

Data completeness= 0.31/0.17       Theta(max)= 20.222

R(reflections)= 0.0413( 161)                            wR2(reflections)=
                                                        0.1017( 174)
S = 1.156                 Npar= 28
```

The following ALERTS were generated. Each ALERT has the format
test-name_ALERT_alert-type_alert-level.
Click on the hyperlinks for more details of the test.

Alert level A

ATOM007_ALERT_1_A _atom_site_aniso_label is missing
Unique label identifying the atom site.

Author Response: As the measurement was performed using a diamond anvil cell to achieve high pressures, the available region of reciprocal space is restricted, resulting in a low data/parameter ratio. Therefore, an anisotropic model is neither feasible nor advisable

PLAT029_ALERT_3_A _diffrn_measured_fraction_theta_full value Low . 0.233 Why?

Author Response: The measurement was performed using a diamond anvil cell, of which the precise design necessitates a small opening angle (approx. 23 degrees) to obtain high pressures. This restricts the available region of reciprocal space.

PLAT090_ALERT_3_A Poor Data / Parameter Ratio (Zmax > 18) .... 3.61 Note

Author Response: The measurement was performed using a diamond anvil cell, of which the precise design necessitates a small opening angle (approx. 23 degrees) to obtain high pressures. This restricts the available region of reciprocal space. The sample was refined isotropically to improve this.

PLAT109_ALERT_2_A Twinning Matrix = Alternate Inversion Twinning . ? Check

Author Response: The present system stems from a cetrosymmetric, tetragonal aristotype. This can be modelled using a twin law given by (0 1 0 -1 0 0 0 0 1 4). The two-fold twin was subsequently chosen over the inversion twin law to explicitly depict the twinning present as viewed from the perspective of the relevant properties (i.e., hybrid improper ferroelectric polarisation).

Alert level B

PLAT196_ALERT_1_B No TEMP record and measurement temperature .NE. 293 Degree
Sn1 O1 O2 O3 O4

Author Response: Model incorporates no riding hydrogens so no TEMP record is required.

Alert level C

```
PLAT041_ALERT_1_C Calc. and Reported SumFormula    Strings  Differ     Please Check
              Calc: O28 Sn8 Sr12
              Rep.: O7 Sn2 Sr3
PLAT042_ALERT_1_C Calc. and Reported MoietyFormula Strings  Differ     Please Check
              Calc: O28 Sn8, 12(Sr)
              Rep.: O7 Sn2 Sr3
PLAT094_ALERT_2_C Ratio of Maximum / Minimum Residual Density ....       2.01 Report
PLAT148_ALERT_3_C s.u. on the      c    - Axis is (Too) Large ....      0.100 Ang.
PLAT202_ALERT_3_C Isotropic non-H Atoms in Anion/Solvent ........          2 Check
              Sr1     Sr2
PLAT911_ALERT_3_C Missing FCF Refl Between Thmin & STh/L=    0.600         19 Report
                0  6  2,   0  1  3,   1  1  3,   2  1  3,   0  0  4,   2  0  4,
                4  0  4,   6  0  4,   0  2  4,   1  2  4,   2  2  4,   3  2  4,
                6  2  4,   0  4  4,   5  4  4,   0  6  4,   1  6  4,   2  6  4,
                3  6  4,
PLAT939_ALERT_3_C Large Value of Not (SHELXL) Weight Optimized S .      13.80 Check
PLAT975_ALERT_2_C Check Calcd Resid. Dens.  0.74Ang From O2      .       0.88 eA-3
```

Alert level G

```
ABSMU01_ALERT_1_G  Calculation of _exptl_absorpt_correction_mu
                not performed for this radiation type.
PLAT002_ALERT_2_G Number of Distance or Angle Restraints on AtSite          3 Note
PLAT003_ALERT_2_G Number of Uiso or U(i,j)  Restrained non-H-Atoms          3 Report
PLAT004_ALERT_5_G Polymeric Structure Found with Maximum Dimension          2 Info
PLAT045_ALERT_1_G Calculated and Reported Z Differ by a Factor ...      0.250 Check
PLAT111_ALERT_2_G ADDSYM Detects New (Pseudo) Centre of Symmetry .        100 %Fit
PLAT112_ALERT_2_G ADDSYM Detects New (Pseudo) Symm. Elem         m        100 %Fit
PLAT113_ALERT_2_G ADDSYM Suggests Possible Pseudo/New  Space Group       Cmcm Check
              Check Model Parameter Symmetry for Reflection Data Support
PLAT176_ALERT_4_G The CIF-Embedded .res File Contains SADI Records          1 Report
PLAT178_ALERT_4_G The CIF-Embedded .res File Contains SIMU Records          1 Report
PLAT188_ALERT_3_G A Non-default SIMU Restraint Value has been used     0.0020 Report
PLAT794_ALERT_5_G Tentative Bond Valency for Sn1       (IV)      .       4.35 Info
PLAT860_ALERT_3_G Number of Least-Squares Restraints ............          4 Note
PLAT910_ALERT_3_G Missing # of FCF Reflection(s) Below Theta(Min).          1 Note
                0  0  2,
PLAT912_ALERT_4_G Missing # of FCF Reflections Above STh/L=  0.600         10 Note
PLAT915_ALERT_3_G No Flack x Check Done: Low Friedel Pair Coverage         16 %
PLAT916_ALERT_2_G Hooft y and Flack x Parameter Values Differ by .       0.50 Check
PLAT941_ALERT_3_G Average HKL Measurement Multiplicity ..........         4.1 Low
PLAT952_ALERT_5_G Calculated (ThMax) and CIF-Reported Lmax Differ.         24 Units
PLAT958_ALERT_1_G Calculated (ThMax) and Actual (FCF) Lmax Differ.         24 Units
PLAT961_ALERT_5_G Dataset Contains no Negative Intensities ......     Please Check
PLAT969_ALERT_5_G The 'Henn et al.' R-Factor-gap value ..........     14.526 Note
              Predicted wR2: Based on SigI**2  0.70 or SHELX Weight  8.80
```

4 **ALERT level A** = Most likely a serious problem - resolve or explain
1 **ALERT level B** = A potentially serious problem, consider carefully
8 **ALERT level C** = Check. Ensure it is not caused by an omission or oversight
22 **ALERT level G** = General information/check it is not something unexpected

7 ALERT type 1 CIF construction/syntax error, inconsistent or missing data

```
  9 ALERT type 2 Indicator that the structure model may be wrong or deficient
 11 ALERT type 3 Indicator that the structure quality may be low
  3 ALERT type 4 Improvement, methodology, query or suggestion
  5 ALERT type 5 Informative message, check
```

---

checkCIF publication errors

Alert level A

```
PUBL004_ALERT_1_A  The contact author's name and address are missing,
            _publ_contact_author_name and _publ_contact_author_address.
PUBL005_ALERT_1_A  _publ_contact_author_email, _publ_contact_author_fax and
            _publ_contact_author_phone are all missing.
            At least one of these should be present.
PUBL006_ALERT_1_A  _publ_requested_journal is missing
            e.g. 'Acta Crystallographica Section C'
PUBL008_ALERT_1_A  _publ_section_title is missing. Title of paper.
PUBL009_ALERT_1_A  _publ_author_name is missing. List of author(s) name(s).
PUBL010_ALERT_1_A  _publ_author_address is missing. Author(s) address(es).
PUBL012_ALERT_1_A  _publ_section_abstract is missing.
            Abstract of paper in English.
```

---

7 **ALERT level A** = Data missing that is essential or data in wrong format
0 **ALERT level G** = General alerts. Data that may be required is missing

---

Publication of your CIF

You should attempt to resolve as many as possible of the alerts in all categories. Often the minor alerts point to easily fixed oversights, errors and omissions in your CIF or refinement strategy, so attention to these fine details can be worthwhile. In order to resolve some of the more serious problems it may be necessary to carry out additional measurements or structure refinements. However, the nature of your study may justify the reported deviations from journal submission requirements and the more serious of these should be commented upon in the discussion or experimental section of a paper or in the "special_details" fields of the CIF. *checkCIF* was carefully designed to identify outliers and unusual parameters, but every test has its limitations and alerts that are not important in a particular case may appear. Conversely, the absence of alerts does not guarantee there are no aspects of the results needing attention. It is up to the individual to critically assess their own results and, if necessary, seek expert advice.

If level A alerts remain, which you believe to be justified deviations, and you intend to submit this CIF for publication in a journal, you should additionally insert an explanation in your CIF using the Validation Reply Form (VRF) below. This will allow your explanation to be considered as part of the review process.

```
# start Validation Reply Form
_vrf_PUBL004_GLOBAL
;
PROBLEM: The contact author's name and address are missing,
RESPONSE: ...
;
_vrf_PUBL005_GLOBAL
;
PROBLEM: _publ_contact_author_email, _publ_contact_author_fax and
RESPONSE: ...
;
_vrf_PUBL006_GLOBAL
;
PROBLEM: _publ_requested_journal is missing
RESPONSE: ...
;
_vrf_PUBL008_GLOBAL
;
PROBLEM: _publ_section_title is missing. Title of paper.
RESPONSE: ...
;
_vrf_PUBL009_GLOBAL
;
PROBLEM: _publ_author_name is missing. List of author(s) name(s).
RESPONSE: ...
;
_vrf_PUBL010_GLOBAL
;
PROBLEM: _publ_author_address is missing. Author(s) address(es).
RESPONSE: ...
;
_vrf_PUBL012_GLOBAL
;
PROBLEM: _publ_section_abstract is missing.
RESPONSE: ...
;
# end Validation Reply Form
```

If you wish to submit your CIF for publication in Acta Crystallographica Section C or E, you should upload your CIF via the web. If you wish to submit your CIF for publication in IUCrData you should upload your CIF via the web. If your CIF is to form part of a submission to another IUCr journal, you will be asked, either during electronic submission or by the Co-editor handling your paper, to upload your CIF via our web site.

PLATON version of 19/12/2024; check.def file version of 19/12/2024

Datablock 013_sso_x1_p9_30-5-1 - ellipsoid plot

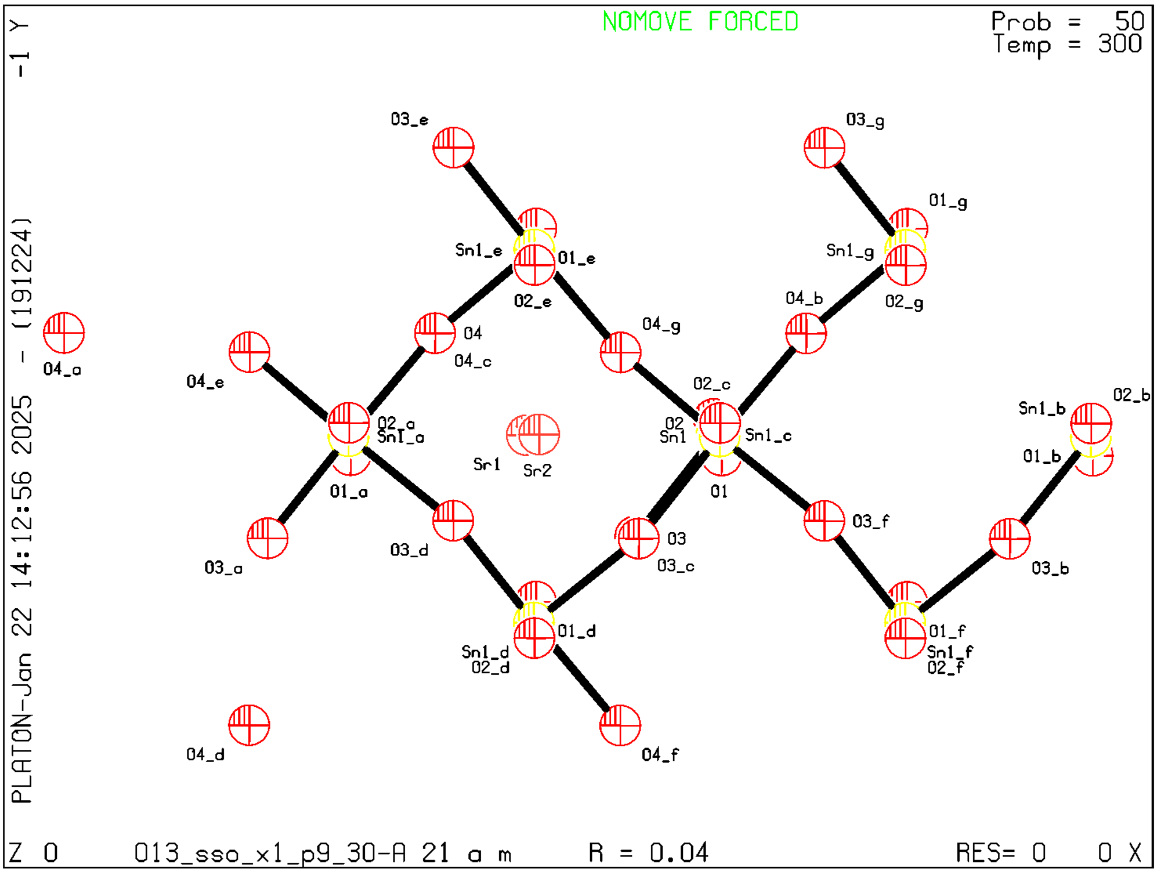

Supplement: Supplementary file 1 [file 013_SSO_x1_P9_30-5-1_checkcif.pdf › SSO_HP_VT_CIFs/SSO_HP_CIFS/013_SSO_x1_P9_30-5-1_checkcif.pdf]
