# Supplementary material for: The varying temperature- and pressure-induced phase transition pathways in hybrid improper ferroelectric Sr3Sn2O7
Source: Acta Crystallogr B Struct Sci Cryst Eng Mater. 2025 Apr 14;81(Pt 3):318–24. doi: 10.1107/S2052520625002306 (PMC12147935; doi:10.1107/S2052520625002306)

checkCIF/PLATON report

Structure factors have been supplied for datablock(s) 016_sso_x1_p12_30-5-1_225

THIS REPORT IS FOR GUIDANCE ONLY. IF USED AS PART OF A REVIEW PROCEDURE FOR PUBLICATION, IT SHOULD NOT REPLACE THE EXPERTISE OF AN EXPERIENCED CRYSTALLOGRAPHIC REFEREE.

No syntax errors found.   CIF dictionary   Interpreting this report

Datablock: 016_sso_x1_p12_30-5-1_225

Bond precision:	Sn- O = 0.0250 A		Wavelength=0.48590
Cell:	a=5.6133(8)	b=5.6446(8)	c=20.10(15)
	alpha=90	beta=90	gamma=90
Temperature:	300 K		

	Calculated	Reported
Volume	637(5)	637(5)
Space group	A 21 a m	A 21 a m
Hall group	A -2 2a	A -2 2a
Moiety formula	O28 Sn8, 12(Sr)	O7 Sn2 Sr3
Sum formula	O28 Sn8 Sr12	O7 Sn2 Sr3
Mr	2449.12	612.24
Dx,g cm-3	6.380	6.386
Z	1	4
Mu (mm-1)	11.915	11.915
F000	1080.0	1080.0
F000’	1068.45	
h,k,lmax	8,8,28	7,7,4
Nref	1006[553]	155
Tmin,Tmax		1.000,1.000
Tmin’		

Correction method= # Reported T Limits: Tmin=1.000 Tmax=1.000
AbsCorr = SPHERE

Data completeness= 0.28/0.15   Theta(max)= 20.445

R(reflections)= 0.0559(147)   wR2(reflections)= 0.1259(155)

S = 1.213   Npar= 28

The following ALERTS were generated. Each ALERT has the format
test-name_ALERT_alert-type_alert-level.
Click on the hyperlinks for more details of the test.

Alert level A

```
ATOM007_ALERT_1_A  _atom_site_aniso_label is missing
            Unique label identifying the atom site.
```

Author Response: As the measurement was performed using a diamond anvil cell to achieve high pressures, the available region of reciprocal space is restricted, resulting in a low data/parameter ratio. Therefore, an anisotropic model is neither feasible nor advisable.

```
PLAT029_ALERT_3_A _diffrn_measured_fraction_theta_full value Low .      0.220 Why?
```

Author Response: The measurement was performed using a diamond anvil cell, of which the precise design necessitates a small opening angle (approx. 23 degrees) to obtain high pressures. This restricts the available region of reciprocal space.

```
PLAT090_ALERT_3_A Poor Data / Parameter Ratio (Zmax > 18) .......       3.18 Note
```

Author Response: The measurement was performed using a diamond anvil cell, of which the precise design necessitates a small opening angle (approx. 23 degrees) to obtain high pressures. This restricts the available region of reciprocal space. The sample was refined isotropically to improve this.

```
PLAT109_ALERT_2_A Twinning Matrix = Alternate Inversion Twinning .          ? Check
```

Author Response: The present system stems from a cetrosymmetric, tetragonal aristotype. This can be modelled using a twin law given by (0 1 0 -1 0 0 0 0 1 4). The two-fold twin was subsequently chosen over the inversion twin law to explicitly depict the twinning present as viewed from the perspective of the relevant properties (i.e., hybrid improper ferroelectric polarisation).

Alert level B

```
PLAT196_ALERT_1_B No TEMP record and measurement temperature .NE.        293 Degree
              Sn1      O1      O2      O3      O4
```

Author Response: Model incorporates no riding hydrogens so no TEMP record is required.

Alert level C

```
PLAT041_ALERT_1_C Calc. and Reported SumFormula    Strings  Differ     Please Check
              Calc: O28 Sn8 Sr12
              Rep.: O7 Sn2 Sr3
PLAT042_ALERT_1_C Calc. and Reported MoietyFormula Strings  Differ     Please Check
              Calc: O28 Sn8, 12(Sr)
              Rep.: O7 Sn2 Sr3
PLAT148_ALERT_3_C s.u. on the      c    - Axis is (Too) Large ....      0.150 Ang.
PLAT202_ALERT_3_C Isotropic non-H Atoms in Anion/Solvent .........          2 Check
              Sr1     Sr2
PLAT911_ALERT_3_C Missing FCF Refl Between Thmin & STh/L=    0.600         23 Report
                0  2  2,   0  6  2,   0  1  3,   1  1  3,   2  1  3,   0  0  4,
                2  0  4,   4  0  4,   6  0  4,   0  2  4,   1  2  4,   2  2  4,
                3  2  4,   4  2  4,   5  2  4,   6  2  4,   0  4  4,   1  4  4,
                2  4  4,   5  4  4,   0  6  4,   1  6  4,   2  6  4,
PLAT975_ALERT_2_C Check Calcd Resid. Dens.  0.89Ang From O1      .       0.99 eA-3
PLAT975_ALERT_2_C Check Calcd Resid. Dens.  0.74Ang From O2      .       0.95 eA-3
PLAT975_ALERT_2_C Check Calcd Resid. Dens.  0.75Ang From O3      .       0.86 eA-3
PLAT976_ALERT_2_C Check Calcd Resid. Dens.  0.99Ang From O4      .      -0.63 eA-3
PLAT976_ALERT_2_C Check Calcd Resid. Dens.  0.97Ang From O2      .      -0.43 eA-3
```

Alert level G

```
ABSMU01_ALERT_1_G  Calculation of _exptl_absorpt_correction_mu
                not performed for this radiation type.
PLAT002_ALERT_2_G Number of Distance or Angle Restraints on AtSite          3 Note
PLAT003_ALERT_2_G Number of Uiso or U(i,j)  Restrained non-H-Atoms          3 Report
PLAT004_ALERT_5_G Polymeric Structure Found with Maximum Dimension          2 Info
PLAT045_ALERT_1_G Calculated and Reported Z Differ by a Factor ...      0.250 Check
PLAT111_ALERT_2_G ADDSYM Detects New (Pseudo) Centre of Symmetry .        100 %Fit
PLAT112_ALERT_2_G ADDSYM Detects New (Pseudo) Symm. Elem         m        100 %Fit
PLAT113_ALERT_2_G ADDSYM Suggests Possible Pseudo/New  Space Group       Cmcm Check
              Check Model Parameter Symmetry for Reflection Data Support
PLAT176_ALERT_4_G The CIF-Embedded .res File Contains SADI Records          1 Report
PLAT178_ALERT_4_G The CIF-Embedded .res File Contains SIMU Records          1 Report
PLAT188_ALERT_3_G A Non-default SIMU Restraint Value has been used     0.0020 Report
PLAT794_ALERT_5_G Tentative Bond Valency for Sn1       (IV)      .       4.40 Info
PLAT860_ALERT_3_G Number of Least-Squares Restraints .............          4 Note
PLAT910_ALERT_3_G Missing # of FCF Reflection(s) Below Theta(Min).          1 Note
                0  0  2,
PLAT912_ALERT_4_G Missing # of FCF Reflections Above STh/L=  0.600         18 Note
PLAT915_ALERT_3_G No Flack x Check Done: Low Friedel Pair Coverage         15 %
PLAT916_ALERT_2_G Hooft y and Flack x Parameter Values Differ by .       0.49 Check
PLAT933_ALERT_2_G Number of HKL-OMIT Records in Embedded .res File          1 Note
                5  0  0,
PLAT941_ALERT_3_G Average HKL Measurement Multiplicity ...........        4.0 Low
PLAT952_ALERT_5_G Calculated (ThMax) and CIF-Reported Lmax Differ.         24 Units
PLAT958_ALERT_1_G Calculated (ThMax) and Actual (FCF) Lmax Differ.         24 Units
PLAT969_ALERT_5_G The 'Henn et al.' R-Factor-gap value ..........       6.797 Note
              Predicted wR2: Based on SigI**2  1.85 or SHELX Weight 10.38
```

4 **ALERT level A** = Most likely a serious problem - resolve or explain
1 **ALERT level B** = A potentially serious problem, consider carefully
10 **ALERT level C** = Check. Ensure it is not caused by an omission or oversight

```
22 ALERT level G = General information/check it is not something unexpected

 7 ALERT type 1 CIF construction/syntax error, inconsistent or missing data
13 ALERT type 2 Indicator that the structure model may be wrong or deficient
10 ALERT type 3 Indicator that the structure quality may be low
 3 ALERT type 4 Improvement, methodology, query or suggestion
 4 ALERT type 5 Informative message, check
```

It is advisable to attempt to resolve as many as possible of the alerts in all categories. Often the minor alerts point to easily fixed oversights, errors and omissions in your CIF or refinement strategy, so attention to these fine details can be worthwhile. In order to resolve some of the more serious problems it may be necessary to carry out additional measurements or structure refinements. However, the purpose of your study may justify the reported deviations and the more serious of these should normally be commented upon in the discussion or experimental section of a paper or in the "special_details" fields of the CIF. checkCIF was carefully designed to identify outliers and unusual parameters, but every test has its limitations and alerts that are not important in a particular case may appear. Conversely, the absence of alerts does not guarantee there are no aspects of the results needing attention. It is up to the individual to critically assess their own results and, if necessary, seek expert advice.

Publication of your CIF in IUCr journals

A basic structural check has been run on your CIF. These basic checks will be run on all CIFs submitted for publication in IUCr journals (*Acta Crystallographica*, *Journal of Applied Crystallography*, *Journal of Synchrotron Radiation*); however, if you intend to submit to *Acta Crystallographica Section C* or *E* or *IUCrData*, you should make sure that full publication checks are run on the final version of your CIF prior to submission.

Publication of your CIF in other journals

Please refer to the *Notes for Authors* of the relevant journal for any special instructions relating to CIF submission.

PLATON version of 19/12/2024; check.def file version of 19/12/2024

Datablock 016_sso_x1_p12_30-5-1_225 - ellipsoid plot

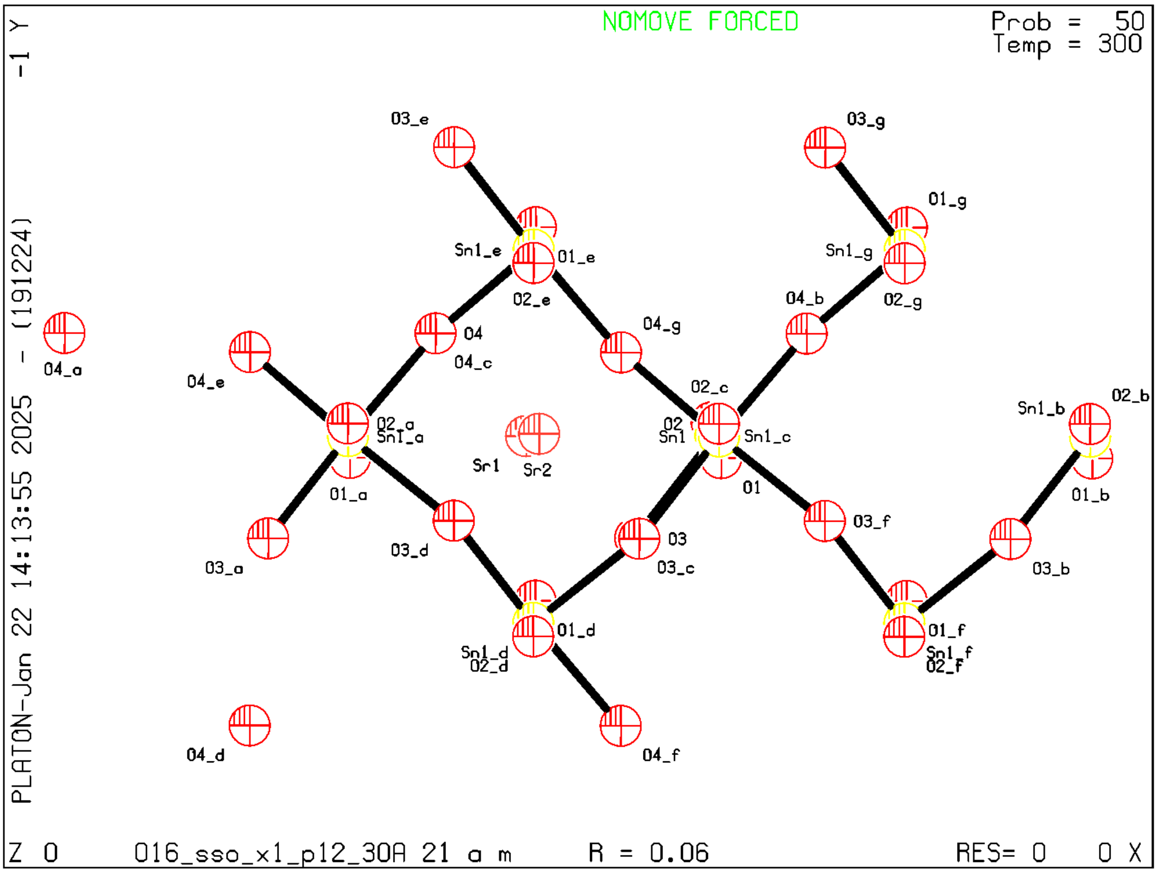

Supplement: Supplementary file 1 [file 016_SSO_x1_P12_30-5-1_225_checkcif.pdf › SSO_HP_VT_CIFs/SSO_HP_CIFS/016_SSO_x1_P12_30-5-1_225_checkcif.pdf]
