# Supplementary material for: The varying temperature- and pressure-induced phase transition pathways in hybrid improper ferroelectric Sr3Sn2O7
Source: Acta Crystallogr B Struct Sci Cryst Eng Mater. 2025 Apr 14;81(Pt 3):318–24. doi: 10.1107/S2052520625002306 (PMC12147935; doi:10.1107/S2052520625002306)

checkCIF/PLATON report

Structure factors have been supplied for datablock(s) 037_sso_x1_p33_30-5-1

THIS REPORT IS FOR GUIDANCE ONLY. IF USED AS PART OF A REVIEW PROCEDURE FOR PUBLICATION, IT SHOULD NOT REPLACE THE EXPERTISE OF AN EXPERIENCED CRYSTALLOGRAPHIC REFEREE.

No syntax errors found. CIF dictionary Interpreting this report

Datablock: 037_sso_x1_p33_30-5-1

```
Bond precision:   Sn- O = 0.0148 A         Wavelength=0.48590

Cell:               a=5.3873(6)       b=5.3891(6)       c=20.26(9)
                    alpha=90          beta=90           gamma=90
Temperature:        300 K

                 Calculated                  Reported
Volume           588(3)                      588(3)
Space group      A c a a                     A c a a
Hall group       -A 2ac 2c                   -A 2ac 2c
Moiety formula   O14 Sn4, 6(Sr)              O7 Sn2 Sr3
Sum formula      O14 Sn4 Sr6                 O7 Sn2 Sr3
Mr               1224.56                     612.24
Dx,g cm-3        6.920                       6.912
Z                2                           4
Mu (mm-1)        12.908                      12.897
F000             1080.0                      1080.0
F000'            1068.45
h,k,lmax         8,8,30                      7,7,4
Nref             517                         80
Tmin,Tmax                                    0.006,0.015
Tmin'

Correction method= # Reported T Limits: Tmin=0.006 Tmax=0.015
AbsCorr = SPHERE

Data completeness= 0.155          Theta(max)= 21.327

                                                      wR2(reflections)=
R(reflections)= 0.0221( 69)                           0.0678( 80)

S = 1.201                  Npar= 14
```

```
The following ALERTS were generated. Each ALERT has the format
       test-name_ALERT_alert-type_alert-level.
Click on the hyperlinks for more details of the test.
```

Alert level A

```
ATOM007_ALERT_1_A  _atom_site_aniso_label is missing
            Unique label identifying the atom site.
```

Author Response: As the measurement was performed using a diamond anvil cell to achieve high pressures, the available region of reciprocal space is restricted, resulting in a low data/parameter ratio. Therefore, an anisotropic model is neither feasible nor advisable.

```
PLAT029_ALERT_3_A _diffrn_measured_fraction_theta_full value Low .      0.238 Why?
```

Author Response: The measurement was performed using a diamond anvil cell, of which the precise design necessitates a small opening angle (approx. 23 degrees) to obtain high pressures. This restricts the available region of reciprocal space.

```
PLAT088_ALERT_3_A Poor Data / Parameter Ratio ....................       5.71 Note
```

Author Response: The measurement was performed using a diamond anvil cell, of which the precise design necessitates a small opening angle (approx. 23 degrees) to obtain high pressures. This restricts the available region of reciprocal space. The sample was refined isotropically to improve this.

Alert level C

```
PLAT041_ALERT_1_C Calc. and Reported SumFormula    Strings  Differ     Please Check
              Calc: O14 Sn4 Sr6
              Rep.: O7 Sn2 Sr3
PLAT042_ALERT_1_C Calc. and Reported MoietyFormula Strings  Differ     Please Check
              Calc: O14 Sn4, 6(Sr)
              Rep.: O7 Sn2 Sr3
PLAT148_ALERT_3_C s.u. on the      c    - Axis is (Too) Large ....      0.090 Ang.
PLAT202_ALERT_3_C Isotropic non-H Atoms in Anion/Solvent .........          2 Check
              Sr1     Sr2
PLAT906_ALERT_3_C Large K Value in the Analysis of Variance ......    135.452 Check
PLAT911_ALERT_3_C Missing FCF Refl Between Thmin & STh/L=    0.600         14 Report
                1  1  3,   2  1  3,   2  0  4,   4  0  4,   6  0  4,   0  2  4,
                1  2  4,   2  2  4,   3  2  4,   6  2  4,   0  4  4,   0  6  4,
                1  6  4,   2  6  4,
```

Alert level G

```
ABSMU01_ALERT_1_G  Calculation of _exptl_absorpt_correction_mu
```

```
                not performed for this radiation type.
PLAT002_ALERT_2_G Number of Distance or Angle Restraints on AtSite          3 Note
PLAT003_ALERT_2_G Number of Uiso or U(i,j)  Restrained non-H-Atoms          3 Report
PLAT004_ALERT_5_G Polymeric Structure Found with Maximum Dimension          2 Info
PLAT045_ALERT_1_G Calculated and Reported Z Differ by a Factor ...      0.500 Check
PLAT128_ALERT_4_G Alternate Setting for Input Space Group  Acaa          Ccca Note
PLAT176_ALERT_4_G The CIF-Embedded .res File Contains SADI Records          1 Report
PLAT178_ALERT_4_G The CIF-Embedded .res File Contains SIMU Records          1 Report
PLAT188_ALERT_3_G A Non-default SIMU Restraint Value has been used     0.0020 Report
PLAT191_ALERT_3_G A Non-default SADI Restraint Value has been used     0.1000 Report
PLAT794_ALERT_5_G Tentative Bond Valency for Sn1       (IV)      .       4.99 Info
PLAT860_ALERT_3_G Number of Least-Squares Restraints ............           3 Note
PLAT870_ALERT_4_G ALERTS Related to Twinning Effects Suppressed ..          ! Info
PLAT910_ALERT_3_G Missing # of FCF Reflection(s) Below Theta(Min).          2 Note
                0  0  2,   0  0  4,
PLAT912_ALERT_4_G Missing # of FCF Reflections Above STh/L=  0.600         15 Note
PLAT941_ALERT_3_G Average HKL Measurement Multiplicity ..........         4.1 Low
PLAT952_ALERT_5_G Calculated (ThMax) and CIF-Reported Lmax Differ.         26 Units
PLAT958_ALERT_1_G Calculated (ThMax) and Actual (FCF) Lmax Differ.         26 Units
PLAT961_ALERT_5_G Dataset Contains no Negative Intensities .......     Please Check
PLAT969_ALERT_5_G The 'Henn et al.' R-Factor-gap value ..........      10.044 Note
              Predicted wR2: Based on SigI**2  0.68 or SHELX Weight  5.65
```

```
   3 ALERT level A = Most likely a serious problem - resolve or explain
   0 ALERT level B = A potentially serious problem, consider carefully
   6 ALERT level C = Check. Ensure it is not caused by an omission or oversight
  20 ALERT level G = General information/check it is not something unexpected

   6 ALERT type 1 CIF construction/syntax error, inconsistent or missing data
   2 ALERT type 2 Indicator that the structure model may be wrong or deficient
  11 ALERT type 3 Indicator that the structure quality may be low
   5 ALERT type 4 Improvement, methodology, query or suggestion
   5 ALERT type 5 Informative message, check
```

It is advisable to attempt to resolve as many as possible of the alerts in all categories. Often the minor alerts point to easily fixed oversights, errors and omissions in your CIF or refinement strategy, so attention to these fine details can be worthwhile. In order to resolve some of the more serious problems it may be necessary to carry out additional measurements or structure refinements. However, the purpose of your study may justify the reported deviations and the more serious of these should normally be commented upon in the discussion or experimental section of a paper or in the "special_details" fields of the CIF. checkCIF was carefully designed to identify outliers and unusual parameters, but every test has its limitations and alerts that are not important in a particular case may appear. Conversely, the absence of alerts does not guarantee there are no aspects of the results needing attention. It is up to the individual to critically assess their own results and, if necessary, seek expert advice.

Publication of your CIF in IUCr journals

A basic structural check has been run on your CIF. These basic checks will be run on all CIFs submitted for publication in IUCr journals (*Acta Crystallographica*, *Journal of Applied Crystallography*, *Journal of Synchrotron Radiation*); however, if you intend to submit to *Acta Crystallographica Section C* or *E* or *IUCrData*, you should make sure that full publication checks are run on the final version of your CIF prior to submission.

Publication of your CIF in other journals

Please refer to the *Notes for Authors* of the relevant journal for any special instructions relating to CIF submission.

PLATON version of 19/12/2024; check.def file version of 19/12/2024

Datablock 037_sso_x1_p33_30-5-1 - ellipsoid plot

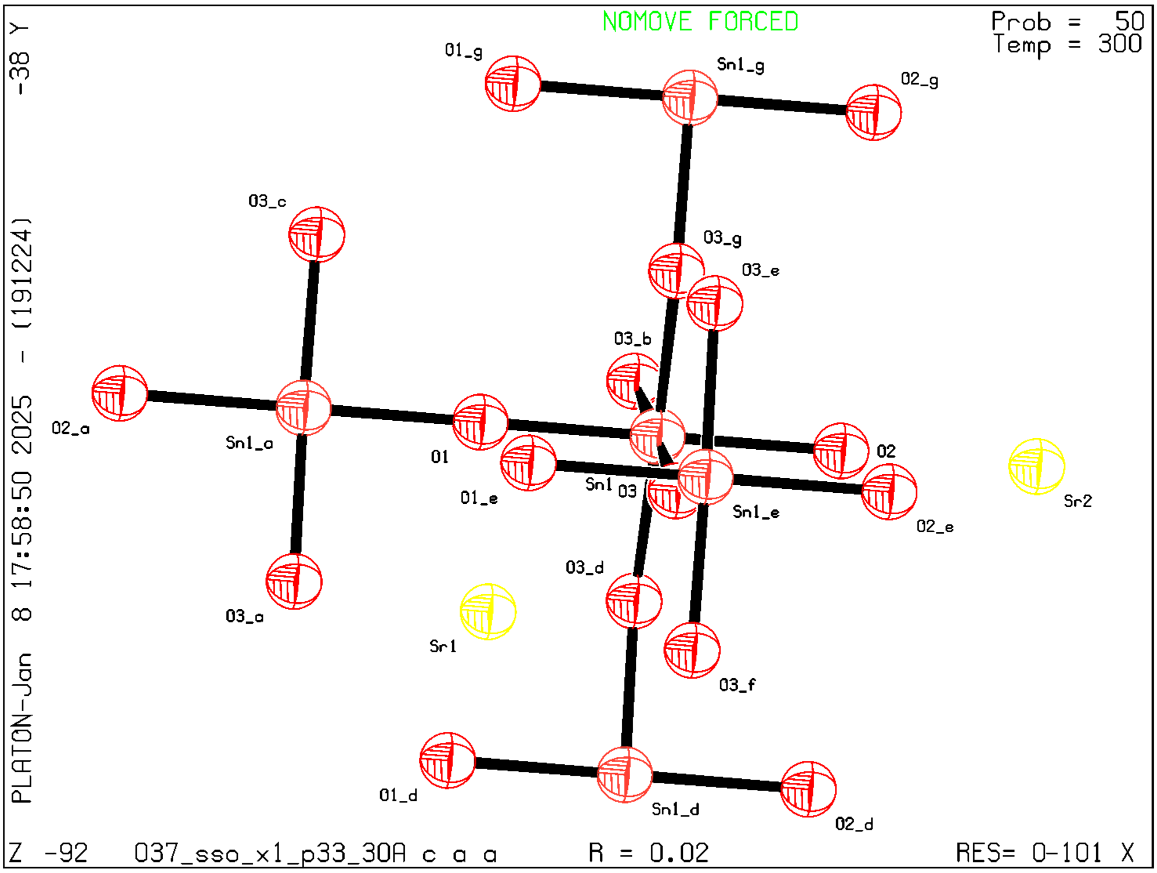

Supplement: Supplementary file 1 [file 037_SSO_x1_P33_30-5-1_checkcif.pdf › SSO_HP_VT_CIFs/SSO_HP_CIFS/037_SSO_x1_P33_30-5-1_checkcif.pdf]
