# Supplementary material for: The varying temperature- and pressure-induced phase transition pathways in hybrid improper ferroelectric Sr3Sn2O7
Source: Acta Crystallogr B Struct Sci Cryst Eng Mater. 2025 Apr 14;81(Pt 3):318–24. doi: 10.1107/S2052520625002306 (PMC12147935; doi:10.1107/S2052520625002306)

checkCIF/PLATON report

Structure factors have been supplied for datablock(s) srsno_i19vt_01_100_2_a21am

THIS REPORT IS FOR GUIDANCE ONLY. IF USED AS PART OF A REVIEW PROCEDURE FOR PUBLICATION, IT SHOULD NOT REPLACE THE EXPERTISE OF AN EXPERIENCED CRYSTALLOGRAPHIC REFEREE.

No syntax errors found.   CIF dictionary   Interpreting this report

Datablock: srsno_i19vt_01_100_2_a21am

Bond precision:	Sn- O = 0.0073 A	Wavelength=0.68890	
Cell:	a=5.69077(13)	b=5.71964(14)	c=20.6244(5)
	alpha=90	beta=90	gamma=90
Temperature:	100 K		

	Calculated	Reported
Volume	671.31(3)	671.31(3)
Space group	A 21 a m	A 21 a m
Hall group	A -2 2a	A -2 2a
Moiety formula	O28 Sn8, 12(Sr)	O7 Sn2 Sr3
Sum formula	O28 Sn8 Sr12	O7 Sn2 Sr3
Mr	2449.12	612.24
Dx,g cm-3	6.058	6.058
Z	1	4
Mu (mm-1)	28.242	28.442
F000	1080.0	1080.0
F000’	1061.72	
h,k,lmax	9,9,33	9,9,33
Nref	1553[843]	1446
Tmin,Tmax		0.001,0.011
Tmin’		

Correction method= # Reported T Limits: Tmin=0.001 Tmax=0.011
AbsCorr = SPHERE

Data completeness= 1.72/0.93   Theta(max)= 34.112

R(reflections)= 0.0709(1332)   wR2(reflections)= 0.1846(1446)

S = 1.129   Npar= 59

The following ALERTS were generated. Each ALERT has the format
test-name_ALERT_alert-type_alert-level.
Click on the hyperlinks for more details of the test.

Alert level A

PLAT109_ALERT_2_A Twinning Matrix = Alternate Inversion Twinning . ? Check

Author Response: The present system stems from a cetrosymmetric, tetragonal aristotype. This can be modelled using a twin law given by (0 1 0 -1 0 0 0 0 1 4). The two-fold twin was subsequently chosen over the inversion twin law to explicitly depict the twinning present as viewed from the perspective of the relevant properties (i.e., hybrid improper ferroelectric polarisation).

PLAT971_ALERT_2_A Check Calcd Resid. Dens. 0.60Ang From Sn1 5.96 eA-3

Author Response: The sample is highly absorbing and contains a significant proportion of heavy elements. While efforts have been made to account for this as best as possible, the diffractometer upon which the data were collected lacks a camera with which to obtain a true representation of the crystal for a more explicit correction. The impact of this upon the data is further compounded by the crystal existing as a highly anisotropic plate. Errors carried through from this in the intensities will increase the residual levels in the model. This is in addition to the typical fourier rippling that is expected at heavy elements, and the impact of the spherical contraction of the valence shell owed to the ionisation of these metals in this highly charge system. Since these residuals exist close to these heavy metal centres and the model (and symmetry) has been thoroughly evaluated (see the manuscript), these are believed to have no physical significance.

PLAT971_ALERT_2_A Check Calcd Resid. Dens. 0.59Ang From Sn1 5.90 eA-3

Author Response: The sample is highly absorbing and contains a significant proportion of heavy elements. While efforts have been made to account for this as best as possible, the diffractometer upon which the data were collected lacks a camera with which to obtain a true representation of the crystal for a more explicit correction. The impact of this upon the data is further compounded by the crystal existing as a highly anisotropic plate. Errors carried through from this in the intensities will increase the residual levels in the model. This is in addition to the typical fourier rippling that is expected at heavy elements, and the impact of the spherical contraction of the valence shell owed to the ionisation of these metals in this highly charge system. Since these residuals exist close to these heavy metal centres and the model (and symmetry) has been thoroughly evaluated (see the manuscript), these are believed to have no physical significance.

PLAT971_ALERT_2_A Check Calcd Resid. Dens. 0.63Ang From Sn1 5.84 eA-3

Author Response: The sample is highly absorbing and contains a significant proportion of heavy elements. While efforts have been made to account for this as best as possible, the diffractometer upon which the data were collected lacks a camera with which to obtain a true representation of the crystal for a more explicit correction. The impact of this upon the data is further compounded by the crystal existing as a highly anisotropic plate. Errors carried through from this in the intensities will increase the residual levels in the model. This is in addition to the typical fourier rippling that is expected at heavy elements, and the impact of the spherical contraction of the valence shell owed to the ionisation of these metals in this highly charge system. Since these residuals exist close to these heavy metal centres and the model (and symmetry) has been thoroughly evaluated (see the manuscript), these are believed to have no physical significance.

PLAT971_ALERT_2_A Check Calcd Resid. Dens. 0.62Ang From Sn1 5.80 eA-3

Author Response: The sample is highly absorbing and contains a significant proportion of heavy elements. While efforts have been made to account for this as best as possible, the diffractometer upon which the data were collected lacks a camera with which to obtain a true representation of the crystal for a more explicit correction. The impact of this upon the data is further compounded by the crystal existing as a highly anisotropic plate. Errors carried through from this in the intensities will increase the residual levels in the model. This is in addition to the typical fourier rippling that is expected at heavy elements, and the impact of the spherical contraction of the valence shell owed to the ionisation of these metals in this highly charge system. Since these residuals exist close to these heavy metal centres and the model (and symmetry) has been thoroughly evaluated (see the manuscript), these are believed to have no physical significance.

PLAT971_ALERT_2_A Check Calcd Resid. Dens. 0.53Ang From Sr2 4.27 eA-3

Author Response: The sample is highly absorbing and contains a significant proportion of heavy elements. While efforts have been made to account for this as best as possible, the diffractometer upon which the data were collected lacks a camera with which to obtain a true representation of the crystal for a more explicit correction. The impact of this upon the data is further compounded by the crystal existing as a highly anisotropic plate. Errors carried through from this in the intensities will increase the residual levels in the model. This is in addition to the typical fourier rippling that is expected at heavy elements, and the impact of the spherical contraction of the valence shell owed to the ionisation of these metals in this highly charge system. Since these residuals exist close to these heavy metal centres and the model (and symmetry) has been thoroughly evaluated (see the manuscript), these are believed to have no physical significance.

PLAT971_ALERT_2_A Check Calcd Resid. Dens. 0.59Ang From Sr1 4.27 eA-3

Author Response: The sample is highly absorbing and contains a significant proportion of heavy elements. While efforts have been made to account for this as best as possible, the diffractometer upon which the data were collected lacks a camera with which to obtain a true representation of the crystal for a more explicit correction. The impact of this upon the data is further compounded by the crystal existing as a highly anisotropic plate. Errors carried through from this in the intensities will increase the residual levels in the model. This is in addition to the typical fourier rippling that is expected at heavy elements, and the impact of the spherical contraction of the valence shell owed to the ionisation of these metals in this highly charge system. Since these residuals exist close to these heavy metal centres and the model (and symmetry) has been thoroughly evaluated (see the manuscript), these are believed to have no physical significance.

PLAT971_ALERT_2_A Check Calcd Resid. Dens. 0.59Ang From Sr2 4.21 eA-3

Author Response: The sample is highly absorbing and contains a significant proportion of heavy elements. While efforts have been made to account for this as best as possible, the diffractometer upon which the data were collected lacks a camera with which to obtain a true representation of the crystal for a more explicit correction. The impact of this upon the data is further compounded by the crystal existing as a highly anisotropic plate. Errors carried through from this in the intensities will increase the residual levels in the model. This is in addition to the typical fourier rippling that is expected at heavy elements, and the impact of the spherical contraction of the valence shell owed to the ionisation of these metals in this highly charge system. Since these residuals exist close to these heavy metal centres and the model (and symmetry) has been thoroughly evaluated (see the manuscript), these are believed to have no physical significance.

```
PLAT971_ALERT_2_A Check Calcd Resid. Dens.  0.58Ang From Sr1            4.04 eA-3
```

Author Response: The sample is highly absorbing and contains a significant proportion of heavy elements. While efforts have been made to account for this as best as possible, the diffractometer upon which the data were collected lacks a camera with which to obtain a true representation of the crystal for a more explicit correction. The impact of this upon the data is further compounded by the crystal existing as a highly anisotropic plate. Errors carried through from this in the intensities will increase the residual levels in the model. This is in addition to the typical fourier rippling that is expected at heavy elements, and the impact of the spherical contraction of the valence shell owed to the ionisation of these metals in this highly charge system. Since these residuals exist close to these heavy metal centres and the model (and symmetry) has been thoroughly evaluated (see the manuscript), these are believed to have no physical significance.

```
PLAT971_ALERT_2_A Check Calcd Resid. Dens.  0.56Ang From Sr2            3.84 eA-3
```

Author Response: The sample is highly absorbing and contains a significant proportion of heavy elements. While efforts have been made to account for this as best as possible, the diffractometer upon which the data were collected lacks a camera with which to obtain a true representation of the crystal for a more explicit correction. The impact of this upon the data is further compounded by the crystal existing as a highly anisotropic plate. Errors carried through from this in the intensities will increase the residual levels in the model. This is in addition to the typical fourier rippling that is expected at heavy elements, and the impact of the spherical contraction of the valence shell owed to the ionisation of these metals in this highly charge system. Since these residuals exist close to these heavy metal centres and the model (and symmetry) has been thoroughly evaluated (see the manuscript), these are believed to have no physical significance.

```
PLAT971_ALERT_2_A Check Calcd Resid. Dens.  0.60Ang From Sr2            3.74 eA-3
```

Author Response: The sample is highly absorbing and contains a significant proportion of heavy elements. While efforts have been made to account for this as best as possible, the diffractometer upon which the data were collected lacks a camera with which to obtain a true representation of the crystal for a more explicit correction. The impact of this upon the data is further compounded by the crystal existing as a highly anisotropic plate. Errors carried through from this in the intensities will increase the residual levels in the model. This is in addition to the typical fourier rippling that is expected at heavy elements, and the impact of the spherical contraction of the valence shell owed to the ionisation of these metals in this highly charge system. Since these residuals exist close to these heavy metal centres and the model (and symmetry) has been thoroughly evaluated (see the manuscript), these are believed to have no physical significance.

Alert level B

```
PLAT097_ALERT_2_B Large Reported Max.  (Positive) Residual Density       6.04 eA-3
```

Author Response: The sample is highly absorbing and contains a significant proportion of heavy elements. While efforts have been made to account for this as best as possible, the diffractometer upon which the data were collected lacks a camera with which to obtain a true representation of the crystal for a more explicit correction. The impact of this upon the data is further compounded by the crystal existing as a highly anisotropic plate. Errors carried through from this in the intensities will increase the residual levels in the model. This is in addition to the typical fourier rippling that is expected at heavy elements, and the impact of the spherical contraction of the valence shell owed to the ionisation of these metals in this highly charge system. Since the residual exists close to a heavy metal centre and the model (and symmetry) has been thoroughly evaluated (see the manuscript), it is believed to have no physical significance.

```
PLAT196_ALERT_1_B No TEMP record and measurement temperature .NE.        293 Degree
```

Author Response: Model incorporates no riding hydrogens so no TEMP record is required.

```
PLAT971_ALERT_2_B Check Calcd Resid. Dens.  0.65Ang From Sr1            3.38 eA-3
```

Author Response: The sample is highly absorbing and contains a significant proportion of heavy elements. While efforts have been made to account for this as best as possible, the diffractometer upon which the data were collected lacks a camera with which to obtain a true representation of the crystal for a more explicit correction. The impact of this upon the data is further compounded by the crystal existing as a highly anisotropic plate. Errors carried through from this in the intensities will increase the residual levels in the model. This is in addition to the typical fourier rippling that is expected at heavy elements, and the impact of the spherical contraction of the valence shell owed to the ionisation of these metals in this highly charge system. Since these residuals exist close to these heavy metal centres and the model (and symmetry) has been thoroughly evaluated (see the manuscript), these are believed to have no physical significance.

```
PLAT971_ALERT_2_B Check Calcd Resid. Dens.  0.64Ang From Sr1            3.32 eA-3
```

Author Response: The sample is highly absorbing and contains a significant proportion of heavy elements. While efforts have been made to account for this as best as possible, the diffractometer upon which the data were collected lacks a camera with which to obtain a true representation of the crystal for a more explicit correction. The impact of this upon the data is further compounded by the crystal existing as a highly anisotropic plate. Errors carried through from this in the intensities will increase the residual levels in the model. This is in addition to the typical fourier rippling that is expected at heavy elements, and the impact of the spherical contraction of the valence shell owed to the ionisation of these metals in this highly charge system. Since these residuals exist close to these heavy metal centres and the model (and symmetry) has been thoroughly evaluated (see the manuscript), these are believed to have no physical significance.

```
PLAT971_ALERT_2_B Check Calcd Resid. Dens.  0.61Ang From Sr2            2.72 eA-3
```

Author Response: The sample is highly absorbing and contains a significant proportion of heavy elements. While efforts have been made to account for this as best as possible, the diffractometer upon which the data were collected lacks a camera with which to obtain a true representation of the crystal for a more explicit correction. The impact of this upon the data is further compounded by the crystal existing as a highly anisotropic plate. Errors carried through from this in the intensities will increase the residual levels in the model. This is in addition to the typical fourier rippling that is expected at heavy elements, and the impact of the spherical contraction of the valence shell owed to the ionisation of these metals in this highly charge system. Since these residuals exist close to these heavy metal centres and the model (and symmetry) has been thoroughly evaluated (see the manuscript), these are believed to have no physical significance.

```
PLAT972_ALERT_2_B Check Calcd Resid. Dens.  1.46Ang From Sr1           -2.96 eA-3
```

Author Response: The sample is highly absorbing and contains a significant proportion of heavy elements. While efforts have been made to account for this as best as possible, the diffractometer upon which the data were collected lacks a camera with which to obtain a true representation of the crystal for a more explicit correction. The impact of this upon the data is further compounded by the crystal existing as a highly anisotropic plate. Errors carried through from this in the intensities will increase the residual levels in the model. This is in addition to the typical fourier rippling that is expected at heavy elements, and the impact of the spherical contraction of the valence shell owed to the ionisation of these metals in this highly charge system. Since these residuals exist close to these heavy metal centres and the model (and symmetry) has been thoroughly evaluated (see the manuscript), these are believed to have no physical significance.

```
PLAT972_ALERT_2_B Check Calcd Resid. Dens.  1.77Ang From Sr2           -2.94 eA-3
```

Author Response: The sample is highly absorbing and contains a significant proportion of heavy elements. While efforts have been made to account for this as best as possible, the diffractometer upon which the data were collected lacks a camera with which to obtain a true representation of the crystal for a more explicit correction. The impact of this upon the data is further compounded by the crystal existing as a highly anisotropic plate. Errors carried through from this in the intensities will increase the residual levels in the model. This is in addition to the typical fourier rippling that is expected at heavy elements, and the impact of the spherical contraction of the valence shell owed to the ionisation of these metals in this highly charge system. Since these residuals exist close to these heavy metal centres and the model (and symmetry) has been thoroughly evaluated (see the manuscript), these are believed to have no physical significance.

```
PLAT972_ALERT_2_B Check Calcd Resid. Dens.  0.82Ang From O2            -2.75 eA-3
```

Author Response: The sample is highly absorbing and contains a significant proportion of heavy elements. While efforts have been made to account for this as best as possible, the diffractometer upon which the data were collected lacks a camera with which to obtain a true representation of the crystal for a more explicit correction. The impact of this upon the data is further compounded by the crystal existing as a highly anisotropic plate. Errors carried through from this in the intensities will increase the residual levels in the model. This is in addition to the typical fourier rippling that is expected at heavy elements, and the impact of the spherical contraction of the valence shell owed to the ionisation of these metals in this highly charge system. Since these residuals exist close to these heavy metal centres and the model (and symmetry) has been thoroughly evaluated (see the manuscript), these are believed to have no physical significance.

```
PLAT972_ALERT_2_B Check Calcd Resid. Dens.  1.23Ang From Sr2           -2.70 eA-3
```

Author Response: The sample is highly absorbing and contains a significant proportion of heavy elements. While efforts have been made to account for this as best as possible, the diffractometer upon which the data were collected lacks a camera with which to obtain a true representation of the crystal for a more explicit correction. The impact of this upon the data is further compounded by the crystal existing as a highly anisotropic plate. Errors carried through from this in the intensities will increase the residual levels in the model. This is in addition to the typical fourier rippling that is expected at heavy elements, and the impact of the spherical contraction of the valence shell owed to the ionisation of these metals in this highly charge system. Since these residuals exist close to these heavy metal centres and the model (and symmetry) has been thoroughly evaluated (see the manuscript), these are believed to have no physical significance.

```
PLAT975_ALERT_2_B Check Calcd Resid. Dens.  0.43Ang From O3      .       1.74 eA-3
```

Author Response: The sample is highly absorbing and contains a significant proportion of heavy elements. While efforts have been made to account for this as best as possible, the diffractometer upon which the data were collected lacks a camera with which to obtain a true representation of the crystal for a more explicit correction. The impact of this upon the data is further compounded by the crystal existing as a highly anisotropic plate. Errors carried through from this in the intensities will increase the residual levels in the model. This is in addition to the typical fourier rippling that is expected at heavy elements, and the impact of the spherical contraction of the valence shell owed to the ionisation of these metals in this highly charge system. Since these residuals exist close to these heavy metal centres and the model (and symmetry) has been thoroughly evaluated (see the manuscript), these are believed to have no physical significance.

```
PLAT975_ALERT_2_B Check Calcd Resid. Dens.  0.50Ang From O4      .       1.68 eA-3
```

Author Response: The sample is highly absorbing and contains a significant proportion of heavy elements. While efforts have been made to account for this as best as possible, the diffractometer upon which the data were collected lacks a camera with which to obtain a true representation of the crystal for a more explicit correction. The impact of this upon the data is further compounded by the crystal existing as a highly anisotropic plate. Errors carried through from this in the intensities will increase the residual levels in the model. This is in addition to the typical fourier rippling that is expected at heavy elements, and the impact of the spherical contraction of the valence shell owed to the ionisation of these metals in this highly charge system. Since these residuals exist close to these heavy metal centres and the model (and symmetry) has been thoroughly evaluated (see the manuscript), these are believed to have no physical significance.

```
PLAT975_ALERT_2_B Check Calcd Resid. Dens.  0.57Ang From O3      .       1.67 eA-3
```

Author Response: The sample is highly absorbing and contains a significant proportion of heavy elements. While efforts have been made to account for this as best as possible, the diffractometer upon which the data were collected lacks a camera with which to obtain a true representation of the crystal for a more explicit correction. The impact of this upon the data is further compounded by the crystal existing as a highly anisotropic plate. Errors carried through from this in the intensities will increase the residual levels in the model. This is in addition to the typical fourier rippling that is expected at heavy elements, and the impact of the spherical contraction of the valence shell owed to the ionisation of these metals in this highly charge system. Since these residuals exist close to these heavy metal centres and the model (and symmetry) has been thoroughly evaluated (see the manuscript), these are believed to have no physical significance.

Alert level C

```
DIFMX02_ALERT_1_C  The maximum difference density is > 0.1*ZMAX*0.75
            The relevant atom site should be identified.
PLAT041_ALERT_1_C Calc. and Reported SumFormula    Strings  Differ     Please Check
              Calc: O28 Sn8 Sr12
              Rep.: O7 Sn2 Sr3
PLAT042_ALERT_1_C Calc. and Reported MoietyFormula Strings  Differ     Please Check
              Calc: O28 Sn8, 12(Sr)
              Rep.: O7 Sn2 Sr3
PLAT911_ALERT_3_C Missing FCF Refl Between Thmin & STh/L=    0.600           2 Report
                2  0  0,   0  2  0,
PLAT971_ALERT_2_C Check Calcd Resid. Dens.  0.57Ang From O3              2.02 eA-3
```

Author Response: The sample is highly absorbing and contains a significant proportion of heavy elements. While efforts have been made to account for this as best as possible, the diffractometer upon which the data were collected lacks a camera with which to obtain a true representation of the crystal for a more explicit correction. The impact of this upon the data is further compounded by the crystal existing as a highly anisotropic plate. Errors carried through from this in the intensities will increase the residual levels in the model. This is in addition to the typical fourier rippling that is expected at heavy elements, and the impact of the spherical contraction of the valence shell owed to the ionisation of these metals in this highly charge system. Since these residuals exist close to these heavy metal centres and the model (and symmetry) has been thoroughly evaluated (see the manuscript), these are believed to have no physical significance.

```
PLAT971_ALERT_2_C Check Calcd Resid. Dens.  0.49Ang From O3             1.93 eA-3
```

Author Response: The sample is highly absorbing and contains a significant proportion of heavy elements. While efforts have been made to account for this as best as possible, the diffractometer upon which the data were collected lacks a camera with which to obtain a true representation of the crystal for a more explicit correction. The impact of this upon the data is further compounded by the crystal existing as a highly anisotropic plate. Errors carried through from this in the intensities will increase the residual levels in the model. This is in addition to the typical fourier rippling that is expected at heavy elements, and the impact of the spherical contraction of the valence shell owed to the ionisation of these metals in this highly charge system. Since these residuals exist close to these heavy metal centres and the model (and symmetry) has been thoroughly evaluated (see the manuscript), these are believed to have no physical significance.

```
PLAT971_ALERT_2_C Check Calcd Resid. Dens.  0.49Ang From O3             1.93 eA-3
```

Author Response: The sample is highly absorbing and contains a significant proportion of heavy elements. While efforts have been made to account for this as best as possible, the diffractometer upon which the data were collected lacks a camera with which to obtain a true representation of the crystal for a more explicit correction. The impact of this upon the data is further compounded by the crystal existing as a highly anisotropic plate. Errors carried through from this in the intensities will increase the residual levels in the model. This is in addition to the typical fourier rippling that is expected at heavy elements, and the impact of the spherical contraction of the valence shell owed to the ionisation of these metals in this highly charge system. Since these residuals exist close to these heavy metal centres and the model (and symmetry) has been thoroughly evaluated (see the manuscript), these are believed to have no physical significance.

PLAT971_ALERT_2_C Check Calcd Resid. Dens. 0.53Ang From O3   1.93 eA-3

Author Response: The sample is highly absorbing and contains a significant proportion of heavy elements. While efforts have been made to account for this as best as possible, the diffractometer upon which the data were collected lacks a camera with which to obtain a true representation of the crystal for a more explicit correction. The impact of this upon the data is further compounded by the crystal existing as a highly anisotropic plate. Errors carried through from this in the intensities will increase the residual levels in the model. This is in addition to the typical fourier rippling that is expected at heavy elements, and the impact of the spherical contraction of the valence shell owed to the ionisation of these metals in this highly charge system. Since these residuals exist close to these heavy metal centres and the model (and symmetry) has been thoroughly evaluated (see the manuscript), these are believed to have no physical significance.

PLAT971_ALERT_2_C Check Calcd Resid. Dens. 0.69Ang From O4   1.85 eA-3

Author Response: The sample is highly absorbing and contains a significant proportion of heavy elements. While efforts have been made to account for this as best as possible, the diffractometer upon which the data were collected lacks a camera with which to obtain a true representation of the crystal for a more explicit correction. The impact of this upon the data is further compounded by the crystal existing as a highly anisotropic plate. Errors carried through from this in the intensities will increase the residual levels in the model. This is in addition to the typical fourier rippling that is expected at heavy elements, and the impact of the spherical contraction of the valence shell owed to the ionisation of these metals in this highly charge system. Since these residuals exist close to these heavy metal centres and the model (and symmetry) has been thoroughly evaluated (see the manuscript), these are believed to have no physical significance.

PLAT971_ALERT_2_C Check Calcd Resid. Dens. 0.14Ang From O3   1.79 eA-3

Author Response: The sample is highly absorbing and contains a significant proportion of heavy elements. While efforts have been made to account for this as best as possible, the diffractometer upon which the data were collected lacks a camera with which to obtain a true representation of the crystal for a more explicit correction. The impact of this upon the data is further compounded by the crystal existing as a highly anisotropic plate. Errors carried through from this in the intensities will increase the residual levels in the model. This is in addition to the typical fourier rippling that is expected at heavy elements, and the impact of the spherical contraction of the valence shell owed to the ionisation of these metals in this highly charge system. Since these residuals exist close to these heavy metal centres and the model (and symmetry) has been thoroughly evaluated (see the manuscript), these are believed to have no physical significance.

```
PLAT971_ALERT_2_C Check Calcd Resid. Dens.  0.57Ang From O4             1.78 eA-3
```

Author Response: The sample is highly absorbing and contains a significant proportion of heavy elements. While efforts have been made to account for this as best as possible, the diffractometer upon which the data were collected lacks a camera with which to obtain a true representation of the crystal for a more explicit correction. The impact of this upon the data is further compounded by the crystal existing as a highly anisotropic plate. Errors carried through from this in the intensities will increase the residual levels in the model. This is in addition to the typical fourier rippling that is expected at heavy elements, and the impact of the spherical contraction of the valence shell owed to the ionisation of these metals in this highly charge system. Since these residuals exist close to these heavy metal centres and the model (and symmetry) has been thoroughly evaluated (see the manuscript), these are believed to have no physical significance.

```
PLAT971_ALERT_2_C Check Calcd Resid. Dens.  0.72Ang From O4             1.75 eA-3
```

Author Response: The sample is highly absorbing and contains a significant proportion of heavy elements. While efforts have been made to account for this as best as possible, the diffractometer upon which the data were collected lacks a camera with which to obtain a true representation of the crystal for a more explicit correction. The impact of this upon the data is further compounded by the crystal existing as a highly anisotropic plate. Errors carried through from this in the intensities will increase the residual levels in the model. This is in addition to the typical fourier rippling that is expected at heavy elements, and the impact of the spherical contraction of the valence shell owed to the ionisation of these metals in this highly charge system. Since these residuals exist close to these heavy metal centres and the model (and symmetry) has been thoroughly evaluated (see the manuscript), these are believed to have no physical significance.

```
PLAT971_ALERT_2_C Check Calcd Resid. Dens.  0.43Ang From O3             1.74 eA-3
```

Author Response: The sample is highly absorbing and contains a significant proportion of heavy elements. While efforts have been made to account for this as best as possible, the diffractometer upon which the data were collected lacks a camera with which to obtain a true representation of the crystal for a more explicit correction. The impact of this upon the data is further compounded by the crystal existing as a highly anisotropic plate. Errors carried through from this in the intensities will increase the residual levels in the model. This is in addition to the typical fourier rippling that is expected at heavy elements, and the impact of the spherical contraction of the valence shell owed to the ionisation of these metals in this highly charge system. Since these residuals exist close to these heavy metal centres and the model (and symmetry) has been thoroughly evaluated (see the manuscript), these are believed to have no physical significance.

```
PLAT971_ALERT_2_C Check Calcd Resid. Dens.  0.50Ang From O4             1.68 eA-3
```

Author Response: The sample is highly absorbing and contains a significant proportion of heavy elements. While efforts have been made to account for this as best as possible, the diffractometer upon which the data were collected lacks a camera with which to obtain a true representation of the crystal for a more explicit correction. The impact of this upon the data is further compounded by the crystal existing as a highly anisotropic plate. Errors carried through from this in the intensities will increase the residual levels in the model. This is in addition to the typical fourier rippling that is expected at heavy elements, and the impact of the spherical contraction of the valence shell owed to the ionisation of these metals in this highly charge system. Since these residuals exist close to these heavy metal centres and the model (and symmetry) has been thoroughly evaluated (see the manuscript), these are believed to have no physical significance.

```
PLAT971_ALERT_2_C Check Calcd Resid. Dens.  0.57Ang From O3             1.67 eA-3
```

Author Response: The sample is highly absorbing and contains a significant proportion of heavy elements. While efforts have been made to account for this as best as possible, the diffractometer upon which the data were collected lacks a camera with which to obtain a true representation of the crystal for a more explicit correction. The impact of this upon the data is further compounded by the crystal existing as a highly anisotropic plate. Errors carried through from this in the intensities will increase the residual levels in the model. This is in addition to the typical fourier rippling that is expected at heavy elements, and the impact of the spherical contraction of the valence shell owed to the ionisation of these metals in this highly charge system. Since these residuals exist close to these heavy metal centres and the model (and symmetry) has been thoroughly evaluated (see the manuscript), these are believed to have no physical significance.

```
PLAT971_ALERT_2_C Check Calcd Resid. Dens.  0.52Ang From O4             1.67 eA-3
```

Author Response: The sample is highly absorbing and contains a significant proportion of heavy elements. While efforts have been made to account for this as best as possible, the diffractometer upon which the data were collected lacks a camera with which to obtain a true representation of the crystal for a more explicit correction. The impact of this upon the data is further compounded by the crystal existing as a highly anisotropic plate. Errors carried through from this in the intensities will increase the residual levels in the model. This is in addition to the typical fourier rippling that is expected at heavy elements, and the impact of the spherical contraction of the valence shell owed to the ionisation of these metals in this highly charge system. Since these residuals exist close to these heavy metal centres and the model (and symmetry) has been thoroughly evaluated (see the manuscript), these are believed to have no physical significance.

```
PLAT972_ALERT_2_C Check Calcd Resid. Dens.  1.25Ang From Sr1           -2.50 eA-3
```

Author Response: The sample is highly absorbing and contains a significant proportion of heavy elements. While efforts have been made to account for this as best as possible, the diffractometer upon which the data were collected lacks a camera with which to obtain a true representation of the crystal for a more explicit correction. The impact of this upon the data is further compounded by the crystal existing as a highly anisotropic plate. Errors carried through from this in the intensities will increase the residual levels in the model. This is in addition to the typical fourier rippling that is expected at heavy elements, and the impact of the spherical contraction of the valence shell owed to the ionisation of these metals in this highly charge system. Since these residuals exist close to these heavy metal centres and the model (and symmetry) has been thoroughly evaluated (see the manuscript), these are believed to have no physical significance.

PLAT972_ALERT_2_C Check Calcd Resid. Dens. 1.24Ang From Sr1   -2.41 eA-3

Author Response: The sample is highly absorbing and contains a significant proportion of heavy elements. While efforts have been made to account for this as best as possible, the diffractometer upon which the data were collected lacks a camera with which to obtain a true representation of the crystal for a more explicit correction. The impact of this upon the data is further compounded by the crystal existing as a highly anisotropic plate. Errors carried through from this in the intensities will increase the residual levels in the model. This is in addition to the typical fourier rippling that is expected at heavy elements, and the impact of the spherical contraction of the valence shell owed to the ionisation of these metals in this highly charge system. Since these residuals exist close to these heavy metal centres and the model (and symmetry) has been thoroughly evaluated (see the manuscript), these are believed to have no physical significance.

PLAT972_ALERT_2_C Check Calcd Resid. Dens. 1.27Ang From Sr2   -2.28 eA-3

Author Response: The sample is highly absorbing and contains a significant proportion of heavy elements. While efforts have been made to account for this as best as possible, the diffractometer upon which the data were collected lacks a camera with which to obtain a true representation of the crystal for a more explicit correction. The impact of this upon the data is further compounded by the crystal existing as a highly anisotropic plate. Errors carried through from this in the intensities will increase the residual levels in the model. This is in addition to the typical fourier rippling that is expected at heavy elements, and the impact of the spherical contraction of the valence shell owed to the ionisation of these metals in this highly charge system. Since these residuals exist close to these heavy metal centres and the model (and symmetry) has been thoroughly evaluated (see the manuscript), these are believed to have no physical significance.

PLAT972_ALERT_2_C Check Calcd Resid. Dens. 1.18Ang From O1   -2.15 eA-3

Author Response: The sample is highly absorbing and contains a significant proportion of heavy elements. While efforts have been made to account for this as best as possible, the diffractometer upon which the data were collected lacks a camera with which to obtain a true representation of the crystal for a more explicit correction. The impact of this upon the data is further compounded by the crystal existing as a highly anisotropic plate. Errors carried through from this in the intensities will increase the residual levels in the model. This is in addition to the typical fourier rippling that is expected at heavy elements, and the impact of the spherical contraction of the valence shell owed to the ionisation of these metals in this highly charge system. Since these residuals exist close to these heavy metal centres and the model (and symmetry) has been thoroughly evaluated (see the manuscript), these are believed to have no physical significance.

```
PLAT972_ALERT_2_C Check Calcd Resid. Dens.  0.35Ang From O1            -2.02 eA-3
```

Author Response: The sample is highly absorbing and contains a significant proportion of heavy elements. While efforts have been made to account for this as best as possible, the diffractometer upon which the data were collected lacks a camera with which to obtain a true representation of the crystal for a more explicit correction. The impact of this upon the data is further compounded by the crystal existing as a highly anisotropic plate. Errors carried through from this in the intensities will increase the residual levels in the model. This is in addition to the typical fourier rippling that is expected at heavy elements, and the impact of the spherical contraction of the valence shell owed to the ionisation of these metals in this highly charge system. Since these residuals exist close to these heavy metal centres and the model (and symmetry) has been thoroughly evaluated (see the manuscript), these are believed to have no physical significance.

```
PLAT972_ALERT_2_C Check Calcd Resid. Dens.  1.10Ang From Sn1           -1.83 eA-3
```

Author Response: The sample is highly absorbing and contains a significant proportion of heavy elements. While efforts have been made to account for this as best as possible, the diffractometer upon which the data were collected lacks a camera with which to obtain a true representation of the crystal for a more explicit correction. The impact of this upon the data is further compounded by the crystal existing as a highly anisotropic plate. Errors carried through from this in the intensities will increase the residual levels in the model. This is in addition to the typical fourier rippling that is expected at heavy elements, and the impact of the spherical contraction of the valence shell owed to the ionisation of these metals in this highly charge system. Since these residuals exist close to these heavy metal centres and the model (and symmetry) has been thoroughly evaluated (see the manuscript), these are believed to have no physical significance.

PLAT972_ALERT_2_C Check Calcd Resid. Dens. 1.62Ang From O2 -1.82 eA-3

Author Response: The sample is highly absorbing and contains a significant proportion of heavy elements. While efforts have been made to account for this as best as possible, the diffractometer upon which the data were collected lacks a camera with which to obtain a true representation of the crystal for a more explicit correction. The impact of this upon the data is further compounded by the crystal existing as a highly anisotropic plate. Errors carried through from this in the intensities will increase the residual levels in the model. This is in addition to the typical fourier rippling that is expected at heavy elements, and the impact of the spherical contraction of the valence shell owed to the ionisation of these metals in this highly charge system. Since these residuals exist close to these heavy metal centres and the model (and symmetry) has been thoroughly evaluated (see the manuscript), these are believed to have no physical significance.

PLAT972_ALERT_2_C Check Calcd Resid. Dens. 0.47Ang From O2 -1.81 eA-3

Author Response: The sample is highly absorbing and contains a significant proportion of heavy elements. While efforts have been made to account for this as best as possible, the diffractometer upon which the data were collected lacks a camera with which to obtain a true representation of the crystal for a more explicit correction. The impact of this upon the data is further compounded by the crystal existing as a highly anisotropic plate. Errors carried through from this in the intensities will increase the residual levels in the model. This is in addition to the typical fourier rippling that is expected at heavy elements, and the impact of the spherical contraction of the valence shell owed to the ionisation of these metals in this highly charge system. Since these residuals exist close to these heavy metal centres and the model (and symmetry) has been thoroughly evaluated (see the manuscript), these are believed to have no physical significance.

PLAT972_ALERT_2_C Check Calcd Resid. Dens. 1.22Ang From O2 -1.79 eA-3

Author Response: The sample is highly absorbing and contains a significant proportion of heavy elements. While efforts have been made to account for this as best as possible, the diffractometer upon which the data were collected lacks a camera with which to obtain a true representation of the crystal for a more explicit correction. The impact of this upon the data is further compounded by the crystal existing as a highly anisotropic plate. Errors carried through from this in the intensities will increase the residual levels in the model. This is in addition to the typical fourier rippling that is expected at heavy elements, and the impact of the spherical contraction of the valence shell owed to the ionisation of these metals in this highly charge system. Since these residuals exist close to these heavy metal centres and the model (and symmetry) has been thoroughly evaluated (see the manuscript), these are believed to have no physical significance.

```
PLAT972_ALERT_2_C Check Calcd Resid. Dens.  1.05Ang From O2            -1.78 eA-3
```

Author Response: The sample is highly absorbing and contains a significant proportion of heavy elements. While efforts have been made to account for this as best as possible, the diffractometer upon which the data were collected lacks a camera with which to obtain a true representation of the crystal for a more explicit correction. The impact of this upon the data is further compounded by the crystal existing as a highly anisotropic plate. Errors carried through from this in the intensities will increase the residual levels in the model. This is in addition to the typical fourier rippling that is expected at heavy elements, and the impact of the spherical contraction of the valence shell owed to the ionisation of these metals in this highly charge system. Since these residuals exist close to these heavy metal centres and the model (and symmetry) has been thoroughly evaluated (see the manuscript), these are believed to have no physical significance.

```
PLAT972_ALERT_2_C Check Calcd Resid. Dens.  1.24Ang From O1            -1.75 eA-3
```

Author Response: The sample is highly absorbing and contains a significant proportion of heavy elements. While efforts have been made to account for this as best as possible, the diffractometer upon which the data were collected lacks a camera with which to obtain a true representation of the crystal for a more explicit correction. The impact of this upon the data is further compounded by the crystal existing as a highly anisotropic plate. Errors carried through from this in the intensities will increase the residual levels in the model. This is in addition to the typical fourier rippling that is expected at heavy elements, and the impact of the spherical contraction of the valence shell owed to the ionisation of these metals in this highly charge system. Since these residuals exist close to these heavy metal centres and the model (and symmetry) has been thoroughly evaluated (see the manuscript), these are believed to have no physical significance.

```
PLAT972_ALERT_2_C Check Calcd Resid. Dens.  1.18Ang From O1            -1.73 eA-3
```

Author Response: The sample is highly absorbing and contains a significant proportion of heavy elements. While efforts have been made to account for this as best as possible, the diffractometer upon which the data were collected lacks a camera with which to obtain a true representation of the crystal for a more explicit correction. The impact of this upon the data is further compounded by the crystal existing as a highly anisotropic plate. Errors carried through from this in the intensities will increase the residual levels in the model. This is in addition to the typical fourier rippling that is expected at heavy elements, and the impact of the spherical contraction of the valence shell owed to the ionisation of these metals in this highly charge system. Since these residuals exist close to these heavy metal centres and the model (and symmetry) has been thoroughly evaluated (see the manuscript), these are believed to have no physical significance.

```
PLAT972_ALERT_2_C Check Calcd Resid. Dens.  1.03Ang From O1            -1.68 eA-3
```

Author Response: The sample is highly absorbing and contains a significant proportion of heavy elements. While efforts have been made to account for this as best as possible, the diffractometer upon which the data were collected lacks a camera with which to obtain a true representation of the crystal for a more explicit correction. The impact of this upon the data is further compounded by the crystal existing as a highly anisotropic plate. Errors carried through from this in the intensities will increase the residual levels in the model. This is in addition to the typical fourier rippling that is expected at heavy elements, and the impact of the spherical contraction of the valence shell owed to the ionisation of these metals in this highly charge system. Since these residuals exist close to these heavy metal centres and the model (and symmetry) has been thoroughly evaluated (see the manuscript), these are believed to have no physical significance.

```
PLAT972_ALERT_2_C Check Calcd Resid. Dens.  1.23Ang From O2            -1.57 eA-3
```

Author Response: The sample is highly absorbing and contains a significant proportion of heavy elements. While efforts have been made to account for this as best as possible, the diffractometer upon which the data were collected lacks a camera with which to obtain a true representation of the crystal for a more explicit correction. The impact of this upon the data is further compounded by the crystal existing as a highly anisotropic plate. Errors carried through from this in the intensities will increase the residual levels in the model. This is in addition to the typical fourier rippling that is expected at heavy elements, and the impact of the spherical contraction of the valence shell owed to the ionisation of these metals in this highly charge system. Since these residuals exist close to these heavy metal centres and the model (and symmetry) has been thoroughly evaluated (see the manuscript), these are believed to have no physical significance.

PLAT972_ALERT_2_C Check Calcd Resid. Dens. 1.24Ang From O2 -1.51 eA-3

Author Response: The sample is highly absorbing and contains a significant proportion of heavy elements. While efforts have been made to account for this as best as possible, the diffractometer upon which the data were collected lacks a camera with which to obtain a true representation of the crystal for a more explicit correction. The impact of this upon the data is further compounded by the crystal existing as a highly anisotropic plate. Errors carried through from this in the intensities will increase the residual levels in the model. This is in addition to the typical fourier rippling that is expected at heavy elements, and the impact of the spherical contraction of the valence shell owed to the ionisation of these metals in this highly charge system. Since these residuals exist close to these heavy metal centres and the model (and symmetry) has been thoroughly evaluated (see the manuscript), these are believed to have no physical significance.

PLAT972_ALERT_2_C Check Calcd Resid. Dens. 1.56Ang From O3 -1.51 eA-3

Author Response: The sample is highly absorbing and contains a significant proportion of heavy elements. While efforts have been made to account for this as best as possible, the diffractometer upon which the data were collected lacks a camera with which to obtain a true representation of the crystal for a more explicit correction. The impact of this upon the data is further compounded by the crystal existing as a highly anisotropic plate. Errors carried through from this in the intensities will increase the residual levels in the model. This is in addition to the typical fourier rippling that is expected at heavy elements, and the impact of the spherical contraction of the valence shell owed to the ionisation of these metals in this highly charge system. Since these residuals exist close to these heavy metal centres and the model (and symmetry) has been thoroughly evaluated (see the manuscript), these are believed to have no physical significance.

Alert level G

```
ABSMU01_ALERT_1_G  Calculation of _exptl_absorpt_correction_mu
                 not performed for this radiation type.
PLAT004_ALERT_5_G Polymeric Structure Found with Maximum Dimension          2 Info
PLAT045_ALERT_1_G Calculated and Reported Z Differ by a Factor ...      0.250 Check
PLAT072_ALERT_2_G SHELXL First  Parameter in WGHT  Unusually Large       0.14 Report
PLAT111_ALERT_2_G ADDSYM Detects New (Pseudo) Centre of Symmetry .        100 %Fit
PLAT112_ALERT_2_G ADDSYM Detects New (Pseudo) Symm. Elem         m        100 %Fit
PLAT113_ALERT_2_G ADDSYM Suggests Possible Pseudo/New  Space Group       Cmcm Check
               Check Model Parameter Symmetry for Reflection Data Support
PLAT794_ALERT_5_G Tentative Bond Valency for Sn1       (IV)      .       4.14 Info
PLAT912_ALERT_4_G Missing # of FCF Reflections Above STh/L=  0.600         25 Note
PLAT916_ALERT_2_G Hooft y and Flack x Parameter Values Differ by .       0.47 Check
PLAT933_ALERT_2_G Number of HKL-OMIT Records in Embedded .res File          7 Note
                -2  0  0,  -1 -1  5,   0  0-10,   0  0 -4,   0  2  0,   1  1 -5,
                 2  0  0,
PLAT969_ALERT_5_G The 'Henn et al.' R-Factor-gap value ..........      5.730 Note
               Predicted wR2: Based on SigI**2  3.22 or SHELX Weight 16.35
```

---

11 **ALERT level A** = Most likely a serious problem - resolve or explain
12 **ALERT level B** = A potentially serious problem, consider carefully
32 **ALERT level C** = Check. Ensure it is not caused by an omission or oversight
12 **ALERT level G** = General information/check it is not something unexpected

6 ALERT type 1 CIF construction/syntax error, inconsistent or missing data
56 ALERT type 2 Indicator that the structure model may be wrong or deficient
1 ALERT type 3 Indicator that the structure quality may be low
1 ALERT type 4 Improvement, methodology, query or suggestion
3 ALERT type 5 Informative message, check

It is advisable to attempt to resolve as many as possible of the alerts in all categories. Often the minor alerts point to easily fixed oversights, errors and omissions in your CIF or refinement strategy, so attention to these fine details can be worthwhile. In order to resolve some of the more serious problems it may be necessary to carry out additional measurements or structure refinements. However, the purpose of your study may justify the reported deviations and the more serious of these should normally be commented upon in the discussion or experimental section of a paper or in the "special_details" fields of the CIF. checkCIF was carefully designed to identify outliers and unusual parameters, but every test has its limitations and alerts that are not important in a particular case may appear. Conversely, the absence of alerts does not guarantee there are no aspects of the results needing attention. It is up to the individual to critically assess their own results and, if necessary, seek expert advice.

Publication of your CIF in IUCr journals

A basic structural check has been run on your CIF. These basic checks will be run on all CIFs submitted for publication in IUCr journals (*Acta Crystallographica*, *Journal of Applied Crystallography*, *Journal of Synchrotron Radiation*); however, if you intend to submit to *Acta Crystallographica Section C* or *E* or *IUCrData*, you should make sure that full publication checks are run on the final version of your CIF prior to submission.

Publication of your CIF in other journals

Please refer to the *Notes for Authors* of the relevant journal for any special instructions relating to CIF submission.

PLATON version of 19/12/2024; check.def file version of 19/12/2024

Datablock srsno_i19vt_01_100_2_a21am - ellipsoid plot

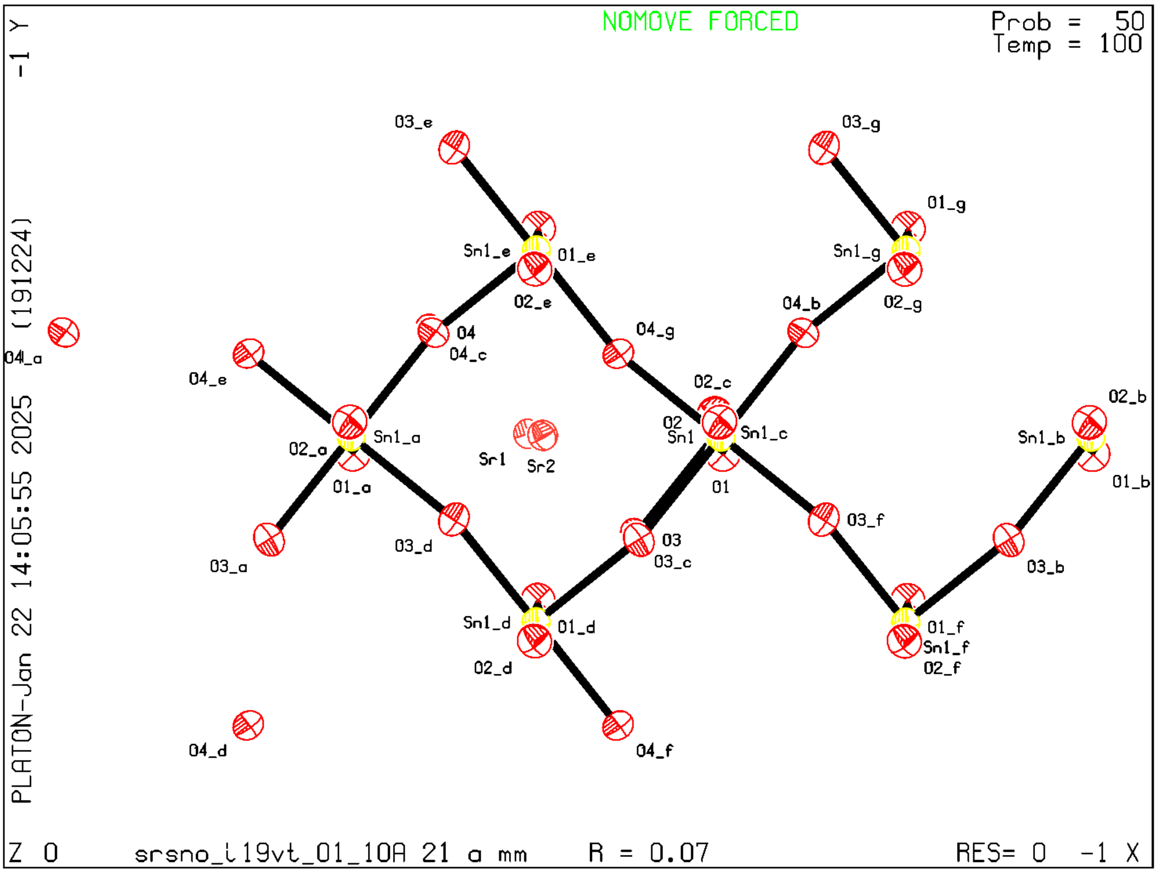

Supplement: Supplementary file 1 [file SrSnO_I19VT_01_100_2_a21am_checkcif.pdf › SSO_HP_VT_CIFs/SSO_VT_CIFS/SrSnO_I19VT_01_100_2_a21am_checkcif.pdf]
