# Supplementary material for: The varying temperature- and pressure-induced phase transition pathways in hybrid improper ferroelectric Sr3Sn2O7
Source: Acta Crystallogr B Struct Sci Cryst Eng Mater. 2025 Apr 14;81(Pt 3):318–24. doi: 10.1107/S2052520625002306 (PMC12147935; doi:10.1107/S2052520625002306)

checkCIF/PLATON report

Structure factors have been supplied for datablock(s) srsno_i19vt_15_420_2_pnab

THIS REPORT IS FOR GUIDANCE ONLY. IF USED AS PART OF A REVIEW PROCEDURE FOR PUBLICATION, IT SHOULD NOT REPLACE THE EXPERTISE OF AN EXPERIENCED CRYSTALLOGRAPHIC REFEREE.

No syntax errors found. CIF dictionary Interpreting this report

Datablock: srsno_i19vt_15_420_2_pnab

```
Bond precision:   Sn- O = 0.0035 A         Wavelength=0.68890

Cell:              a=5.7016(3)      b=5.7234(4)      c=20.6979(13)
                   alpha=90         beta=90          gamma=90
Temperature:       420 K
```

	Calculated	Reported
Volume	675.43(7)	675.42(7)
Space group	P n a b	P n a b
Hall group	-P 2bc 2n	-P 2bc 2n
Moiety formula	O28 Sn8, 12(Sr)	O7 Sn2 Sr3
Sum formula	O28 Sn8 Sr12	O7 Sn2 Sr3
Mr	2449.12	612.24
Dx,g cm-3	6.021	6.021
Z	1	4
Mu (mm-1)	28.070	28.415
F000	1080.0	1080.0
F000’	1061.72	
h,k,lmax	9,9,33	9,9,33
Nref	1534	1482
Tmin,Tmax		0.001,0.011
Tmin’		

```
Correction method= # Reported T Limits: Tmin=0.001 Tmax=0.011
AbsCorr = SPHERE

Data completeness= 0.966           Theta(max)= 34.153

R(reflections)= 0.0789( 1137)                      wR2(reflections)=
                                                    0.2223( 1482)
S = 1.032                  Npar= 56
```

The following ALERTS were generated. Each ALERT has the format
test-name_ALERT_alert-type_alert-level.
Click on the hyperlinks for more details of the test.

Alert level A

PLAT971_ALERT_2_A Check Calcd Resid. Dens. 0.71Ang From Sn1 5.48 eA-3

Author Response: The sample is highly absorbing and contains a significant proportion of heavy elements. While efforts have been made to account for this as best as possible, the diffractometer upon which the data were collected lacks a camera with which to obtain a true representation of the crystal for a more explicit correction. The impact of this upon the data is further compounded by the crystal existing as a highly anisotropic plate. Errors carried through from this in the intensities will increase the residual levels in the model. This is in addition to the typical fourier rippling that is expected at heavy elements, and the impact of the spherical contraction of the valence shell owed to the ionisation of these metals in this highly charge system. Since these residuals exist close to these heavy metal centres and the model (and symmetry) has been thoroughly evaluated (see the manuscript), these are believed to have no physical significance.

PLAT971_ALERT_2_A Check Calcd Resid. Dens. 0.72Ang From Sn1 5.48 eA-3

Author Response: The sample is highly absorbing and contains a significant proportion of heavy elements. While efforts have been made to account for this as best as possible, the diffractometer upon which the data were collected lacks a camera with which to obtain a true representation of the crystal for a more explicit correction. The impact of this upon the data is further compounded by the crystal existing as a highly anisotropic plate. Errors carried through from this in the intensities will increase the residual levels in the model. This is in addition to the typical fourier rippling that is expected at heavy elements, and the impact of the spherical contraction of the valence shell owed to the ionisation of these metals in this highly charge system. Since these residuals exist close to these heavy metal centres and the model (and symmetry) has been thoroughly evaluated (see the manuscript), these are believed to have no physical significance.

PLAT971_ALERT_2_A Check Calcd Resid. Dens. 0.72Ang From Sn1 5.42 eA-3

Author Response: The sample is highly absorbing and contains a significant proportion of heavy elements. While efforts have been made to account for this as best as possible, the diffractometer upon which the data were collected lacks a camera with which to obtain a true representation of the crystal for a more explicit correction. The impact of this upon the data is further compounded by the crystal existing as a highly anisotropic plate. Errors carried through from this in the intensities will increase the residual levels in the model. This is in addition to the typical fourier rippling that is expected at heavy elements, and the impact of the spherical contraction of the valence shell owed to the ionisation of these metals in this highly charge system. Since these residuals exist close to these heavy metal centres and the model (and symmetry) has been thoroughly evaluated (see the manuscript), these are believed to have no physical significance.

```
PLAT971_ALERT_2_A Check Calcd Resid. Dens.  0.65Ang From Sn1            5.42 eA-3
```

Author Response: The sample is highly absorbing and contains a significant proportion of heavy elements. While efforts have been made to account for this as best as possible, the diffractometer upon which the data were collected lacks a camera with which to obtain a true representation of the crystal for a more explicit correction. The impact of this upon the data is further compounded by the crystal existing as a highly anisotropic plate. Errors carried through from this in the intensities will increase the residual levels in the model. This is in addition to the typical fourier rippling that is expected at heavy elements, and the impact of the spherical contraction of the valence shell owed to the ionisation of these metals in this highly charge system. Since these residuals exist close to these heavy metal centres and the model (and symmetry) has been thoroughly evaluated (see the manuscript), these are believed to have no physical significance.

```
PLAT971_ALERT_2_A Check Calcd Resid. Dens.  0.70Ang From Sr2            3.69 eA-3
```

Author Response: The sample is highly absorbing and contains a significant proportion of heavy elements. While efforts have been made to account for this as best as possible, the diffractometer upon which the data were collected lacks a camera with which to obtain a true representation of the crystal for a more explicit correction. The impact of this upon the data is further compounded by the crystal existing as a highly anisotropic plate. Errors carried through from this in the intensities will increase the residual levels in the model. This is in addition to the typical fourier rippling that is expected at heavy elements, and the impact of the spherical contraction of the valence shell owed to the ionisation of these metals in this highly charge system. Since these residuals exist close to these heavy metal centres and the model (and symmetry) has been thoroughly evaluated (see the manuscript), these are believed to have no physical significance.

```
PLAT974_ALERT_2_A Check Calcd Negative Resid. Density on       Sr1      -2.54 eA-3
```

Author Response: The sample is highly absorbing and contains a significant proportion of heavy elements. While efforts have been made to account for this as best as possible, the diffractometer upon which the data were collected lacks a camera with which to obtain a true representation of the crystal for a more explicit correction. The impact of this upon the data is further compounded by the crystal existing as a highly anisotropic plate. Errors carried through from this in the intensities will increase the residual levels in the model. This is in addition to the typical fourier rippling that is expected at heavy elements, and the impact of the spherical contraction of the valence shell owed to the ionisation of these metals in this highly charge system. Since these residuals exist close to these heavy metal centres and the model (and symmetry) has been thoroughly evaluated (see the manuscript), these are believed to have no physical significance.

```
PLAT976_ALERT_2_A Check Calcd Resid. Dens.  0.71Ang From O2      .      -2.16 eA-3
```

Author Response: The sample is highly absorbing and contains a significant proportion of heavy elements. While efforts have been made to account for this as best as possible, the diffractometer upon which the data were collected lacks a camera with which to obtain a true representation of the crystal for a more explicit correction. The impact of this upon the data is further compounded by the crystal existing as a highly anisotropic plate. Errors carried through from this in the intensities will increase the residual levels in the model. This is in addition to the typical fourier rippling that is expected at heavy elements, and the impact of the spherical contraction of the valence shell owed to the ionisation of these metals in this highly charge system. Since these residuals exist close to these heavy metal centres and the model (and symmetry) has been thoroughly evaluated (see the manuscript), these are believed to have no physical significance.

Alert level B

```
PLAT097_ALERT_2_B Large Reported Max.  (Positive) Residual Density       5.74 eA-3
```

Author Response: The sample is highly absorbing and contains a significant proportion of heavy elements. While efforts have been made to account for this as best as possible, the diffractometer upon which the data were collected lacks a camera with which to obtain a true representation of the crystal for a more explicit correction. The impact of this upon the data is further compounded by the crystal existing as a highly anisotropic plate. Errors carried through from this in the intensities will increase the residual levels in the model. This is in addition to the typical fourier rippling that is expected at heavy elements, and the impact of the spherical contraction of the valence shell owed to the ionisation of these metals in this highly charge system. Since the residual exists close to a heavy metal centre and the model (and symmetry) has been thoroughly evaluated (see the manuscript), it is believed to have no physical significance.

```
PLAT112_ALERT_2_B ADDSYM Detects New (Pseudo) Symm. Elem         4        100 %Fit
```

Author Response: The deviations from the high symmetry aristotype are low, manifesting as low intensity superstructure reflections and small deviations from the tetragonal aristotype. The model (and symmetry) has been thoroughly evaluated (please see the manuscript).

```
PLAT112_ALERT_2_B ADDSYM Detects New (Pseudo) Symm. Elem         m        100 %Fit
```

Author Response: The deviations from the high symmetry aristotype are low, manifesting as low intensity superstructure reflections and small deviations from the tetragonal aristotype. The model (and symmetry) has been thoroughly evaluated (please see the manuscript).

```
PLAT112_ALERT_2_B ADDSYM Detects New (Pseudo) Symm. Elem         m        100 %Fit
```

Author Response: The deviations from the high symmetry aristotype are low, manifesting as low intensity superstructure reflections and small deviations from the tetragonal aristotype. The model (and symmetry) has been thoroughly evaluated (please see the manuscript).

```
PLAT113_ALERT_2_B ADDSYM Suggests Possible Pseudo/New  Space Group     I4/mmm Check
              Check Model Parameter Symmetry for Reflection Data Support
```

Author Response: The deviations from the high symmetry aristotype are low, manifesting as low intensity superstructure reflections and small deviations from the tetragonal aristotype. The model (and symmetry) has been thoroughly evaluated (please see the manuscript).

PLAT971_ALERT_2_B Check Calcd Resid. Dens. 0.71Ang From Sr2 3.32 eA-3

Author Response: The sample is highly absorbing and contains a significant proportion of heavy elements. While efforts have been made to account for this as best as possible, the diffractometer upon which the data were collected lacks a camera with which to obtain a true representation of the crystal for a more explicit correction. The impact of this upon the data is further compounded by the crystal existing as a highly anisotropic plate. Errors carried through from this in the intensities will increase the residual levels in the model. This is in addition to the typical fourier rippling that is expected at heavy elements, and the impact of the spherical contraction of the valence shell owed to the ionisation of these metals in this highly charge system. Since these residuals exist close to these heavy metal centres and the model (and symmetry) has been thoroughly evaluated (see the manuscript), these are believed to have no physical significance.

PLAT971_ALERT_2_B Check Calcd Resid. Dens. 0.60Ang From Sr2 3.27 eA-3

Author Response: The sample is highly absorbing and contains a significant proportion of heavy elements. While efforts have been made to account for this as best as possible, the diffractometer upon which the data were collected lacks a camera with which to obtain a true representation of the crystal for a more explicit correction. The impact of this upon the data is further compounded by the crystal existing as a highly anisotropic plate. Errors carried through from this in the intensities will increase the residual levels in the model. This is in addition to the typical fourier rippling that is expected at heavy elements, and the impact of the spherical contraction of the valence shell owed to the ionisation of these metals in this highly charge system. Since these residuals exist close to these heavy metal centres and the model (and symmetry) has been thoroughly evaluated (see the manuscript), these are believed to have no physical significance.

PLAT971_ALERT_2_B Check Calcd Resid. Dens. 0.71Ang From Sr1 3.19 eA-3

Author Response: The sample is highly absorbing and contains a significant proportion of heavy elements. While efforts have been made to account for this as best as possible, the diffractometer upon which the data were collected lacks a camera with which to obtain a true representation of the crystal for a more explicit correction. The impact of this upon the data is further compounded by the crystal existing as a highly anisotropic plate. Errors carried through from this in the intensities will increase the residual levels in the model. This is in addition to the typical fourier rippling that is expected at heavy elements, and the impact of the spherical contraction of the valence shell owed to the ionisation of these metals in this highly charge system. Since these residuals exist close to these heavy metal centres and the model (and symmetry) has been thoroughly evaluated (see the manuscript), these are believed to have no physical significance.

```
PLAT971_ALERT_2_B Check Calcd Resid. Dens.  0.75Ang From Sr2            3.16 eA-3
```

Author Response: The sample is highly absorbing and contains a significant proportion of heavy elements. While efforts have been made to account for this as best as possible, the diffractometer upon which the data were collected lacks a camera with which to obtain a true representation of the crystal for a more explicit correction. The impact of this upon the data is further compounded by the crystal existing as a highly anisotropic plate. Errors carried through from this in the intensities will increase the residual levels in the model. This is in addition to the typical fourier rippling that is expected at heavy elements, and the impact of the spherical contraction of the valence shell owed to the ionisation of these metals in this highly charge system. Since these residuals exist close to these heavy metal centres and the model (and symmetry) has been thoroughly evaluated (see the manuscript), these are believed to have no physical significance.

```
PLAT971_ALERT_2_B Check Calcd Resid. Dens.  0.72Ang From Sr1            2.96 eA-3
```

Author Response: The sample is highly absorbing and contains a significant proportion of heavy elements. While efforts have been made to account for this as best as possible, the diffractometer upon which the data were collected lacks a camera with which to obtain a true representation of the crystal for a more explicit correction. The impact of this upon the data is further compounded by the crystal existing as a highly anisotropic plate. Errors carried through from this in the intensities will increase the residual levels in the model. This is in addition to the typical fourier rippling that is expected at heavy elements, and the impact of the spherical contraction of the valence shell owed to the ionisation of these metals in this highly charge system. Since these residuals exist close to these heavy metal centres and the model (and symmetry) has been thoroughly evaluated (see the manuscript), these are believed to have no physical significance.

```
PLAT971_ALERT_2_B Check Calcd Resid. Dens.  0.71Ang From Sr1            2.76 eA-3
```

Author Response: The sample is highly absorbing and contains a significant proportion of heavy elements. While efforts have been made to account for this as best as possible, the diffractometer upon which the data were collected lacks a camera with which to obtain a true representation of the crystal for a more explicit correction. The impact of this upon the data is further compounded by the crystal existing as a highly anisotropic plate. Errors carried through from this in the intensities will increase the residual levels in the model. This is in addition to the typical fourier rippling that is expected at heavy elements, and the impact of the spherical contraction of the valence shell owed to the ionisation of these metals in this highly charge system. Since these residuals exist close to these heavy metal centres and the model (and symmetry) has been thoroughly evaluated (see the manuscript), these are believed to have no physical significance.

```
PLAT972_ALERT_2_B Check Calcd Resid. Dens.  0.35Ang From Sr2           -3.24 eA-3
```

Author Response: The sample is highly absorbing and contains a significant proportion of heavy elements. While efforts have been made to account for this as best as possible, the diffractometer upon which the data were collected lacks a camera with which to obtain a true representation of the crystal for a more explicit correction. The impact of this upon the data is further compounded by the crystal existing as a highly anisotropic plate. Errors carried through from this in the intensities will increase the residual levels in the model. This is in addition to the typical fourier rippling that is expected at heavy elements, and the impact of the spherical contraction of the valence shell owed to the ionisation of these metals in this highly charge system. Since these residuals exist close to these heavy metal centres and the model (and symmetry) has been thoroughly evaluated (see the manuscript), these are believed to have no physical significance.

```
PLAT972_ALERT_2_B Check Calcd Resid. Dens.  1.60Ang From Sr1           -2.73 eA-3
```

Author Response: The sample is highly absorbing and contains a significant proportion of heavy elements. While efforts have been made to account for this as best as possible, the diffractometer upon which the data were collected lacks a camera with which to obtain a true representation of the crystal for a more explicit correction. The impact of this upon the data is further compounded by the crystal existing as a highly anisotropic plate. Errors carried through from this in the intensities will increase the residual levels in the model. This is in addition to the typical fourier rippling that is expected at heavy elements, and the impact of the spherical contraction of the valence shell owed to the ionisation of these metals in this highly charge system. Since these residuals exist close to these heavy metal centres and the model (and symmetry) has been thoroughly evaluated (see the manuscript), these are believed to have no physical significance.

```
PLAT975_ALERT_2_B Check Calcd Resid. Dens.  0.54Ang From O4      .       1.62 eA-3
```

Author Response: The sample is highly absorbing and contains a significant proportion of heavy elements. While efforts have been made to account for this as best as possible, the diffractometer upon which the data were collected lacks a camera with which to obtain a true representation of the crystal for a more explicit correction. The impact of this upon the data is further compounded by the crystal existing as a highly anisotropic plate. Errors carried through from this in the intensities will increase the residual levels in the model. This is in addition to the typical fourier rippling that is expected at heavy elements, and the impact of the spherical contraction of the valence shell owed to the ionisation of these metals in this highly charge system. Since these residuals exist close to these heavy metal centres and the model (and symmetry) has been thoroughly evaluated (see the manuscript), these are believed to have no physical significance.

```
PLAT975_ALERT_2_B Check Calcd Resid. Dens.  0.60Ang From O3      .       1.58 eA-3
```

Author Response: The sample is highly absorbing and contains a significant proportion of heavy elements. While efforts have been made to account for this as best as possible, the diffractometer upon which the data were collected lacks a camera with which to obtain a true representation of the crystal for a more explicit correction. The impact of this upon the data is further compounded by the crystal existing as a highly anisotropic plate. Errors carried through from this in the intensities will increase the residual levels in the model. This is in addition to the typical fourier rippling that is expected at heavy elements, and the impact of the spherical contraction of the valence shell owed to the ionisation of these metals in this highly charge system. Since these residuals exist close to these heavy metal centres and the model (and symmetry) has been thoroughly evaluated (see the manuscript), these are believed to have no physical significance.

Alert level C

```
DIFMX02_ALERT_1_C  The maximum difference density is > 0.1*ZMAX*0.75
            The relevant atom site should be identified.
PLAT041_ALERT_1_C Calc. and Reported SumFormula    Strings  Differ     Please Check
              Calc: O28 Sn8 Sr12
              Rep.: O7 Sn2 Sr3
PLAT042_ALERT_1_C Calc. and Reported MoietyFormula Strings  Differ     Please Check
              Calc: O28 Sn8, 12(Sr)
              Rep.: O7 Sn2 Sr3
PLAT051_ALERT_1_C Mu(calc) and Mu(cif) Ratio Differs from 1.0 by .       1.22 %
PLAT911_ALERT_3_C Missing FCF Refl Between Thmin & STh/L=    0.600          2 Report
                2  0  0,   0  2  0,
PLAT971_ALERT_2_C Check Calcd Resid. Dens.  0.73Ang From Sr1             2.48 eA-3
```

Author Response: The sample is highly absorbing and contains a significant proportion of heavy elements. While efforts have been made to account for this as best as possible, the diffractometer upon which the data were collected lacks a camera with which to obtain a true representation of the crystal for a more explicit correction. The impact of this upon the data is further compounded by the crystal existing as a highly anisotropic plate. Errors carried through from this in the intensities will increase the residual levels in the model. This is in addition to the typical fourier rippling that is expected at heavy elements, and the impact of the spherical contraction of the valence shell owed to the ionisation of these metals in this highly charge system. Since these residuals exist close to these heavy metal centres and the model (and symmetry) has been thoroughly evaluated (see the manuscript), these are believed to have no physical significance.

```
PLAT971_ALERT_2_C Check Calcd Resid. Dens.  0.14Ang From O4              1.74 eA-3
```

Author Response: The sample is highly absorbing and contains a significant proportion of heavy elements. While efforts have been made to account for this as best as possible, the diffractometer upon which the data were collected lacks a camera with which to obtain a true representation of the crystal for a more explicit correction. The impact of this upon the data is further compounded by the crystal existing as a highly anisotropic plate. Errors carried through from this in the intensities will increase the residual levels in the model. This is in addition to the typical fourier rippling that is expected at heavy elements, and the impact of the spherical contraction of the valence shell owed to the ionisation of these metals in this highly charge system. Since these residuals exist close to these heavy metal centres and the model (and symmetry) has been thoroughly evaluated (see the manuscript), these are believed to have no physical significance.

PLAT971_ALERT_2_C Check Calcd Resid. Dens. 0.71Ang From O4 1.66 eA-3

Author Response: The sample is highly absorbing and contains a significant proportion of heavy elements. While efforts have been made to account for this as best as possible, the diffractometer upon which the data were collected lacks a camera with which to obtain a true representation of the crystal for a more explicit correction. The impact of this upon the data is further compounded by the crystal existing as a highly anisotropic plate. Errors carried through from this in the intensities will increase the residual levels in the model. This is in addition to the typical fourier rippling that is expected at heavy elements, and the impact of the spherical contraction of the valence shell owed to the ionisation of these metals in this highly charge system. Since these residuals exist close to these heavy metal centres and the model (and symmetry) has been thoroughly evaluated (see the manuscript), these are believed to have no physical significance.

PLAT971_ALERT_2_C Check Calcd Resid. Dens. 0.83Ang From O3 1.65 eA-3

Author Response: The sample is highly absorbing and contains a significant proportion of heavy elements. While efforts have been made to account for this as best as possible, the diffractometer upon which the data were collected lacks a camera with which to obtain a true representation of the crystal for a more explicit correction. The impact of this upon the data is further compounded by the crystal existing as a highly anisotropic plate. Errors carried through from this in the intensities will increase the residual levels in the model. This is in addition to the typical fourier rippling that is expected at heavy elements, and the impact of the spherical contraction of the valence shell owed to the ionisation of these metals in this highly charge system. Since these residuals exist close to these heavy metal centres and the model (and symmetry) has been thoroughly evaluated (see the manuscript), these are believed to have no physical significance.

PLAT971_ALERT_2_C Check Calcd Resid. Dens. 0.32Ang From O3 1.64 eA-3

Author Response: The sample is highly absorbing and contains a significant proportion of heavy elements. While efforts have been made to account for this as best as possible, the diffractometer upon which the data were collected lacks a camera with which to obtain a true representation of the crystal for a more explicit correction. The impact of this upon the data is further compounded by the crystal existing as a highly anisotropic plate. Errors carried through from this in the intensities will increase the residual levels in the model. This is in addition to the typical fourier rippling that is expected at heavy elements, and the impact of the spherical contraction of the valence shell owed to the ionisation of these metals in this highly charge system. Since these residuals exist close to these heavy metal centres and the model (and symmetry) has been thoroughly evaluated (see the manuscript), these are believed to have no physical significance.

```
PLAT971_ALERT_2_C Check Calcd Resid. Dens.  0.72Ang From O4             1.64 eA-3
```

Author Response: The sample is highly absorbing and contains a significant proportion of heavy elements. While efforts have been made to account for this as best as possible, the diffractometer upon which the data were collected lacks a camera with which to obtain a true representation of the crystal for a more explicit correction. The impact of this upon the data is further compounded by the crystal existing as a highly anisotropic plate. Errors carried through from this in the intensities will increase the residual levels in the model. This is in addition to the typical fourier rippling that is expected at heavy elements, and the impact of the spherical contraction of the valence shell owed to the ionisation of these metals in this highly charge system. Since these residuals exist close to these heavy metal centres and the model (and symmetry) has been thoroughly evaluated (see the manuscript), these are believed to have no physical significance.

```
PLAT971_ALERT_2_C Check Calcd Resid. Dens.  0.54Ang From O4             1.62 eA-3
```

Author Response: The sample is highly absorbing and contains a significant proportion of heavy elements. While efforts have been made to account for this as best as possible, the diffractometer upon which the data were collected lacks a camera with which to obtain a true representation of the crystal for a more explicit correction. The impact of this upon the data is further compounded by the crystal existing as a highly anisotropic plate. Errors carried through from this in the intensities will increase the residual levels in the model. This is in addition to the typical fourier rippling that is expected at heavy elements, and the impact of the spherical contraction of the valence shell owed to the ionisation of these metals in this highly charge system. Since these residuals exist close to these heavy metal centres and the model (and symmetry) has been thoroughly evaluated (see the manuscript), these are believed to have no physical significance.

```
PLAT971_ALERT_2_C Check Calcd Resid. Dens.  0.60Ang From O3           1.58 eA-3
```

Author Response: The sample is highly absorbing and contains a significant proportion of heavy elements. While efforts have been made to account for this as best as possible, the diffractometer upon which the data were collected lacks a camera with which to obtain a true representation of the crystal for a more explicit correction. The impact of this upon the data is further compounded by the crystal existing as a highly anisotropic plate. Errors carried through from this in the intensities will increase the residual levels in the model. This is in addition to the typical fourier rippling that is expected at heavy elements, and the impact of the spherical contraction of the valence shell owed to the ionisation of these metals in this highly charge system. Since these residuals exist close to these heavy metal centres and the model (and symmetry) has been thoroughly evaluated (see the manuscript), these are believed to have no physical significance.

```
PLAT971_ALERT_2_C Check Calcd Resid. Dens.  0.66Ang From O3           1.56 eA-3
```

Author Response: The sample is highly absorbing and contains a significant proportion of heavy elements. While efforts have been made to account for this as best as possible, the diffractometer upon which the data were collected lacks a camera with which to obtain a true representation of the crystal for a more explicit correction. The impact of this upon the data is further compounded by the crystal existing as a highly anisotropic plate. Errors carried through from this in the intensities will increase the residual levels in the model. This is in addition to the typical fourier rippling that is expected at heavy elements, and the impact of the spherical contraction of the valence shell owed to the ionisation of these metals in this highly charge system. Since these residuals exist close to these heavy metal centres and the model (and symmetry) has been thoroughly evaluated (see the manuscript), these are believed to have no physical significance.

```
PLAT971_ALERT_2_C Check Calcd Resid. Dens.  0.68Ang From O3           1.53 eA-3
```

Author Response: The sample is highly absorbing and contains a significant proportion of heavy elements. While efforts have been made to account for this as best as possible, the diffractometer upon which the data were collected lacks a camera with which to obtain a true representation of the crystal for a more explicit correction. The impact of this upon the data is further compounded by the crystal existing as a highly anisotropic plate. Errors carried through from this in the intensities will increase the residual levels in the model. This is in addition to the typical fourier rippling that is expected at heavy elements, and the impact of the spherical contraction of the valence shell owed to the ionisation of these metals in this highly charge system. Since these residuals exist close to these heavy metal centres and the model (and symmetry) has been thoroughly evaluated (see the manuscript), these are believed to have no physical significance.

```
PLAT972_ALERT_2_C Check Calcd Resid. Dens.  1.23Ang From Sr1          -2.43 eA-3
```

Author Response: The sample is highly absorbing and contains a significant proportion of heavy elements. While efforts have been made to account for this as best as possible, the diffractometer upon which the data were collected lacks a camera with which to obtain a true representation of the crystal for a more explicit correction. The impact of this upon the data is further compounded by the crystal existing as a highly anisotropic plate. Errors carried through from this in the intensities will increase the residual levels in the model. This is in addition to the typical fourier rippling that is expected at heavy elements, and the impact of the spherical contraction of the valence shell owed to the ionisation of these metals in this highly charge system. Since these residuals exist close to these heavy metal centres and the model (and symmetry) has been thoroughly evaluated (see the manuscript), these are believed to have no physical significance.

```
PLAT972_ALERT_2_C Check Calcd Resid. Dens.  1.24Ang From Sr1          -2.33 eA-3
```

Author Response: The sample is highly absorbing and contains a significant proportion of heavy elements. While efforts have been made to account for this as best as possible, the diffractometer upon which the data were collected lacks a camera with which to obtain a true representation of the crystal for a more explicit correction. The impact of this upon the data is further compounded by the crystal existing as a highly anisotropic plate. Errors carried through from this in the intensities will increase the residual levels in the model. This is in addition to the typical fourier rippling that is expected at heavy elements, and the impact of the spherical contraction of the valence shell owed to the ionisation of these metals in this highly charge system. Since these residuals exist close to these heavy metal centres and the model (and symmetry) has been thoroughly evaluated (see the manuscript), these are believed to have no physical significance.

PLAT972_ALERT_2_C Check Calcd Resid. Dens. 0.71Ang From O2 -2.16 eA-3

Author Response: The sample is highly absorbing and contains a significant proportion of heavy elements. While efforts have been made to account for this as best as possible, the diffractometer upon which the data were collected lacks a camera with which to obtain a true representation of the crystal for a more explicit correction. The impact of this upon the data is further compounded by the crystal existing as a highly anisotropic plate. Errors carried through from this in the intensities will increase the residual levels in the model. This is in addition to the typical fourier rippling that is expected at heavy elements, and the impact of the spherical contraction of the valence shell owed to the ionisation of these metals in this highly charge system. Since these residuals exist close to these heavy metal centres and the model (and symmetry) has been thoroughly evaluated (see the manuscript), these are believed to have no physical significance.

PLAT972_ALERT_2_C Check Calcd Resid. Dens. 1.07Ang From O1 -2.14 eA-3

Author Response: The sample is highly absorbing and contains a significant proportion of heavy elements. While efforts have been made to account for this as best as possible, the diffractometer upon which the data were collected lacks a camera with which to obtain a true representation of the crystal for a more explicit correction. The impact of this upon the data is further compounded by the crystal existing as a highly anisotropic plate. Errors carried through from this in the intensities will increase the residual levels in the model. This is in addition to the typical fourier rippling that is expected at heavy elements, and the impact of the spherical contraction of the valence shell owed to the ionisation of these metals in this highly charge system. Since these residuals exist close to these heavy metal centres and the model (and symmetry) has been thoroughly evaluated (see the manuscript), these are believed to have no physical significance.

PLAT972_ALERT_2_C Check Calcd Resid. Dens. 0.37Ang From O1 -2.10 eA-3

Author Response: The sample is highly absorbing and contains a significant proportion of heavy elements. While efforts have been made to account for this as best as possible, the diffractometer upon which the data were collected lacks a camera with which to obtain a true representation of the crystal for a more explicit correction. The impact of this upon the data is further compounded by the crystal existing as a highly anisotropic plate. Errors carried through from this in the intensities will increase the residual levels in the model. This is in addition to the typical fourier rippling that is expected at heavy elements, and the impact of the spherical contraction of the valence shell owed to the ionisation of these metals in this highly charge system. Since these residuals exist close to these heavy metal centres and the model (and symmetry) has been thoroughly evaluated (see the manuscript), these are believed to have no physical significance.

`PLAT972_ALERT_2_C Check Calcd Resid. Dens.  1.25Ang From Sr2           -1.93 eA-3`

Author Response: The sample is highly absorbing and contains a significant proportion of heavy elements. While efforts have been made to account for this as best as possible, the diffractometer upon which the data were collected lacks a camera with which to obtain a true representation of the crystal for a more explicit correction. The impact of this upon the data is further compounded by the crystal existing as a highly anisotropic plate. Errors carried through from this in the intensities will increase the residual levels in the model. This is in addition to the typical fourier rippling that is expected at heavy elements, and the impact of the spherical contraction of the valence shell owed to the ionisation of these metals in this highly charge system. Since these residuals exist close to these heavy metal centres and the model (and symmetry) has been thoroughly evaluated (see the manuscript), these are believed to have no physical significance.

`PLAT972_ALERT_2_C Check Calcd Resid. Dens.  1.24Ang From Sr2           -1.91 eA-3`

Author Response: The sample is highly absorbing and contains a significant proportion of heavy elements. While efforts have been made to account for this as best as possible, the diffractometer upon which the data were collected lacks a camera with which to obtain a true representation of the crystal for a more explicit correction. The impact of this upon the data is further compounded by the crystal existing as a highly anisotropic plate. Errors carried through from this in the intensities will increase the residual levels in the model. This is in addition to the typical fourier rippling that is expected at heavy elements, and the impact of the spherical contraction of the valence shell owed to the ionisation of these metals in this highly charge system. Since these residuals exist close to these heavy metal centres and the model (and symmetry) has been thoroughly evaluated (see the manuscript), these are believed to have no physical significance.

PLAT972_ALERT_2_C Check Calcd Resid. Dens. 1.22Ang From Sr2   -1.89 eA-3

Author Response: The sample is highly absorbing and contains a significant proportion of heavy elements. While efforts have been made to account for this as best as possible, the diffractometer upon which the data were collected lacks a camera with which to obtain a true representation of the crystal for a more explicit correction. The impact of this upon the data is further compounded by the crystal existing as a highly anisotropic plate. Errors carried through from this in the intensities will increase the residual levels in the model. This is in addition to the typical fourier rippling that is expected at heavy elements, and the impact of the spherical contraction of the valence shell owed to the ionisation of these metals in this highly charge system. Since these residuals exist close to these heavy metal centres and the model (and symmetry) has been thoroughly evaluated (see the manuscript), these are believed to have no physical significance.

PLAT972_ALERT_2_C Check Calcd Resid. Dens. 0.83Ang From O1   -1.87 eA-3

Author Response: The sample is highly absorbing and contains a significant proportion of heavy elements. While efforts have been made to account for this as best as possible, the diffractometer upon which the data were collected lacks a camera with which to obtain a true representation of the crystal for a more explicit correction. The impact of this upon the data is further compounded by the crystal existing as a highly anisotropic plate. Errors carried through from this in the intensities will increase the residual levels in the model. This is in addition to the typical fourier rippling that is expected at heavy elements, and the impact of the spherical contraction of the valence shell owed to the ionisation of these metals in this highly charge system. Since these residuals exist close to these heavy metal centres and the model (and symmetry) has been thoroughly evaluated (see the manuscript), these are believed to have no physical significance.

PLAT972_ALERT_2_C Check Calcd Resid. Dens. 1.27Ang From Sr2   -1.84 eA-3

Author Response: The sample is highly absorbing and contains a significant proportion of heavy elements. While efforts have been made to account for this as best as possible, the diffractometer upon which the data were collected lacks a camera with which to obtain a true representation of the crystal for a more explicit correction. The impact of this upon the data is further compounded by the crystal existing as a highly anisotropic plate. Errors carried through from this in the intensities will increase the residual levels in the model. This is in addition to the typical fourier rippling that is expected at heavy elements, and the impact of the spherical contraction of the valence shell owed to the ionisation of these metals in this highly charge system. Since these residuals exist close to these heavy metal centres and the model (and symmetry) has been thoroughly evaluated (see the manuscript), these are believed to have no physical significance.

```
PLAT972_ALERT_2_C Check Calcd Resid. Dens.  0.37Ang From O2            -1.84 eA-3
```

Author Response: The sample is highly absorbing and contains a significant proportion of heavy elements. While efforts have been made to account for this as best as possible, the diffractometer upon which the data were collected lacks a camera with which to obtain a true representation of the crystal for a more explicit correction. The impact of this upon the data is further compounded by the crystal existing as a highly anisotropic plate. Errors carried through from this in the intensities will increase the residual levels in the model. This is in addition to the typical fourier rippling that is expected at heavy elements, and the impact of the spherical contraction of the valence shell owed to the ionisation of these metals in this highly charge system. Since these residuals exist close to these heavy metal centres and the model (and symmetry) has been thoroughly evaluated (see the manuscript), these are believed to have no physical significance.

```
PLAT972_ALERT_2_C Check Calcd Resid. Dens.  1.25Ang From O2            -1.82 eA-3
```

Author Response: The sample is highly absorbing and contains a significant proportion of heavy elements. While efforts have been made to account for this as best as possible, the diffractometer upon which the data were collected lacks a camera with which to obtain a true representation of the crystal for a more explicit correction. The impact of this upon the data is further compounded by the crystal existing as a highly anisotropic plate. Errors carried through from this in the intensities will increase the residual levels in the model. This is in addition to the typical fourier rippling that is expected at heavy elements, and the impact of the spherical contraction of the valence shell owed to the ionisation of these metals in this highly charge system. Since these residuals exist close to these heavy metal centres and the model (and symmetry) has been thoroughly evaluated (see the manuscript), these are believed to have no physical significance.

```
PLAT972_ALERT_2_C Check Calcd Resid. Dens.  1.27Ang From Sn1           -1.74 eA-3
```

Author Response: The sample is highly absorbing and contains a significant proportion of heavy elements. While efforts have been made to account for this as best as possible, the diffractometer upon which the data were collected lacks a camera with which to obtain a true representation of the crystal for a more explicit correction. The impact of this upon the data is further compounded by the crystal existing as a highly anisotropic plate. Errors carried through from this in the intensities will increase the residual levels in the model. This is in addition to the typical fourier rippling that is expected at heavy elements, and the impact of the spherical contraction of the valence shell owed to the ionisation of these metals in this highly charge system. Since these residuals exist close to these heavy metal centres and the model (and symmetry) has been thoroughly evaluated (see the manuscript), these are believed to have no physical significance.

```
PLAT972_ALERT_2_C Check Calcd Resid. Dens.  1.28Ang From Sr1           -1.73 eA-3
```

Author Response: The sample is highly absorbing and contains a significant proportion of heavy elements. While efforts have been made to account for this as best as possible, the diffractometer upon which the data were collected lacks a camera with which to obtain a true representation of the crystal for a more explicit correction. The impact of this upon the data is further compounded by the crystal existing as a highly anisotropic plate. Errors carried through from this in the intensities will increase the residual levels in the model. This is in addition to the typical fourier rippling that is expected at heavy elements, and the impact of the spherical contraction of the valence shell owed to the ionisation of these metals in this highly charge system. Since these residuals exist close to these heavy metal centres and the model (and symmetry) has been thoroughly evaluated (see the manuscript), these are believed to have no physical significance.

```
PLAT972_ALERT_2_C Check Calcd Resid. Dens.  1.05Ang From O2            -1.69 eA-3
```

Author Response: The sample is highly absorbing and contains a significant proportion of heavy elements. While efforts have been made to account for this as best as possible, the diffractometer upon which the data were collected lacks a camera with which to obtain a true representation of the crystal for a more explicit correction. The impact of this upon the data is further compounded by the crystal existing as a highly anisotropic plate. Errors carried through from this in the intensities will increase the residual levels in the model. This is in addition to the typical fourier rippling that is expected at heavy elements, and the impact of the spherical contraction of the valence shell owed to the ionisation of these metals in this highly charge system. Since these residuals exist close to these heavy metal centres and the model (and symmetry) has been thoroughly evaluated (see the manuscript), these are believed to have no physical significance.

```
PLAT972_ALERT_2_C Check Calcd Resid. Dens.  1.26Ang From Sr1           -1.67 eA-3
```

Author Response: The sample is highly absorbing and contains a significant proportion of heavy elements. While efforts have been made to account for this as best as possible, the diffractometer upon which the data were collected lacks a camera with which to obtain a true representation of the crystal for a more explicit correction. The impact of this upon the data is further compounded by the crystal existing as a highly anisotropic plate. Errors carried through from this in the intensities will increase the residual levels in the model. This is in addition to the typical fourier rippling that is expected at heavy elements, and the impact of the spherical contraction of the valence shell owed to the ionisation of these metals in this highly charge system. Since these residuals exist close to these heavy metal centres and the model (and symmetry) has been thoroughly evaluated (see the manuscript), these are believed to have no physical significance.

```
PLAT975_ALERT_2_C Check Calcd Resid. Dens.  0.60Ang From O3      .       0.97 eA-3
```

Author Response: The sample is highly absorbing and contains a significant proportion of heavy elements. While efforts have been made to account for this as best as possible, the diffractometer upon which the data were collected lacks a camera with which to obtain a true representation of the crystal for a more explicit correction. The impact of this upon the data is further compounded by the crystal existing as a highly anisotropic plate. Errors carried through from this in the intensities will increase the residual levels in the model. This is in addition to the typical fourier rippling that is expected at heavy elements, and the impact of the spherical contraction of the valence shell owed to the ionisation of these metals in this highly charge system. Since these residuals exist close to these heavy metal centres and the model (and symmetry) has been thoroughly evaluated (see the manuscript), these are believed to have no physical significance.

```
PLAT975_ALERT_2_C Check Calcd Resid. Dens.  0.67Ang From O4      .       0.94 eA-3
```

Author Response: The sample is highly absorbing and contains a significant proportion of heavy elements. While efforts have been made to account for this as best as possible, the diffractometer upon which the data were collected lacks a camera with which to obtain a true representation of the crystal for a more explicit correction. The impact of this upon the data is further compounded by the crystal existing as a highly anisotropic plate. Errors carried through from this in the intensities will increase the residual levels in the model. This is in addition to the typical fourier rippling that is expected at heavy elements, and the impact of the spherical contraction of the valence shell owed to the ionisation of these metals in this highly charge system. Since these residuals exist close to these heavy metal centres and the model (and symmetry) has been thoroughly evaluated (see the manuscript), these are believed to have no physical significance.

```
PLAT975_ALERT_2_C Check Calcd Resid. Dens.  0.87Ang From O2      .       0.54 eA-3
```

Author Response: The sample is highly absorbing and contains a significant proportion of heavy elements. While efforts have been made to account for this as best as possible, the diffractometer upon which the data were collected lacks a camera with which to obtain a true representation of the crystal for a more explicit correction. The impact of this upon the data is further compounded by the crystal existing as a highly anisotropic plate. Errors carried through from this in the intensities will increase the residual levels in the model. This is in addition to the typical fourier rippling that is expected at heavy elements, and the impact of the spherical contraction of the valence shell owed to the ionisation of these metals in this highly charge system. Since these residuals exist close to these heavy metal centres and the model (and symmetry) has been thoroughly evaluated (see the manuscript), these are believed to have no physical significance.

Alert level G

```
ABSMU01_ALERT_1_G  Calculation of _exptl_absorpt_correction_mu
                not performed for this radiation type.
PLAT004_ALERT_5_G Polymeric Structure Found with Maximum Dimension         2 Info
PLAT045_ALERT_1_G Calculated and Reported Z Differ by a Factor ...     0.250 Check
PLAT072_ALERT_2_G SHELXL First  Parameter in WGHT  Unusually Large      0.17 Report
PLAT110_ALERT_2_G ADDSYM Detects Potential Lattice Translation ...         ? Check
PLAT112_ALERT_2_G ADDSYM Detects New (Pseudo) Symm. Elem        B        100 %Fit
```

Author Response: The deviations from the high symmetry aristotype are low, manifesting as low intensity superstructure reflections and small deviations from the tetragonal aristotype. The model (and symmetry) has been thoroughly evaluated (please see the manuscript).

```
PLAT112_ALERT_2_G ADDSYM Detects New (Pseudo) Symm. Elem         A       100 %Fit
```

Author Response: The deviations from the high symmetry aristotype are low, manifesting as low intensity superstructure reflections and small deviations from the tetragonal aristotype. The model (and symmetry) has been thoroughly evaluated (please see the manuscript).

```
PLAT116_ALERT_2_G ADDSYM Included (Pseudo) Lattice Translation ...     Please Check
PLAT794_ALERT_5_G Tentative Bond Valency for Sn1       (IV)      .       4.19 Info
PLAT912_ALERT_4_G Missing # of FCF Reflections Above STh/L=  0.600         50 Note
PLAT933_ALERT_2_G Number of HKL-OMIT Records in Embedded .res File          5 Note
               0  0-10,   0  0 -4,   0  2  0,   1  1 -5,   2  0  0,
PLAT969_ALERT_5_G The 'Henn et al.' R-Factor-gap value ..........      4.473 Note
             Predicted wR2: Based on SigI**2  4.97 or SHELX Weight 21.54
```

```
  7 ALERT level A = Most likely a serious problem - resolve or explain
 15 ALERT level B = A potentially serious problem, consider carefully
 34 ALERT level C = Check. Ensure it is not caused by an omission or oversight
 12 ALERT level G = General information/check it is not something unexpected

  6 ALERT type 1 CIF construction/syntax error, inconsistent or missing data
 57 ALERT type 2 Indicator that the structure model may be wrong or deficient
  1 ALERT type 3 Indicator that the structure quality may be low
  1 ALERT type 4 Improvement, methodology, query or suggestion
  3 ALERT type 5 Informative message, check
```

It is advisable to attempt to resolve as many as possible of the alerts in all categories. Often the minor alerts point to easily fixed oversights, errors and omissions in your CIF or refinement strategy, so attention to these fine details can be worthwhile. In order to resolve some of the more serious problems it may be necessary to carry out additional measurements or structure refinements. However, the purpose of your study may justify the reported deviations and the more serious of these should normally be commented upon in the discussion or experimental section of a paper or in the "special_details" fields of the CIF. checkCIF was carefully designed to identify outliers and unusual parameters, but every test has its limitations and alerts that are not important in a particular case may appear. Conversely, the absence of alerts does not guarantee there are no aspects of the results needing attention. It is up to the individual to critically assess their own results and, if necessary, seek expert advice.

Publication of your CIF in IUCr journals

A basic structural check has been run on your CIF. These basic checks will be run on all CIFs submitted for publication in IUCr journals (*Acta Crystallographica*, *Journal of Applied Crystallography*, *Journal of Synchrotron Radiation*); however, if you intend to submit to *Acta Crystallographica Section C* or *E* or *IUCrData*, you should make sure that full publication checks are run on the final version of your CIF prior to submission.

Publication of your CIF in other journals

Please refer to the *Notes for Authors* of the relevant journal for any special instructions relating to CIF submission.

PLATON version of 19/12/2024; check.def file version of 19/12/2024

Datablock srsno_i19vt_15_420_2_pnab - ellipsoid plot

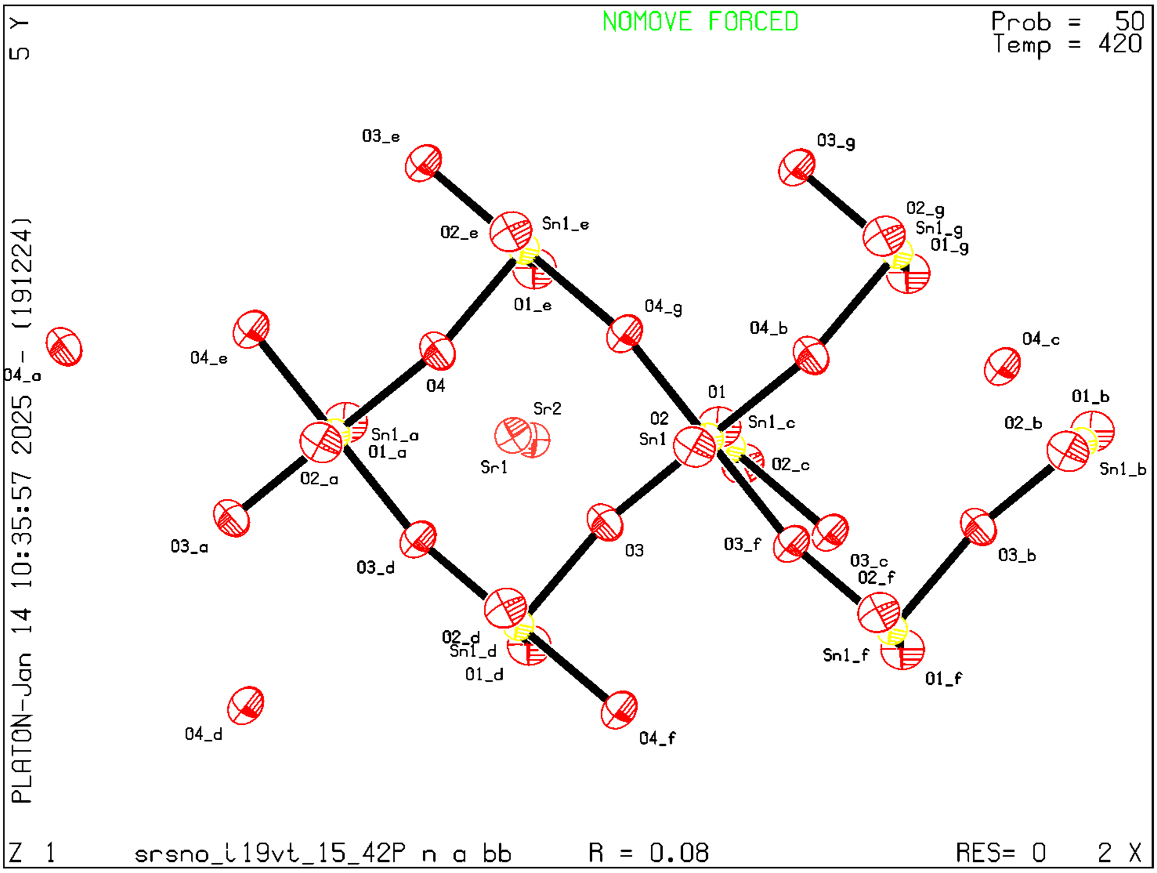

Supplement: Supplementary file 1 [file SrSnO_I19VT_15_420_2_pnab_checkcif.pdf › SSO_HP_VT_CIFs/SSO_VT_CIFS/SrSnO_I19VT_15_420_2_pnab_checkcif.pdf]
