# Supplementary material for: The varying temperature- and pressure-induced phase transition pathways in hybrid improper ferroelectric Sr3Sn2O7
Source: Acta Crystallogr B Struct Sci Cryst Eng Mater. 2025 Apr 14;81(Pt 3):318–24. doi: 10.1107/S2052520625002306 (PMC12147935; doi:10.1107/S2052520625002306)

checkCIF/PLATON report

Structure factors have been supplied for datablock(s) srsno_i19vt_24_430_2_pnab_if

THIS REPORT IS FOR GUIDANCE ONLY. IF USED AS PART OF A REVIEW PROCEDURE FOR PUBLICATION, IT SHOULD NOT REPLACE THE EXPERTISE OF AN EXPERIENCED CRYSTALLOGRAPHIC REFEREE.

No syntax errors found. CIF dictionary Interpreting this report

Datablock: srsno_i19vt_24_430_2_pnab_if

Bond precision: Sn- O = 0.0035 A Wavelength=0.68890

Cell:	a=5.6997(3)	b=5.7200(3)	c=20.6913(10)
	alpha=90	beta=90	gamma=90
Temperature:	430 K		

	Calculated	Reported
Volume	674.58(6)	674.58(6)
Space group	P n a b	P n a b
Hall group	-P 2bc 2n	-P 2bc 2n
Moiety formula	O28 Sn8, 12(Sr)	O7 Sn2 Sr3
Sum formula	O28 Sn8 Sr12	O7 Sn2 Sr3
Mr	2449.12	612.24
Dx,g cm-3	6.029	6.028
Z	1	4
Mu (mm-1)	28.105	28.442
F000	1080.0	1080.0
F000'	1061.72	
h,k,lmax	9,9,33	9,9,33
Nref	1534	1482
Tmin,Tmax		0.001,0.011
Tmin'		

Correction method= # Reported T Limits: Tmin=0.001 Tmax=0.011
AbsCorr = SPHERE

Data completeness= 0.966 Theta(max)= 34.171

R(reflections)= 0.0821(1097) wR2(reflections)= 0.2397(1482)

S = 1.011 Npar= 56

The following ALERTS were generated. Each ALERT has the format
test-name_ALERT_alert-type_alert-level.
Click on the hyperlinks for more details of the test.

Alert level A

PLAT971_ALERT_2_A Check Calcd Resid. Dens. 0.63Ang From Sn1 5.60 eA-3

Author Response: The sample is highly absorbing and contains a significant proportion of heavy elements. While efforts have been made to account for this as best as possible, the diffractometer upon which the data were collected lacks a camera with which to obtain a true representation of the crystal for a more explicit correction. The impact of this upon the data is further compounded by the crystal existing as a highly anisotropic plate. Errors carried through from this in the intensities will increase the residual levels in the model. This is in addition to the typical fourier rippling that is expected at heavy elements, and the impact of the spherical contraction of the valence shell owed to the ionisation of these metals in this highly charge system. Since these residuals exist close to these heavy metal centres and the model (and symmetry) has been thoroughly evaluated (see the manuscript), these are believed to have no physical significance.

PLAT971_ALERT_2_A Check Calcd Resid. Dens. 0.63Ang From Sn1 5.54 eA-3

Author Response: The sample is highly absorbing and contains a significant proportion of heavy elements. While efforts have been made to account for this as best as possible, the diffractometer upon which the data were collected lacks a camera with which to obtain a true representation of the crystal for a more explicit correction. The impact of this upon the data is further compounded by the crystal existing as a highly anisotropic plate. Errors carried through from this in the intensities will increase the residual levels in the model. This is in addition to the typical fourier rippling that is expected at heavy elements, and the impact of the spherical contraction of the valence shell owed to the ionisation of these metals in this highly charge system. Since these residuals exist close to these heavy metal centres and the model (and symmetry) has been thoroughly evaluated (see the manuscript), these are believed to have no physical significance.

PLAT971_ALERT_2_A Check Calcd Resid. Dens. 0.65Ang From Sn1 5.48 eA-3

Author Response: The sample is highly absorbing and contains a significant proportion of heavy elements. While efforts have been made to account for this as best as possible, the diffractometer upon which the data were collected lacks a camera with which to obtain a true representation of the crystal for a more explicit correction. The impact of this upon the data is further compounded by the crystal existing as a highly anisotropic plate. Errors carried through from this in the intensities will increase the residual levels in the model. This is in addition to the typical fourier rippling that is expected at heavy elements, and the impact of the spherical contraction of the valence shell owed to the ionisation of these metals in this highly charge system. Since these residuals exist close to these heavy metal centres and the model (and symmetry) has been thoroughly evaluated (see the manuscript), these are believed to have no physical significance.

```
PLAT971_ALERT_2_A Check Calcd Resid. Dens.  0.64Ang From Sn1            5.46 eA-3
```

Author Response: The sample is highly absorbing and contains a significant proportion of heavy elements. While efforts have been made to account for this as best as possible, the diffractometer upon which the data were collected lacks a camera with which to obtain a true representation of the crystal for a more explicit correction. The impact of this upon the data is further compounded by the crystal existing as a highly anisotropic plate. Errors carried through from this in the intensities will increase the residual levels in the model. This is in addition to the typical fourier rippling that is expected at heavy elements, and the impact of the spherical contraction of the valence shell owed to the ionisation of these metals in this highly charge system. Since these residuals exist close to these heavy metal centres and the model (and symmetry) has been thoroughly evaluated (see the manuscript), these are believed to have no physical significance.

```
PLAT971_ALERT_2_A Check Calcd Resid. Dens.  0.55Ang From Sr2            3.74 eA-3
```

Author Response: The sample is highly absorbing and contains a significant proportion of heavy elements. While efforts have been made to account for this as best as possible, the diffractometer upon which the data were collected lacks a camera with which to obtain a true representation of the crystal for a more explicit correction. The impact of this upon the data is further compounded by the crystal existing as a highly anisotropic plate. Errors carried through from this in the intensities will increase the residual levels in the model. This is in addition to the typical fourier rippling that is expected at heavy elements, and the impact of the spherical contraction of the valence shell owed to the ionisation of these metals in this highly charge system. Since these residuals exist close to these heavy metal centres and the model (and symmetry) has been thoroughly evaluated (see the manuscript), these are believed to have no physical significance.

```
PLAT971_ALERT_2_A Check Calcd Resid. Dens.  0.61Ang From Sr2            3.54 eA-3
```

Author Response: The sample is highly absorbing and contains a significant proportion of heavy elements. While efforts have been made to account for this as best as possible, the diffractometer upon which the data were collected lacks a camera with which to obtain a true representation of the crystal for a more explicit correction. The impact of this upon the data is further compounded by the crystal existing as a highly anisotropic plate. Errors carried through from this in the intensities will increase the residual levels in the model. This is in addition to the typical fourier rippling that is expected at heavy elements, and the impact of the spherical contraction of the valence shell owed to the ionisation of these metals in this highly charge system. Since these residuals exist close to these heavy metal centres and the model (and symmetry) has been thoroughly evaluated (see the manuscript), these are believed to have no physical significance.

```
PLAT971_ALERT_2_A Check Calcd Resid. Dens.  0.60Ang From Sr2            3.51 eA-3
```

Author Response: The sample is highly absorbing and contains a significant proportion of heavy elements. While efforts have been made to account for this as best as possible, the diffractometer upon which the data were collected lacks a camera with which to obtain a true representation of the crystal for a more explicit correction. The impact of this upon the data is further compounded by the crystal existing as a highly anisotropic plate. Errors carried through from this in the intensities will increase the residual levels in the model. This is in addition to the typical fourier rippling that is expected at heavy elements, and the impact of the spherical contraction of the valence shell owed to the ionisation of these metals in this highly charge system. Since these residuals exist close to these heavy metal centres and the model (and symmetry) has been thoroughly evaluated (see the manuscript), these are believed to have no physical significance.

```
PLAT972_ALERT_2_A Check Calcd Resid. Dens.  0.35Ang From Sr2           -4.73 eA-3
```

Author Response: The sample is highly absorbing and contains a significant proportion of heavy elements. While efforts have been made to account for this as best as possible, the diffractometer upon which the data were collected lacks a camera with which to obtain a true representation of the crystal for a more explicit correction. The impact of this upon the data is further compounded by the crystal existing as a highly anisotropic plate. Errors carried through from this in the intensities will increase the residual levels in the model. This is in addition to the typical fourier rippling that is expected at heavy elements, and the impact of the spherical contraction of the valence shell owed to the ionisation of these metals in this highly charge system. Since these residuals exist close to these heavy metal centres and the model (and symmetry) has been thoroughly evaluated (see the manuscript), these are believed to have no physical significance.

```
PLAT974_ALERT_2_A Check Calcd Negative Resid. Density on       Sr1      -3.43 eA-3
```

Author Response: The sample is highly absorbing and contains a significant proportion of heavy elements. While efforts have been made to account for this as best as possible, the diffractometer upon which the data were collected lacks a camera with which to obtain a true representation of the crystal for a more explicit correction. The impact of this upon the data is further compounded by the crystal existing as a highly anisotropic plate. Errors carried through from this in the intensities will increase the residual levels in the model. This is in addition to the typical fourier rippling that is expected at heavy elements, and the impact of the spherical contraction of the valence shell owed to the ionisation of these metals in this highly charge system. Since these residuals exist close to these heavy metal centres and the model (and symmetry) has been thoroughly evaluated (see the manuscript), these are believed to have no physical significance.

Alert level B

```
PLAT097_ALERT_2_B Large Reported Max.  (Positive) Residual Density       5.99 eA-3
```

Author Response: The sample is highly absorbing and contains a significant proportion of heavy elements. While efforts have been made to account for this as best as possible, the diffractometer upon which the data were collected lacks a camera with which to obtain a true representation of the crystal for a more explicit correction. The impact of this upon the data is further compounded by the crystal existing as a highly anisotropic plate. Errors carried through from this in the intensities will increase the residual levels in the model. This is in addition to the typical fourier rippling that is expected at heavy elements, and the impact of the spherical contraction of the valence shell owed to the ionisation of these metals in this highly charge system. Since the residual exists close to a heavy metal centre and the model (and symmetry) has been thoroughly evaluated (see the manuscript), it is believed to have no physical significance.

```
PLAT112_ALERT_2_B ADDSYM Detects New (Pseudo) Symm. Elem         4        100 %Fit
```

Author Response: The deviations from the high symmetry aristotype are low, manifesting as low intensity superstructure reflections and small deviations from the tetragonal aristotype. The model (and symmetry) has been thoroughly evaluated (please see the manuscript).

```
PLAT112_ALERT_2_B ADDSYM Detects New (Pseudo) Symm. Elem         m        100 %Fit
```

Author Response: The deviations from the high symmetry aristotype are low, manifesting as low intensity superstructure reflections and small deviations from the tetragonal aristotype. The model (and symmetry) has been thoroughly evaluated (please see the manuscript).

```
PLAT112_ALERT_2_B ADDSYM Detects New (Pseudo) Symm. Elem         m        100 %Fit
```

Author Response: The deviations from the high symmetry aristotype are low, manifesting as low intensity superstructure reflections and small deviations from the tetragonal aristotype. The model (and symmetry) has been thoroughly evaluated (please see the manuscript).

```
PLAT113_ALERT_2_B ADDSYM Suggests Possible Pseudo/New  Space Group     I4/mmm Check
              Check Model Parameter Symmetry for Reflection Data Support
```

Author Response: The deviations from the high symmetry aristotype are low, manifesting as low intensity superstructure reflections and small deviations from the tetragonal aristotype. The model (and symmetry) has been thoroughly evaluated (please see the manuscript).

```
PLAT971_ALERT_2_B Check Calcd Resid. Dens.  0.65Ang From Sr2            3.34 eA-3
```

Author Response: The sample is highly absorbing and contains a significant proportion of heavy elements. While efforts have been made to account for this as best as possible, the diffractometer upon which the data were collected lacks a camera with which to obtain a true representation of the crystal for a more explicit correction. The impact of this upon the data is further compounded by the crystal existing as a highly anisotropic plate. Errors carried through from this in the intensities will increase the residual levels in the model. This is in addition to the typical fourier rippling that is expected at heavy elements, and the impact of the spherical contraction of the valence shell owed to the ionisation of these metals in this highly charge system. Since these residuals exist close to these heavy metal centres and the model (and symmetry) has been thoroughly evaluated (see the manuscript), these are believed to have no physical significance.

```
PLAT971_ALERT_2_B Check Calcd Resid. Dens.  0.67Ang From Sr1            3.33 eA-3
```

Author Response: The sample is highly absorbing and contains a significant proportion of heavy elements. While efforts have been made to account for this as best as possible, the diffractometer upon which the data were collected lacks a camera with which to obtain a true representation of the crystal for a more explicit correction. The impact of this upon the data is further compounded by the crystal existing as a highly anisotropic plate. Errors carried through from this in the intensities will increase the residual levels in the model. This is in addition to the typical fourier rippling that is expected at heavy elements, and the impact of the spherical contraction of the valence shell owed to the ionisation of these metals in this highly charge system. Since these residuals exist close to these heavy metal centres and the model (and symmetry) has been thoroughly evaluated (see the manuscript), these are believed to have no physical significance.

```
PLAT971_ALERT_2_B Check Calcd Resid. Dens.  0.67Ang From Sr1            3.28 eA-3
```

Author Response: The sample is highly absorbing and contains a significant proportion of heavy elements. While efforts have been made to account for this as best as possible, the diffractometer upon which the data were collected lacks a camera with which to obtain a true representation of the crystal for a more explicit correction. The impact of this upon the data is further compounded by the crystal existing as a highly anisotropic plate. Errors carried through from this in the intensities will increase the residual levels in the model. This is in addition to the typical fourier rippling that is expected at heavy elements, and the impact of the spherical contraction of the valence shell owed to the ionisation of these metals in this highly charge system. Since these residuals exist close to these heavy metal centres and the model (and symmetry) has been thoroughly evaluated (see the manuscript), these are believed to have no physical significance.

`PLAT975_ALERT_2_B Check Calcd Resid. Dens.  0.53Ang From O4      .       1.68 eA-3`

Author Response: The sample is highly absorbing and contains a significant proportion of heavy elements. While efforts have been made to account for this as best as possible, the diffractometer upon which the data were collected lacks a camera with which to obtain a true representation of the crystal for a more explicit correction. The impact of this upon the data is further compounded by the crystal existing as a highly anisotropic plate. Errors carried through from this in the intensities will increase the residual levels in the model. This is in addition to the typical fourier rippling that is expected at heavy elements, and the impact of the spherical contraction of the valence shell owed to the ionisation of these metals in this highly charge system. Since these residuals exist close to these heavy metal centres and the model (and symmetry) has been thoroughly evaluated (see the manuscript), these are believed to have no physical significance.

`PLAT975_ALERT_2_B Check Calcd Resid. Dens.  0.56Ang From O3      .       1.64 eA-3`

Author Response: The sample is highly absorbing and contains a significant proportion of heavy elements. While efforts have been made to account for this as best as possible, the diffractometer upon which the data were collected lacks a camera with which to obtain a true representation of the crystal for a more explicit correction. The impact of this upon the data is further compounded by the crystal existing as a highly anisotropic plate. Errors carried through from this in the intensities will increase the residual levels in the model. This is in addition to the typical fourier rippling that is expected at heavy elements, and the impact of the spherical contraction of the valence shell owed to the ionisation of these metals in this highly charge system. Since these residuals exist close to these heavy metal centres and the model (and symmetry) has been thoroughly evaluated (see the manuscript), these are believed to have no physical significance.

Alert level C

```
DIFMN02_ALERT_2_C  The minimum difference density is < -0.1*ZMAX*0.75
            _refine_diff_density_min given =     -4.481
            Test value =     -3.750
DIFMN03_ALERT_1_C  The minimum difference density is < -0.1*ZMAX*0.75
            The relevant atom site should be identified.
DIFMX02_ALERT_1_C  The maximum difference density is > 0.1*ZMAX*0.75
            The relevant atom site should be identified.
PLAT041_ALERT_1_C Calc. and Reported SumFormula    Strings  Differ     Please Check
              Calc: O28 Sn8 Sr12
              Rep.: O7 Sn2 Sr3
PLAT042_ALERT_1_C Calc. and Reported MoietyFormula Strings  Differ     Please Check
              Calc: O28 Sn8, 12(Sr)
              Rep.: O7 Sn2 Sr3
PLAT051_ALERT_1_C Mu(calc) and Mu(cif) Ratio Differs from 1.0 by .       1.19 %
PLAT098_ALERT_2_C Large Reported Min.  (Negative) Residual Density      -4.48 eA-3
PLAT905_ALERT_3_C Negative K value in the Analysis of Variance ...     -0.921 Report
PLAT971_ALERT_2_C Check Calcd Resid. Dens.  0.53Ang From O4              1.68 eA-3
```

Author Response: The sample is highly absorbing and contains a significant proportion of heavy elements. While efforts have been made to account for this as best as possible, the diffractometer upon which the data were collected lacks a camera with which to obtain a true representation of the crystal for a more explicit correction. The impact of this upon the data is further compounded by the crystal existing as a highly anisotropic plate. Errors carried through from this in the intensities will increase the residual levels in the model. This is in addition to the typical fourier rippling that is expected at heavy elements, and the impact of the spherical contraction of the valence shell owed to the ionisation of these metals in this highly charge system. Since these residuals exist close to these heavy metal centres and the model (and symmetry) has been thoroughly evaluated (see the manuscript), these are believed to have no physical significance.

```
PLAT971_ALERT_2_C Check Calcd Resid. Dens.  0.56Ang From O3              1.64 eA-3
```

Author Response: The sample is highly absorbing and contains a significant proportion of heavy elements. While efforts have been made to account for this as best as possible, the diffractometer upon which the data were collected lacks a camera with which to obtain a true representation of the crystal for a more explicit correction. The impact of this upon the data is further compounded by the crystal existing as a highly anisotropic plate. Errors carried through from this in the intensities will increase the residual levels in the model. This is in addition to the typical fourier rippling that is expected at heavy elements, and the impact of the spherical contraction of the valence shell owed to the ionisation of these metals in this highly charge system. Since these residuals exist close to these heavy metal centres and the model (and symmetry) has been thoroughly evaluated (see the manuscript), these are believed to have no physical significance.

```
PLAT971_ALERT_2_C Check Calcd Resid. Dens.  0.60Ang From O4             1.61 eA-3
```

Author Response: The sample is highly absorbing and contains a significant proportion of heavy elements. While efforts have been made to account for this as best as possible, the diffractometer upon which the data were collected lacks a camera with which to obtain a true representation of the crystal for a more explicit correction. The impact of this upon the data is further compounded by the crystal existing as a highly anisotropic plate. Errors carried through from this in the intensities will increase the residual levels in the model. This is in addition to the typical fourier rippling that is expected at heavy elements, and the impact of the spherical contraction of the valence shell owed to the ionisation of these metals in this highly charge system. Since these residuals exist close to these heavy metal centres and the model (and symmetry) has been thoroughly evaluated (see the manuscript), these are believed to have no physical significance.

```
PLAT971_ALERT_2_C Check Calcd Resid. Dens.  0.62Ang From O3             1.54 eA-3
```

Author Response: The sample is highly absorbing and contains a significant proportion of heavy elements. While efforts have been made to account for this as best as possible, the diffractometer upon which the data were collected lacks a camera with which to obtain a true representation of the crystal for a more explicit correction. The impact of this upon the data is further compounded by the crystal existing as a highly anisotropic plate. Errors carried through from this in the intensities will increase the residual levels in the model. This is in addition to the typical fourier rippling that is expected at heavy elements, and the impact of the spherical contraction of the valence shell owed to the ionisation of these metals in this highly charge system. Since these residuals exist close to these heavy metal centres and the model (and symmetry) has been thoroughly evaluated (see the manuscript), these are believed to have no physical significance.

PLAT972_ALERT_2_C Check Calcd Resid. Dens. 1.59Ang From Sr1   -1.98 eA-3

Author Response: The sample is highly absorbing and contains a significant proportion of heavy elements. While efforts have been made to account for this as best as possible, the diffractometer upon which the data were collected lacks a camera with which to obtain a true representation of the crystal for a more explicit correction. The impact of this upon the data is further compounded by the crystal existing as a highly anisotropic plate. Errors carried through from this in the intensities will increase the residual levels in the model. This is in addition to the typical fourier rippling that is expected at heavy elements, and the impact of the spherical contraction of the valence shell owed to the ionisation of these metals in this highly charge system. Since these residuals exist close to these heavy metal centres and the model (and symmetry) has been thoroughly evaluated (see the manuscript), these are believed to have no physical significance.

PLAT972_ALERT_2_C Check Calcd Resid. Dens. 1.18Ang From O1   -1.91 eA-3

Author Response: The sample is highly absorbing and contains a significant proportion of heavy elements. While efforts have been made to account for this as best as possible, the diffractometer upon which the data were collected lacks a camera with which to obtain a true representation of the crystal for a more explicit correction. The impact of this upon the data is further compounded by the crystal existing as a highly anisotropic plate. Errors carried through from this in the intensities will increase the residual levels in the model. This is in addition to the typical fourier rippling that is expected at heavy elements, and the impact of the spherical contraction of the valence shell owed to the ionisation of these metals in this highly charge system. Since these residuals exist close to these heavy metal centres and the model (and symmetry) has been thoroughly evaluated (see the manuscript), these are believed to have no physical significance.

PLAT972_ALERT_2_C Check Calcd Resid. Dens. 1.26Ang From Sr1   -1.89 eA-3

Author Response: The sample is highly absorbing and contains a significant proportion of heavy elements. While efforts have been made to account for this as best as possible, the diffractometer upon which the data were collected lacks a camera with which to obtain a true representation of the crystal for a more explicit correction. The impact of this upon the data is further compounded by the crystal existing as a highly anisotropic plate. Errors carried through from this in the intensities will increase the residual levels in the model. This is in addition to the typical fourier rippling that is expected at heavy elements, and the impact of the spherical contraction of the valence shell owed to the ionisation of these metals in this highly charge system. Since these residuals exist close to these heavy metal centres and the model (and symmetry) has been thoroughly evaluated (see the manuscript), these are believed to have no physical significance.

```
PLAT972_ALERT_2_C Check Calcd Resid. Dens.  1.26Ang From Sr1           -1.89 eA-3
```

Author Response: The sample is highly absorbing and contains a significant proportion of heavy elements. While efforts have been made to account for this as best as possible, the diffractometer upon which the data were collected lacks a camera with which to obtain a true representation of the crystal for a more explicit correction. The impact of this upon the data is further compounded by the crystal existing as a highly anisotropic plate. Errors carried through from this in the intensities will increase the residual levels in the model. This is in addition to the typical fourier rippling that is expected at heavy elements, and the impact of the spherical contraction of the valence shell owed to the ionisation of these metals in this highly charge system. Since these residuals exist close to these heavy metal centres and the model (and symmetry) has been thoroughly evaluated (see the manuscript), these are believed to have no physical significance.

```
PLAT972_ALERT_2_C Check Calcd Resid. Dens.  0.81Ang From O2            -1.85 eA-3
```

Author Response: The sample is highly absorbing and contains a significant proportion of heavy elements. While efforts have been made to account for this as best as possible, the diffractometer upon which the data were collected lacks a camera with which to obtain a true representation of the crystal for a more explicit correction. The impact of this upon the data is further compounded by the crystal existing as a highly anisotropic plate. Errors carried through from this in the intensities will increase the residual levels in the model. This is in addition to the typical fourier rippling that is expected at heavy elements, and the impact of the spherical contraction of the valence shell owed to the ionisation of these metals in this highly charge system. Since these residuals exist close to these heavy metal centres and the model (and symmetry) has been thoroughly evaluated (see the manuscript), these are believed to have no physical significance.

```
PLAT972_ALERT_2_C Check Calcd Resid. Dens.  1.22Ang From Sr2           -1.72 eA-3
```

Author Response: The sample is highly absorbing and contains a significant proportion of heavy elements. While efforts have been made to account for this as best as possible, the diffractometer upon which the data were collected lacks a camera with which to obtain a true representation of the crystal for a more explicit correction. The impact of this upon the data is further compounded by the crystal existing as a highly anisotropic plate. Errors carried through from this in the intensities will increase the residual levels in the model. This is in addition to the typical fourier rippling that is expected at heavy elements, and the impact of the spherical contraction of the valence shell owed to the ionisation of these metals in this highly charge system. Since these residuals exist close to these heavy metal centres and the model (and symmetry) has been thoroughly evaluated (see the manuscript), these are believed to have no physical significance.

```
PLAT972_ALERT_2_C Check Calcd Resid. Dens.  1.33Ang From Sr1           -1.70 eA-3
```

Author Response: The sample is highly absorbing and contains a significant proportion of heavy elements. While efforts have been made to account for this as best as possible, the diffractometer upon which the data were collected lacks a camera with which to obtain a true representation of the crystal for a more explicit correction. The impact of this upon the data is further compounded by the crystal existing as a highly anisotropic plate. Errors carried through from this in the intensities will increase the residual levels in the model. This is in addition to the typical fourier rippling that is expected at heavy elements, and the impact of the spherical contraction of the valence shell owed to the ionisation of these metals in this highly charge system. Since these residuals exist close to these heavy metal centres and the model (and symmetry) has been thoroughly evaluated (see the manuscript), these are believed to have no physical significance.

```
PLAT972_ALERT_2_C Check Calcd Resid. Dens.  1.07Ang From Sr2           -1.70 eA-3
```

Author Response: The sample is highly absorbing and contains a significant proportion of heavy elements. While efforts have been made to account for this as best as possible, the diffractometer upon which the data were collected lacks a camera with which to obtain a true representation of the crystal for a more explicit correction. The impact of this upon the data is further compounded by the crystal existing as a highly anisotropic plate. Errors carried through from this in the intensities will increase the residual levels in the model. This is in addition to the typical fourier rippling that is expected at heavy elements, and the impact of the spherical contraction of the valence shell owed to the ionisation of these metals in this highly charge system. Since these residuals exist close to these heavy metal centres and the model (and symmetry) has been thoroughly evaluated (see the manuscript), these are believed to have no physical significance.

```
PLAT972_ALERT_2_C Check Calcd Resid. Dens.  1.35Ang From Sr1            -1.70 eA-3
```

Author Response: The sample is highly absorbing and contains a significant proportion of heavy elements. While efforts have been made to account for this as best as possible, the diffractometer upon which the data were collected lacks a camera with which to obtain a true representation of the crystal for a more explicit correction. The impact of this upon the data is further compounded by the crystal existing as a highly anisotropic plate. Errors carried through from this in the intensities will increase the residual levels in the model. This is in addition to the typical fourier rippling that is expected at heavy elements, and the impact of the spherical contraction of the valence shell owed to the ionisation of these metals in this highly charge system. Since these residuals exist close to these heavy metal centres and the model (and symmetry) has been thoroughly evaluated (see the manuscript), these are believed to have no physical significance.

```
PLAT972_ALERT_2_C Check Calcd Resid. Dens.  1.17Ang From O2             -1.69 eA-3
```

Author Response: The sample is highly absorbing and contains a significant proportion of heavy elements. While efforts have been made to account for this as best as possible, the diffractometer upon which the data were collected lacks a camera with which to obtain a true representation of the crystal for a more explicit correction. The impact of this upon the data is further compounded by the crystal existing as a highly anisotropic plate. Errors carried through from this in the intensities will increase the residual levels in the model. This is in addition to the typical fourier rippling that is expected at heavy elements, and the impact of the spherical contraction of the valence shell owed to the ionisation of these metals in this highly charge system. Since these residuals exist close to these heavy metal centres and the model (and symmetry) has been thoroughly evaluated (see the manuscript), these are believed to have no physical significance.

PLAT972_ALERT_2_C Check Calcd Resid. Dens. 1.23Ang From Sr2 -1.65 eA-3

Author Response: The sample is highly absorbing and contains a significant proportion of heavy elements. While efforts have been made to account for this as best as possible, the diffractometer upon which the data were collected lacks a camera with which to obtain a true representation of the crystal for a more explicit correction. The impact of this upon the data is further compounded by the crystal existing as a highly anisotropic plate. Errors carried through from this in the intensities will increase the residual levels in the model. This is in addition to the typical fourier rippling that is expected at heavy elements, and the impact of the spherical contraction of the valence shell owed to the ionisation of these metals in this highly charge system. Since these residuals exist close to these heavy metal centres and the model (and symmetry) has been thoroughly evaluated (see the manuscript), these are believed to have no physical significance.

PLAT972_ALERT_2_C Check Calcd Resid. Dens. 0.24Ang From Sn1 -1.62 eA-3

Author Response: The sample is highly absorbing and contains a significant proportion of heavy elements. While efforts have been made to account for this as best as possible, the diffractometer upon which the data were collected lacks a camera with which to obtain a true representation of the crystal for a more explicit correction. The impact of this upon the data is further compounded by the crystal existing as a highly anisotropic plate. Errors carried through from this in the intensities will increase the residual levels in the model. This is in addition to the typical fourier rippling that is expected at heavy elements, and the impact of the spherical contraction of the valence shell owed to the ionisation of these metals in this highly charge system. Since these residuals exist close to these heavy metal centres and the model (and symmetry) has been thoroughly evaluated (see the manuscript), these are believed to have no physical significance.

PLAT972_ALERT_2_C Check Calcd Resid. Dens. 1.58Ang From Sr2 -1.56 eA-3

Author Response: The sample is highly absorbing and contains a significant proportion of heavy elements. While efforts have been made to account for this as best as possible, the diffractometer upon which the data were collected lacks a camera with which to obtain a true representation of the crystal for a more explicit correction. The impact of this upon the data is further compounded by the crystal existing as a highly anisotropic plate. Errors carried through from this in the intensities will increase the residual levels in the model. This is in addition to the typical fourier rippling that is expected at heavy elements, and the impact of the spherical contraction of the valence shell owed to the ionisation of these metals in this highly charge system. Since these residuals exist close to these heavy metal centres and the model (and symmetry) has been thoroughly evaluated (see the manuscript), these are believed to have no physical significance.

```
PLAT972_ALERT_2_C Check Calcd Resid. Dens.  1.27Ang From O1            -1.53 eA-3
```

Author Response: The sample is highly absorbing and contains a significant proportion of heavy elements. While efforts have been made to account for this as best as possible, the diffractometer upon which the data were collected lacks a camera with which to obtain a true representation of the crystal for a more explicit correction. The impact of this upon the data is further compounded by the crystal existing as a highly anisotropic plate. Errors carried through from this in the intensities will increase the residual levels in the model. This is in addition to the typical fourier rippling that is expected at heavy elements, and the impact of the spherical contraction of the valence shell owed to the ionisation of these metals in this highly charge system. Since these residuals exist close to these heavy metal centres and the model (and symmetry) has been thoroughly evaluated (see the manuscript), these are believed to have no physical significance.

```
PLAT972_ALERT_2_C Check Calcd Resid. Dens.  1.31Ang From O1            -1.53 eA-3
```

Author Response: The sample is highly absorbing and contains a significant proportion of heavy elements. While efforts have been made to account for this as best as possible, the diffractometer upon which the data were collected lacks a camera with which to obtain a true representation of the crystal for a more explicit correction. The impact of this upon the data is further compounded by the crystal existing as a highly anisotropic plate. Errors carried through from this in the intensities will increase the residual levels in the model. This is in addition to the typical fourier rippling that is expected at heavy elements, and the impact of the spherical contraction of the valence shell owed to the ionisation of these metals in this highly charge system. Since these residuals exist close to these heavy metal centres and the model (and symmetry) has been thoroughly evaluated (see the manuscript), these are believed to have no physical significance.

PLAT972_ALERT_2_C Check Calcd Resid. Dens. 1.25Ang From Sr2 -1.51 eA-3

Author Response: The sample is highly absorbing and contains a significant proportion of heavy elements. While efforts have been made to account for this as best as possible, the diffractometer upon which the data were collected lacks a camera with which to obtain a true representation of the crystal for a more explicit correction. The impact of this upon the data is further compounded by the crystal existing as a highly anisotropic plate. Errors carried through from this in the intensities will increase the residual levels in the model. This is in addition to the typical fourier rippling that is expected at heavy elements, and the impact of the spherical contraction of the valence shell owed to the ionisation of these metals in this highly charge system. Since these residuals exist close to these heavy metal centres and the model (and symmetry) has been thoroughly evaluated (see the manuscript), these are believed to have no physical significance.

PLAT975_ALERT_2_C Check Calcd Resid. Dens. 0.81Ang From O4 . 1.29 eA-3

Author Response: The sample is highly absorbing and contains a significant proportion of heavy elements. While efforts have been made to account for this as best as possible, the diffractometer upon which the data were collected lacks a camera with which to obtain a true representation of the crystal for a more explicit correction. The impact of this upon the data is further compounded by the crystal existing as a highly anisotropic plate. Errors carried through from this in the intensities will increase the residual levels in the model. This is in addition to the typical fourier rippling that is expected at heavy elements, and the impact of the spherical contraction of the valence shell owed to the ionisation of these metals in this highly charge system. Since these residuals exist close to these heavy metal centres and the model (and symmetry) has been thoroughly evaluated (see the manuscript), these are believed to have no physical significance.

PLAT975_ALERT_2_C Check Calcd Resid. Dens. 0.79Ang From O3 . 1.27 eA-3

Author Response: The sample is highly absorbing and contains a significant proportion of heavy elements. While efforts have been made to account for this as best as possible, the diffractometer upon which the data were collected lacks a camera with which to obtain a true representation of the crystal for a more explicit correction. The impact of this upon the data is further compounded by the crystal existing as a highly anisotropic plate. Errors carried through from this in the intensities will increase the residual levels in the model. This is in addition to the typical fourier rippling that is expected at heavy elements, and the impact of the spherical contraction of the valence shell owed to the ionisation of these metals in this highly charge system. Since these residuals exist close to these heavy metal centres and the model (and symmetry) has been thoroughly evaluated (see the manuscript), these are believed to have no physical significance.

PLAT975_ALERT_2_C Check Calcd Resid. Dens. 0.70Ang From O2 . 0.75 eA-3

Author Response: The sample is highly absorbing and contains a significant proportion of heavy elements. While efforts have been made to account for this as best as possible, the diffractometer upon which the data were collected lacks a camera with which to obtain a true representation of the crystal for a more explicit correction. The impact of this upon the data is further compounded by the crystal existing as a highly anisotropic plate. Errors carried through from this in the intensities will increase the residual levels in the model. This is in addition to the typical fourier rippling that is expected at heavy elements, and the impact of the spherical contraction of the valence shell owed to the ionisation of these metals in this highly charge system. Since these residuals exist close to these heavy metal centres and the model (and symmetry) has been thoroughly evaluated (see the manuscript), these are believed to have no physical significance.

PLAT975_ALERT_2_C Check Calcd Resid. Dens. 0.67Ang From O1 . 0.68 eA-3

Author Response: The sample is highly absorbing and contains a significant proportion of heavy elements. While efforts have been made to account for this as best as possible, the diffractometer upon which the data were collected lacks a camera with which to obtain a true representation of the crystal for a more explicit correction. The impact of this upon the data is further compounded by the crystal existing as a highly anisotropic plate. Errors carried through from this in the intensities will increase the residual levels in the model. This is in addition to the typical fourier rippling that is expected at heavy elements, and the impact of the spherical contraction of the valence shell owed to the ionisation of these metals in this highly charge system. Since these residuals exist close to these heavy metal centres and the model (and symmetry) has been thoroughly evaluated (see the manuscript), these are believed to have no physical significance.

```
PLAT975_ALERT_2_C Check Calcd Resid. Dens.  0.99Ang From O1      .       0.58 eA-3
```

Author Response: The sample is highly absorbing and contains a significant proportion of heavy elements. While efforts have been made to account for this as best as possible, the diffractometer upon which the data were collected lacks a camera with which to obtain a true representation of the crystal for a more explicit correction. The impact of this upon the data is further compounded by the crystal existing as a highly anisotropic plate. Errors carried through from this in the intensities will increase the residual levels in the model. This is in addition to the typical fourier rippling that is expected at heavy elements, and the impact of the spherical contraction of the valence shell owed to the ionisation of these metals in this highly charge system. Since these residuals exist close to these heavy metal centres and the model (and symmetry) has been thoroughly evaluated (see the manuscript), these are believed to have no physical significance.

Alert level G

```
ABSMU01_ALERT_1_G  Calculation of _exptl_absorpt_correction_mu
                not performed for this radiation type.
PLAT004_ALERT_5_G Polymeric Structure Found with Maximum Dimension          2 Info
PLAT045_ALERT_1_G Calculated and Reported Z Differ by a Factor ...      0.250 Check
PLAT072_ALERT_2_G SHELXL First  Parameter in WGHT  Unusually Large       0.18 Report
PLAT110_ALERT_2_G ADDSYM Detects Potential Lattice Translation ...          ? Check
PLAT112_ALERT_2_G ADDSYM Detects New (Pseudo) Symm. Elem         B        100 %Fit
```

Author Response: The deviations from the high symmetry aristotype are low, manifesting as low intensity superstructure reflections and small deviations from the tetragonal aristotype. The model (and symmetry) has been thoroughly evaluated (please see the manuscript).

```
PLAT112_ALERT_2_G ADDSYM Detects New (Pseudo) Symm. Elem         A        100 %Fit
```

Author Response: The deviations from the high symmetry aristotype are low, manifesting as low intensity superstructure reflections and small deviations from the tetragonal aristotype. The model (and symmetry) has been thoroughly evaluated (please see the manuscript).

```
PLAT116_ALERT_2_G ADDSYM Included (Pseudo) Lattice Translation ...     Please Check
PLAT794_ALERT_5_G Tentative Bond Valency for Sn1       (IV)      .       4.20 Info
PLAT912_ALERT_4_G Missing # of FCF Reflections Above STh/L=  0.600         51 Note
PLAT933_ALERT_2_G Number of HKL-OMIT Records in Embedded .res File          4 Note
                0  0-10,   0  0 -4,   0  2  0,   1  1 -5,
PLAT969_ALERT_5_G The 'Henn et al.' R-Factor-gap value ..........      4.592 Note
              Predicted wR2: Based on SigI**2  5.22 or SHELX Weight 23.71
PLAT992_ALERT_5_G Repd & Actual _reflns_number_gt Values Differ by          2 Check
```

```
 9 ALERT level A = Most likely a serious problem - resolve or explain
10 ALERT level B = A potentially serious problem, consider carefully
33 ALERT level C = Check. Ensure it is not caused by an omission or oversight
13 ALERT level G = General information/check it is not something unexpected

 7 ALERT type 1 CIF construction/syntax error, inconsistent or missing data
52 ALERT type 2 Indicator that the structure model may be wrong or deficient
 1 ALERT type 3 Indicator that the structure quality may be low
 1 ALERT type 4 Improvement, methodology, query or suggestion
 4 ALERT type 5 Informative message, check
```

It is advisable to attempt to resolve as many as possible of the alerts in all categories. Often the minor alerts point to easily fixed oversights, errors and omissions in your CIF or refinement strategy, so attention to these fine details can be worthwhile. In order to resolve some of the more serious problems it may be necessary to carry out additional measurements or structure refinements. However, the purpose of your study may justify the reported deviations and the more serious of these should normally be commented upon in the discussion or experimental section of a paper or in the "special_details" fields of the CIF. checkCIF was carefully designed to identify outliers and unusual parameters, but every test has its limitations and alerts that are not important in a particular case may appear. Conversely, the absence of alerts does not guarantee there are no aspects of the results needing attention. It is up to the individual to critically assess their own results and, if necessary, seek expert advice.

Publication of your CIF in IUCr journals

A basic structural check has been run on your CIF. These basic checks will be run on all CIFs submitted for publication in IUCr journals (*Acta Crystallographica*, *Journal of Applied Crystallography*, *Journal of Synchrotron Radiation*); however, if you intend to submit to *Acta Crystallographica Section C* or *E* or *IUCrData*, you should make sure that full publication checks are run on the final version of your CIF prior to submission.

Publication of your CIF in other journals

Please refer to the *Notes for Authors* of the relevant journal for any special instructions relating to CIF submission.

PLATON version of 19/12/2024; check.def file version of 19/12/2024

Datablock srsno_i19vt_24_430_2_pnab_if - ellipsoid plot

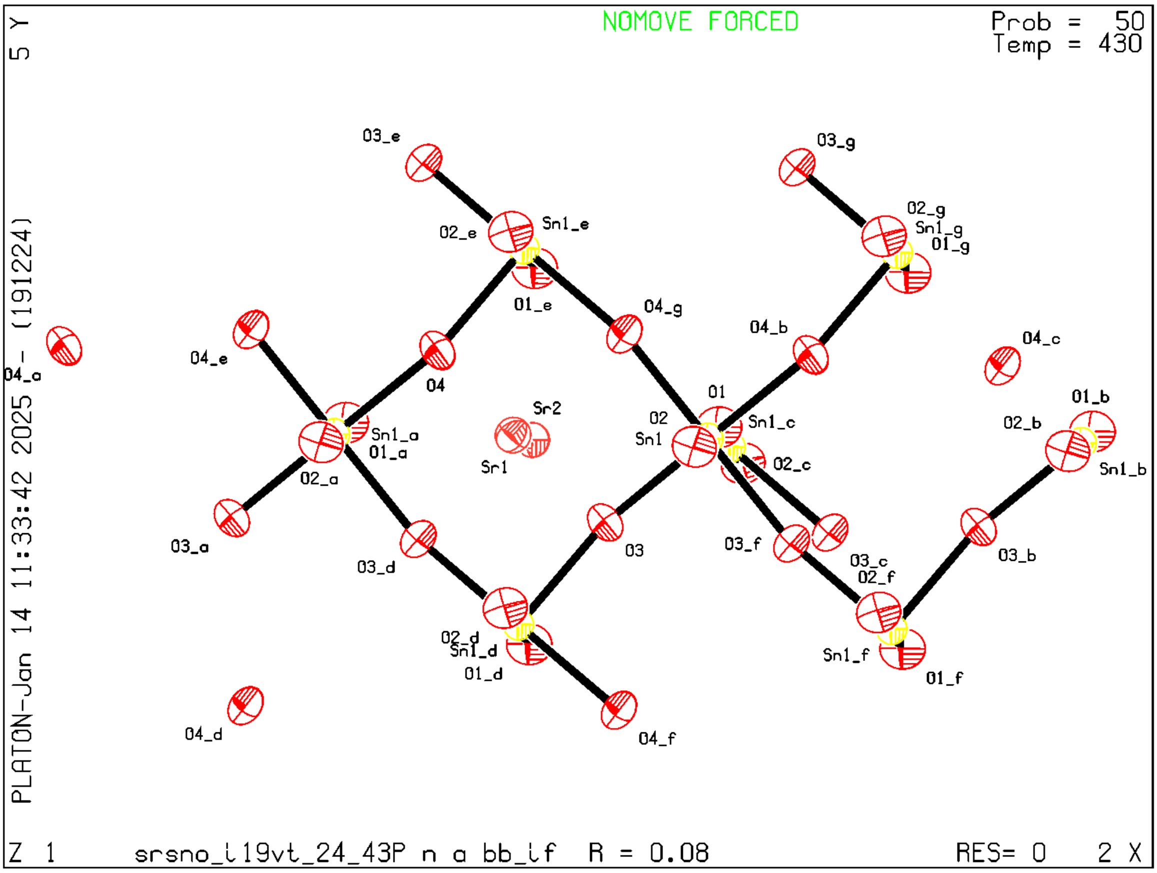

Supplement: Supplementary file 1 [file SrSnO_I19VT_24_430_2_pnab_if_checkcif.pdf › SSO_HP_VT_CIFs/SSO_VT_CIFS/SrSnO_I19VT_24_430_2_pnab_if_checkcif.pdf]
